# *CDKL1* variants affecting ciliary formation predispose to thoracic aortic aneurysm and dissection

**DOI:** 10.1172/JCI186287

**Published:** 2025-10-07

**Authors:** Theresa Nauth, Melanie Philipp, Sina Renner, Martin D. Burkhalter, Helke Schüler, Ceren Saygi, Kristian Händler, Bente Siebels, Alice Busch, Thomas Mair, Verena Rickassel, Sophia Deden, Konstantin Hoffer, Jakob Olfe, Thomas S. Mir, Yskert von Kodolitsch, Evaldas Girdauskas, Meike Rybczynski, Malte Kriegs, Hannah Voß, Thomas Sauvigny, Malte Spielmann, Malik Alawi, Susanne Krasemann, Christian Kubisch, Till J. Demal, Georg Rosenberger

**Affiliations:** 1 Institute of Human Genetics, University Medical Center Hamburg-Eppendorf, Hamburg, Germany.; 2 Department of Experimental and Clinical Pharmacology and Pharmacogenomics, Section of Pharmacogenomics, Eberhard-Karls-University Tübingen, Tübingen, Germany.; 3 Department of Cardiology, University Heart & Vascular Center Hamburg, Hamburg, Germany.; 4 Bioinformatics Core, University Medical Center Hamburg-Eppendorf, Hamburg, Germany.; 5 Institute of Human Genetics, Universitätsklinikum Schleswig-Holstein (UKSH), University of Lübeck and University of Kiel, Lübeck, Germany.; 6 Section Mass Spectrometry and Proteomics, Center for Diagnostics;; 7 University Cancer Center Hamburg (UCCH) Kinomics Core Facility, Hubertus Wald Tumorzentrum–UCCH; and; 8 Department of Radiotherapy and Radiation Oncology, Hubertus Wald Tumorzentrum–UCCH; University Medical Center Hamburg-Eppendorf, Hamburg, Germany.; 9 Clinic for Children’s Heart Medicine and Adult Congenital Heart Disease and; 10 Department of Cardiovascular Surgery, University Heart & Vascular Center Hamburg, Hamburg, Germany.; 11 Department of Neurosurgery, University Medical Center Hamburg-Eppendorf, Hamburg, Germany.; 12 Human Molecular Genetics Group, Max Planck Institute for Molecular Genetics, Berlin, Germany.; 13 DZHK e.V. (German Center for Cardiovascular Research), Partner Site Hamburg/Kiel/Lübeck, Germany.; 14 Institute of Neuropathology and; 15 Core Facility for Experimental Histo-Pathology, University Medical Center Hamburg-Eppendorf, Hamburg, Germany.

**Keywords:** Cell biology, Genetics, Vascular biology, Cardiovascular disease, Genetic diseases

## Abstract

Genetic factors are fundamental in the etiology of thoracic aortic aneurysm and dissection (TAAD), but the genetic cause is detected in only about 30% of cases. To define unreported TAAD-associated sequence variants, exome and gene panel sequencing was performed in 323 patients. We identified heterozygous *CDKL1* variants [c.427T>C p.(Cys143Arg), c.617C>T p.(Ser206Leu), and c.404C>T p.(Thr135Met)] in 6 patients from 3 families with TAAD spectrum disorders. *CDKL1* encodes a protein kinase involved in ciliary biology. Amino acid substitutions were predicted to affect CDKL1 catalytic activity or protein binding properties. CDKL1 was expressed in vascular smooth muscle cells in normal and diseased human aortic wall tissue. Cdkl1 knockdown and transient knockout in zebrafish resulted in intersomitic vessel (ISV) malformations and aortic dilation. Coinjection of human *CDKL1^wild-type^* RNA, but not *CDKL1^Cys143Arg^* and *CDKL1^Ser206Leu^* RNA, rescued ISV malformations. All variants affected CDKL1 kinase function and profiling data, and altered protein-protein binding properties, particularly with ciliary transport molecules. Expression of CDKL1 variants in heterologous cells interfered with cilia formation and length, CDKL1 localization, and p38 MAPK and Vegf signaling. Our data suggest a role of *CDKL1* variants in the pathogenesis of TAAD spectrum disorders. The association between primary cilia dysregulation and TAAD expands our knowledge of the underlying molecular pathophysiology.

## Abstract 2

Collected data support that cilia-associated kinase *CDKL1* is a new disease gene for aortic disease and dysfunction of primary cilia is a new pathomechanism for aortopathies.

## Graphical abstract

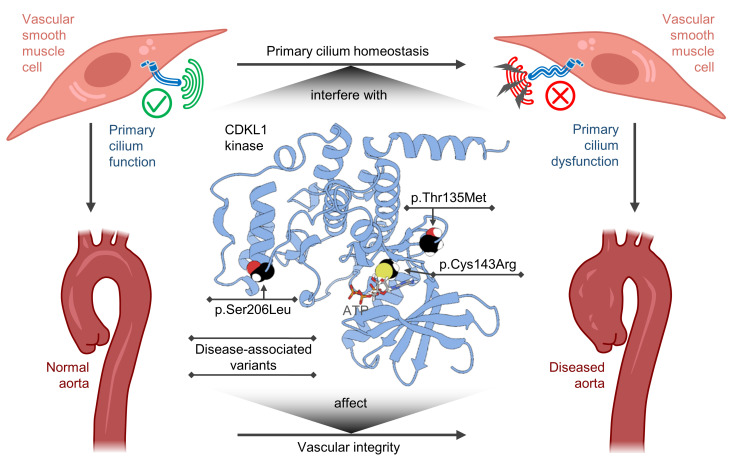

## Introduction

Thoracic aortic aneurysms and dissections (TAAD) are a major cause of morbidity and mortality in developed countries (1–3). TAAD is divided into non-syndromic forms and syndromic forms such as Marfan syndrome and Loeys-Dietz syndrome. Patients with syndromic forms present with anomalies within various organ systems including integumental, musculoskeletal, ocular, craniofacial, and/or non-aortic vascular manifestations (2, 4). In a larger context, diseases with TAAD can be counted among the extensive group of connective tissue disorders (CTDs) (5). From an etiological point of view, TAAD is a heterogeneous disease with a high proportion of genetic predisposition (1, 2, 6). Accordingly, a positive family history is a substantial risk factor for TAAD (2). More than 40 genes with differing strength of association with hereditary TAAD (HTAAD) have been described (1, 4, 7). However, definitively disease-causing variants in these genes are only found in less than 30% of patients (2, 4), which suggests further genetic heterogeneity (4). As a genetic diagnosis is getting increasingly important for gene-tailored, personalized patient management, further disease genes have to be identified.

Most known genes implicated in HTAAD encode proteins acting in 3 cellular mechanisms: (a) extracellular matrix (ECM) assembly, maintenance, and homeostasis (including collagen metabolism); (b) transforming growth factor-β (TGFB) signaling; and (c) vascular smooth muscle cell (VSMC) function (1–3). To give some details, fibrillins and collagens (e.g., FBN1, COL3A1) are structural ECM components; enzymes such as LOX and PLOD1 are involved in processing of structural ECM molecules; TGFB receptors and SMAD proteins (e.g., TGFBR1, SMAD3) mediate or affect TGFB signaling and VSMC phenotype; and MYH11, ACTA2, MYLK, and PRKG1 are regulators of VSMC contractility (1, 2, 4). Few HTAAD genes are associated with miscellaneous cellular mechanisms such as cell-cell adhesion or transcriptional regulation (1, 3, 4). To identify additional susceptibility genes for TAAD, we performed exome sequencing in a family with TAAD. We validated our priority candidate genes by genetic analyses in a cohort of 320 individuals with TAAD spectrum disorders/hereditary CTDs, by computer-aided in silico analysis, and by extensive functional characterization of candidate variants.

## Results

### Identification of heterozygous CDKL1 missense variants in patients with TAAD spectrum disorders.

**Figure 1:**
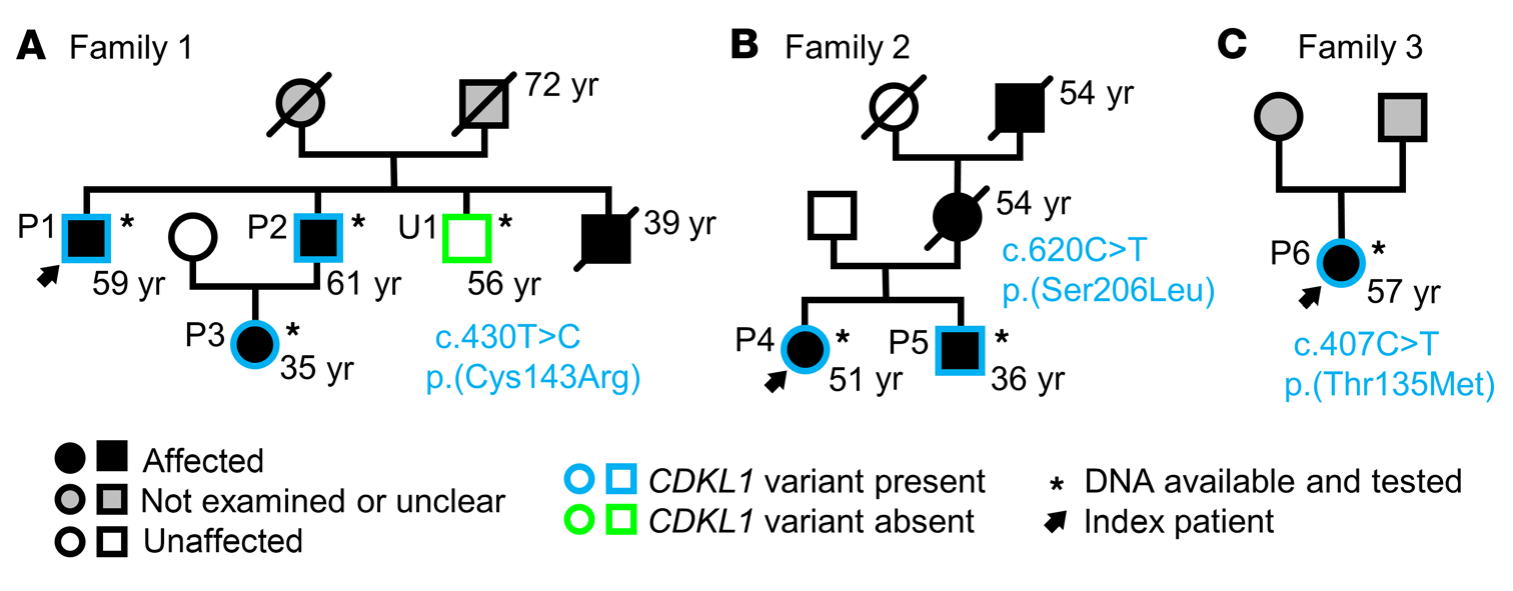
Family pedigrees. The respective identified *CDKL1* variant and current age (in years) is given. (**A**) Family 1. Patients P1, P2, and P3 with a TAAD spectrum disorder were used for the identification of *CDKL1* as candidate disease gene. U1, unaffected brother of the index. (**B**) Family 2. Patients P4 and P5 with Marfan syndrome–like features. (**C**) Family 3. Patient P6 with aortic dissection.

We performed exome sequencing in 3 affected individuals of family 1 (Figure 1A). The male index patient (patient P1) had a severe and complex aortic disease, which affected the entire length of the organ. Initially, he presented at an external hospital with a type B aortic dissection, which was treated by a replacement of the abdominal aorta and iliac artery replacement using a Y-graft, with reimplantation of the renal and visceral arteries at the age of 45 years. Subsequently, due to a thoracic aortic aneurysm of the descending aorta, a thoracic endovascular aortic repair of the distal aortic arch as well as the descending aorta was performed at our center at the same year. Thereafter, a type A dissection occurred, which necessitated the replacement of the aortic root (Bentall procedure) and the proximal arch, also within the same year. Due to graft infections and an endoleak, several further open and endovascular aortic procedures were necessary afterward. In addition to the aortic disease, a physical examination at the age of 52 years revealed hypertelorism, a high-arched palate, flat feet (pes planus), and severe myopia. His brother (patient P2) had a type B aortic dissection at the age of 52 years, which was treated by endovascular aortic repair. An anterior spinal artery syndrome led to a spinal cord injury. At the age of 53 years, carotid artery dissections on both sides on the basis of carotidal aneurysms and basal ganglia hemorrhage occurred. The index patient’s niece (patient P3) presented at the age of 22 with scoliosis, positive wrist and thumb sign, planovalgus deformity of both feet, myopia, greater than 90° extension of the fifth digit, borderline dilated pulmonary artery, and a marfanoid habitus. The aorta showed normal diameters and was not affected by dissections. Table 1 summarizes the clinical manifestations. Family history indicated that an older brother of the index patient died due to an aortic event (TAAD) at the age of 39 years. None of the 3 affected family members showed sequence variants with allele frequencies (AFs) ≤0.05 in disease and candidate disease genes for vascular/connective tissue disorders (Supplemental Table 1; supplemental material available online with this article; https://doi.org/10.1172/JCI186287DS1). Moreover, they did not share putatively disease-relevant copy number variations, and genome sequencing in patient P1 did not reveal any rare intronic *FBN1* variants predicted to affect splicing (Supplemental Data: Results). Filtering of exome data, variant prioritization, and genotyping in the unaffected brother (Figure 1A, U1) of the index patient resulted in a list of 11 putative candidate alleles present in a heterozygous form in all 3 affected family members (Table 2). Further variant prioritization including pathogenicity predictions, allele frequencies, and functional considerations supported the heterozygous missense variant *CDKL1* c.427T>C p.(Cys143Arg) as the most promising candidate allele (Table 2): First, CDKL1 (cyclin-dependent kinase–like 1) belongs to a family of CDKL kinases encompassing homologs CDKL1, CDKL2, CDKL3, CDKL4, and CDKL5 (8). Little is known about CDKL function, except for *CDKL5* that is encoded by the disease gene for a form of epileptic encephalopathy (developmental and epileptic encephalopathy-2 [DEE2], MIM #300672). Pathogenic variants affecting CDKL5 amino acid 152 (p.Cys152Arg, p.Cys152Phe, p.Cys152Tyr), which is homologous to Cys^143^ in CDKL1, have been reported previously (9–13). Second, allele count of *CDKL1* c.427T>C is 0 in the Genome Aggregation Database (gnomAD) v2.1.1 (Table 2) (14). Third, CDKL1 p.(Cys143Arg) was predicted to be damaging by 5 in silico pathogenicity prediction tools (Table 2), and CDKL1 Cys^143^ was determined to be crucial for ATP binding by the use of structural bioinformatics approaches (15).

Gene panel sequencing identified 2 further CDKL1 variants in a cohort of 320 consecutive, unrelated individuals with TAAD spectrum or connective tissue disorders: The heterozygous missense variant *CDKL1* c.617C>T p.(Ser206Leu) was detected in patient P4 (family 2, Figure 1B), a woman with Marfan syndrome–like features including positive wrist and thumb signs, dural ectasia, pectus excavatum, and joint hyperflexibility (knees and elbows) as first reported at the age of 37 years and confirmed in her most recent clinical examination at the age of 51 years (Table 1). Her brother, patient P5 (Figure 1B), also carries the *CDKL1* c.617C>T variant and presented with scoliosis, positive thumb sign, greater than 90° extension of the fifth digit, reduced upper segment/lower segment ratio, increased arm/height ratio (dolichostenomelia), and dural ectasia at the age of 23 years and at his most recent clinical examination at the age of 34 years. According to medical history, the mother and the grandfather of the siblings died of an aortic dissection and sudden cardiac death, respectively (Figure 1B). We did not identify variants with AF ≤0.05 in disease and candidate disease genes for vascular/connective tissue disorders (Supplemental Table 1). The siblings did not share potentially pathogenic copy number variations, and genome sequencing in patient P5 did not reveal any rare intronic *FBN1* variants predicted to affect splicing (Supplemental Data: Results). The AF of *CDKL1* c.617C>T p.(Ser206Leu) is 0.00001831 in gnomAD (v2.1.1) controls, 0.00004576 for non-Finnish Europeans (NFEs) in gnomAD (v4.1.0), and 0.00003656 in gnomAD (v4.1.0) total dataset (Table 2). CDKL1 p.(Ser206Leu) is predicted to be deleterious by all 5 tested pathogenicity classifiers (Table 2). Moreover, DEE2-causing serine-to-arginine change of CDKL5 amino acid 215, which is homologous to CDKL1 Ser^206^, caused a marked reduction in CDKL5 kinase activity in cell-based studies (16). Segregation analysis was not possible because DNA samples from further family members were not available.

Patient P6 (family 3, Figure 1C), a 57-year-old woman, presented with an acute type A aortic dissection from the aortic root to the iliac bifurcation at the age of 52. The brachiocephalic trunk originated from the true lumen; the left common carotid artery and the left subclavian artery were both also affected by the dissection. She was treated with a replacement of the ascending aorta and the proximal aortic arch as well as the aortic valve using a mechanical aortic valve prosthesis. The most recent clinical examination at the age of 52 years did not show any relevant musculoskeletal features, other than pes planus (Table 1). Her family history was negative for aortic disease. Gene panel sequencing revealed the heterozygous missense variant *CDKL1* c.404C>T, which has an AF of 0.00004570 in gnomAD (v2.1.1) controls, 0.00009407 for NFEs in gnomAD (v4.1.0), and 0.00007930 in gnomAD (v4.1.0) total dataset (Table 2). CDKL1 p.(Thr135Met) is predicted to be deleterious by 4 of 5 tested pathogenicity classifiers (Table 2). In addition, we identified 2 heterozygous variants in OMIM disease genes in patient P6: *MYLK* c.3064C>A p.(Pro1022Thr) and *FLNC* c.449A>G p.(Asp150Gly), which were classified as variant of uncertain significance (VUS) and likely benign variant (LBV), respectively (Table 2) (17). *MYLK* c.3064C>A p.(Pro1022Thr) is listed neither in the Single Nucleotide Polymorphism Database (dbSNP) and gnomAD database (v2.1.1), nor in the Human Gene Mutation Database (HGMD) (18), and all pathogenicity scores tested were below their respective thresholds (Table 2). Heterozygous pathogenic variants in *MYLK* cause autosomal dominant aortic aneurysm, familial thoracic 7 (MIM #613780). The overwhelming majority of aortopathy-associated *MYLK* variants are frameshift, nonsense, or splice site alterations (ClinVar, HGMD) (18, 19), whereas the significance of *MYLK* missense variants for the manifestation of TAAD is unsolved (20, 21). Heterozygous pathogenic *FLNC* variants cause several forms of (cardio)myopathies (MIM #617047, #614065, #609524), none of which are consistent with the clinical manifestations in patient P6. *FLNC* c.449A>G p.(Asp150Gly) has an AF of 0.00003672 in gnomAD (v2.1.1) controls (Table 2), and it is listed in ClinVar and HGMD as VUS and likely disease-causing, respectively, for (cardio)myopathies (22, 23). Only 2 of 5 pathogenicity scores calculated for *FLNC* c.449A>G p.(Asp150Gly) were above the respective thresholds (Table 2). Parental DNA samples for segregation analysis were not available. Taken together, available data do not rule out a role for *MYLK* c.3064C>A and do not support a role for *FLNC* c.449A>G in the clinical manifestation of patient P6.

In summary, we identified 3 different *CDKL1* variants in 6 individuals from 3 families with clinical presentations suggestive of CTDs with aortic/arterial manifestations. The mean age of the patients at first clinical manifestation was 38.5 ± 12.4 years with an even female to male ratio. Four of six patients presented with vascular manifestations, and all but one (patient P2) showed skeletal features.

### Molecular modeling and conservation analysis.

**Figure 2:**
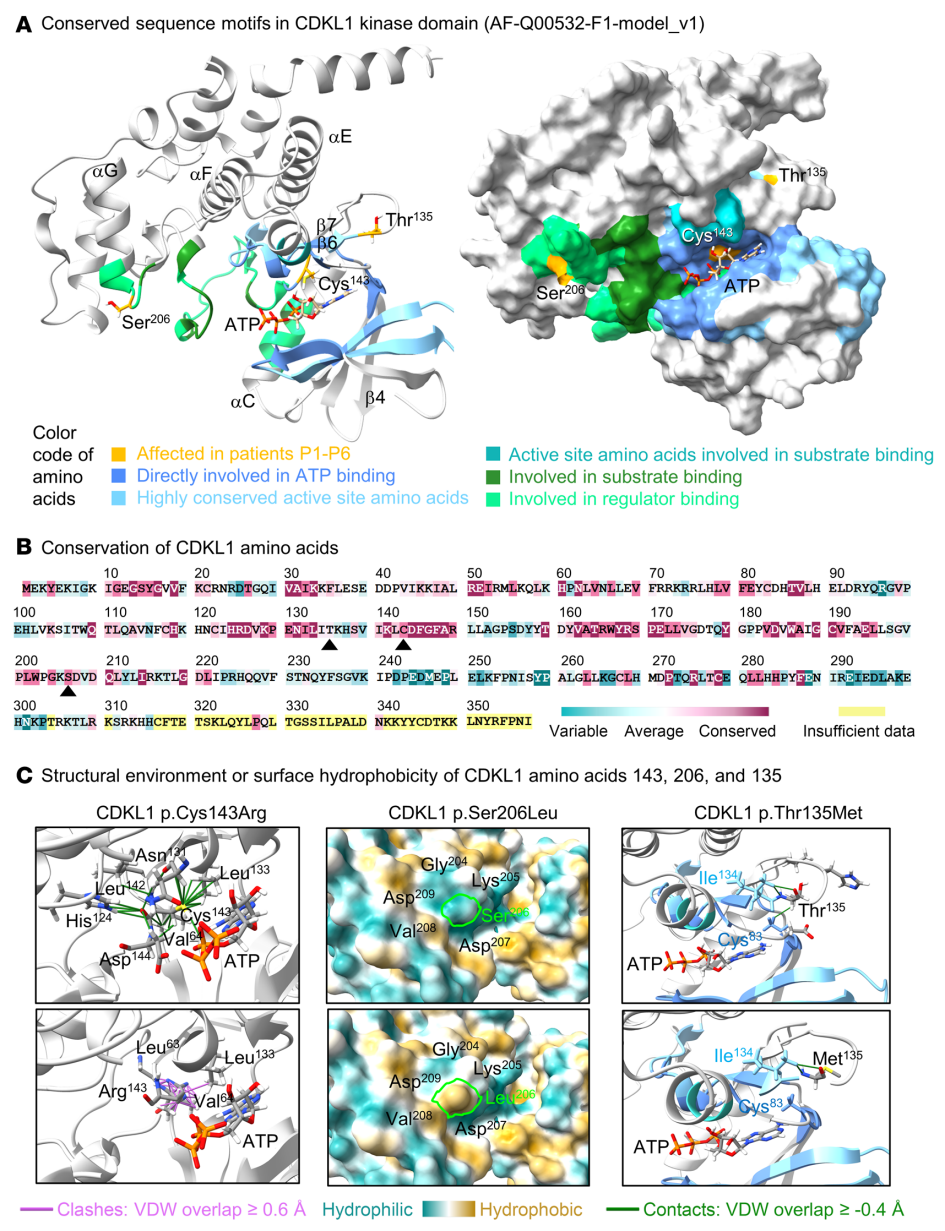
Structural and physicochemical impact of CDKL1 amino acid changes. (**A**) Molecular position of CDKL1 amino acids Cys^143^, Ser^206^, and Thr^135^ in relation to conserved sequence motifs and accessible surfaces. CDKL1 amino acids 1–303 are shown (AlphaFold AF-Q00532-F1-model_v4; AlphaFill AF-Q00532-F1-model_v1). Different colors in the ribbon representation (left model) and on the protein surface (right model) indicate conserved sequence motifs in the protein kinase domain of CDKL1 (amino acids 5–288). Secondary structure elements including β4, β6, and β7 strands as well as αC, αE, αF, and αG helices are indicated. (**B**) Conservation of CDKL1. Conservation was determined between 150 sequences among various species and visualized using the ConSurf server. Conserved amino acids are highlighted in maroon and variable residues in cyan. Cys^143^, Ser^206^, and Thr^135^ are marked with arrowheads. (**C**) Structural or physicochemical consequences of CDKL1 amino acid changes. Ribbon representations show spatial positions of CDKL1 wild-type amino acids Cys^143^ and Thr^135^ (top models) and exchanged amino acids Arg^143^ and Met^135^ (bottom models) as well as neighboring amino acids and phosphate donor ATP (all as sticks). Van der Waals (VDW) overlaps ≥0.4 Å (i.e., contacts) are shown as green lines; VDW overlaps ≥0.6 Å (i.e., clashes) are indicated by magenta lines. Side chains are colored by element (hydrogen: white; carbon: gray; oxygen: red; nitrogen: blue; sulfur: yellow; phosphorus: orange). In the ribbon representations for the p.Thr135Met change, amino acids involved in ATP positioning and protein catalysis are indicated by dark blue and light blue, respectively. Protein surface models show surface hydrophobicity of wild-type amino acid Ser^206^ (top model) and replaced amino acid Leu^206^ (bottom model), both within the putative Gly-Lys-Ser-Asp-Val-Asp protein binding motif. The color code ranges from cyan for the most hydrophilic residues to tan for the most hydrophobic residues. Comprehensive models are shown in Supplemental Figures 2–4.

Figure 2A shows the spatial position of affected CDKL1 amino acids Cys^143^, Ser^206^, and Thr^135^ in relation to conserved protein motifs, secondary structure elements, and protein surface. These amino acids are conserved (Figure 2B). Cys^143^ is the first amino acid of the highly conserved activation loop and borders the invariant DFG motif that is important for Mg^2+^ binding and protein catalysis (Figure 2, A and B, and Supplemental Figure 1). Molecular replacement of Cys^143^ with an arginine is predicted to result in loss of one van der Waals contact with the phosphate donor ATP and changes of more than 25 intramolecular interactions (Figure 2C and Supplemental Figure 2). We conclude that p.Cys143Arg may interfere with ATP phosphate positioning and/or protein catalysis. Ser^206^ is within the GKSDVD motif that is implicated in regulator/substrate binding (Figure 2, A and B, and Supplemental Figure 1). Replacement of the uncharged, polar, hydrophilic Ser^206^ by an uncharged, nonpolar, hydrophobic leucine is predicted to reverse surface hydrophobicity (Figure 2C and Supplemental Figure 3) that is crucial for protein folding and binding properties. Thus, the CDKL1 p.Ser206Leu alteration may affect interactions with yet undescribed regulators/substrates. Thr^135^ localizes in the Mg^2+^ binding loop and borders the serine/threonine protein kinases active-site signature, which suggests a structural function in positioning of Mg^2+^ and ATP (Figure 2A and Supplemental Figure 1). Molecular replacement of Thr^135^ is predicted to result in loss of more than 10 intramolecular interactions, including the one with Cys^83^ that is involved in ATP positioning and protein catalysis (Figure 2C and Supplemental Figures 1 and 4). Thus, p.Thr135Met possibly affects the catalytic activity of the kinase. In summary, structural and physicochemical in silico calculations suggest adverse effects on specific protein features for all identified CDKL1 variants. Detailed information on molecular modeling and on general kinase biology, including citations of relevant literature, is outlined in Supplemental Results, Supplemental Methods, and Supplemental Figures 1–4.

### CDKL1 is expressed in VSMCs within aortic wall tissue.

**Figure 3:**
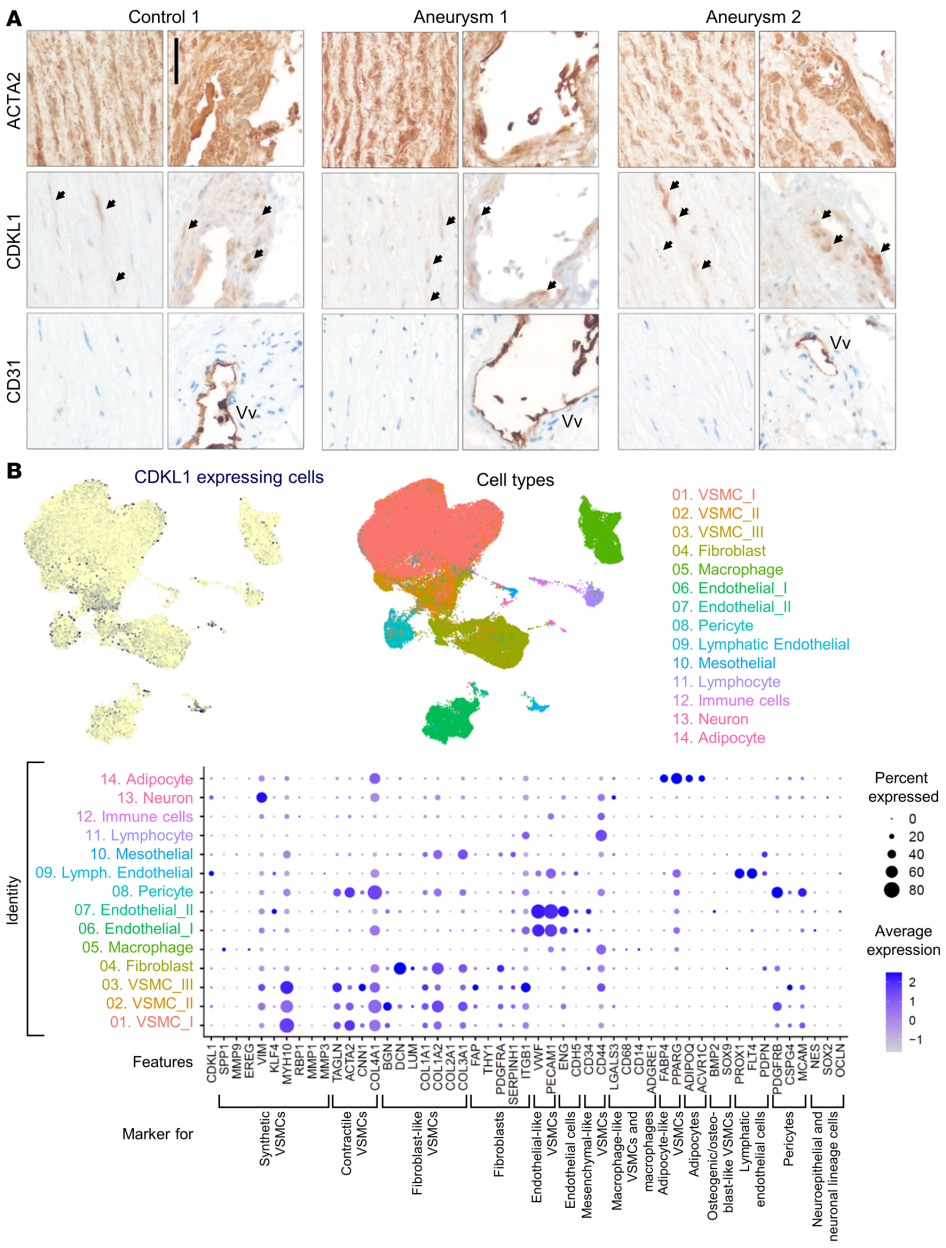
CDKL1 is expressed in VSMCs of normal and diseased aortic tissue. (**A**) Immunohistological analysis of CDKL1 expression in aortic wall tissue. Consecutive sections of formalin-fixed paraffin-embedded human aorta tissue from a control individual (control 1) and 2 patients with aortic aneurysms (aneurysm 1 and aneurysm 2) were immunostained for VSMC marker protein ACTA2 (smooth muscle actin), CDKL1 (antibody 1 [AB1; see Supplemental Data: Results]; 1:300), and the vascular endothelial marker CD31 using specific primary antibodies and horseradish peroxidase–conjugated (brown) secondary antibodies. Specimens were counterstained with hematoxylin (nuclei, blue). Representative pictures are shown. Note that CDKL1 is colocalizing with ACTA2-positive cells. Arrows indicate cells with stronger CDKL1 expression. Vv, vasa vasorum. Scale bar: 50 μm. (**B**) Reanalysis of combined publicly available human aortic single-cell RNA-Seq datasets (28, 29). Filtered and annotated data were merged and integrated using Harmony. Top left panel: Feature plot of CDKL1 expression in combined dataset. Each cell in the dataset is represented by a dot in the uniform manifold approximation and projection (UMAP) space. Cells with a CDKL1 expression above 0 are colored in blue; cells with no detected expression of CDKL1 are colored in yellow. Top right panel: UMAP representation of the annotation of the combined dataset. Annotation was copied and combined from original publications (28, 29). Bottom panel: Dot plot of marker genes confirming cell identity. Gene markers for specific cell types were selected from literature (25–27).

Immunohistological staining of tissue sections suggested CDKL1 expression in epithelial and endothelial cells, adipocytes, and melanocytes and in the walls of blood vessels of various tissues (Supplemental Figure 5) (24); however, no expression data were generated by immunohistochemistry (IHC) for larger vessels. We visualized CDKL1 in aortic tissue from 3 individuals with ascending aortic aneurysms and 2 control individuals (Figure 3A and Supplemental Figure 6, A and B). Clinical details regarding these patients and controls are given in Supplemental Methods. Staining of ACTA2 and CD31, marker proteins for smooth muscle cells and vascular endothelial cells, respectively, in consecutive sections suggested that CDKL1 is expressed weakly to moderately in VSMCs of the aortic media and moderately in the smooth muscle layer of small blood vessels, the vasa vasorum (Figure 3A and Supplemental Figure 6, A and B). Certain cells and cell layers showed stronger CDKL1 expression in both tissue areas (arrows in Figure 3A and in Supplemental Figure 6, A and B). Validation of CDKL1 antibodies is shown in Supplemental Figure 7.

Recent studies indicated that VSMCs display a high degree of plasticity and possess several differentiation potentials and phenotypes with changes in morphology and function (25–27). Therefore, and to support our IHC data, we assessed in which VSMC subtypes CDKL1 may be expressed by accessing 2 publicly available single-cell RNA-Seq datasets of aortic wall cells (28, 29). CDKL1 showed low but ubiquitous expression across all cell types present in the datasets (Figure 3B). Cell types showing slightly enriched CDKL1 expression as compared with general CDKL1 expression level in the dataset include lymphatic endothelial cells, neuronal cells, and VSMC subtype II (Figure 3B). The 3 defined VSMC subpopulations (I, II, and III) in our study consist primarily of contractile VSMCs according to coexpressed markers (specific markers: *TAGLN*, *ACTA2*, *COL4A1*); markers for synthetic VSMCs are also enriched (*VIM*, *MYH10*) (Figure 3B, dot plot) (25, 26, 30–32). VSMC subtype II may also include fibroblast-like VSMCs (specific markers: *BGN*, *COL1A2*, *COL3A1*) that have been shown to be present in both diseased and normal aorta (Figure 3B, dot plot) (33, 34).

Taken together, these data indicate that CDKL1 is expressed in VSMCs in the media of the aorta and of vasa vasorum; however, it is not yet entirely clear whether CDKL1-positive cells — in particular those strongly expressing CDKL1 — likely represent specific VSMC subpopulations.

### Knockdown of Cdkl1 induces vascular malformation and aortic dilation in zebrafish.

**Figure 4:**
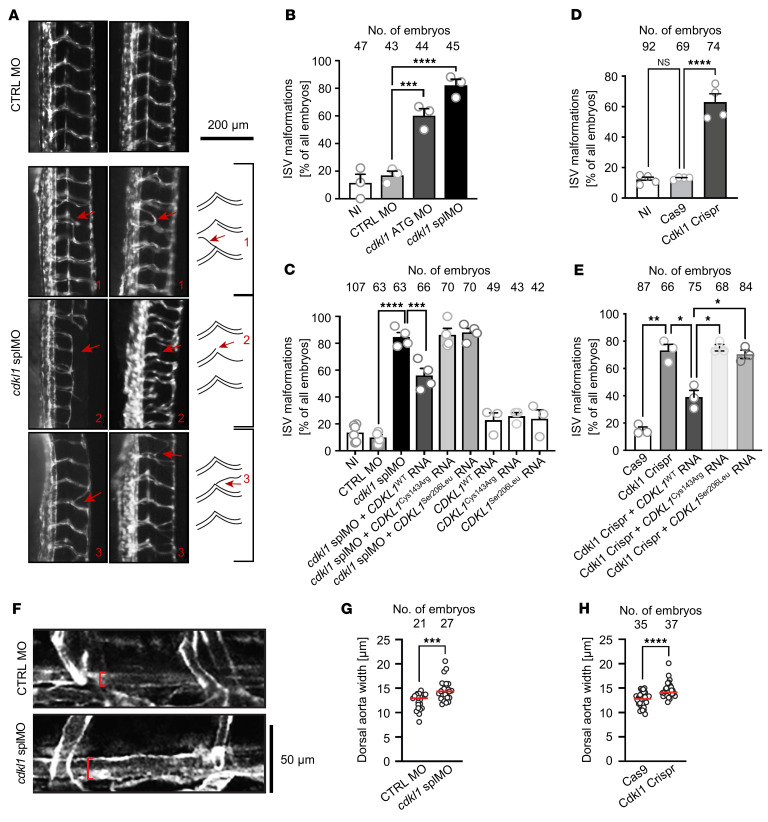
ISV malformation and aortic dilation in zebrafish *cdkl1* morphants and crispants. (**A**) ISV malformations in zebrafish embryos. Representative fluorescence images are shown for each phenotype; dorsal to the right. Cartoon illustrates aberrantly formed ISVs in 48-hpf fli-GFP embryos. (**B**) Quantification of ISV formation defects. (**C**) Coinjection of RNA encoding CDKL1^WT^ rescued ISV defects upon MO-induced Cdkl1 knockdown, while variants encoding CDKL1^Cys143Arg^ and CDKL1^Ser206Leu^ are unable to rescue ISV malformations. Overexpression of CDKL1^WT^ or its variants did not induce ISV defects. (**D**) Cdkl1 crispants (Cdkl1 Crispr) display ISV defects more often than do non-injected (NI) embryos or embryos injected with Cas9 only. Single ribonucleoprotein complexes including 2 guide RNAs (targeting Cdkl1 exons 6 and 7) and Cas9 were injected into the first cell of fertilized zebrafish eggs; as control, eggs were injected with Cas9 only. 1-way ANOVA with Šidák’s multiple-comparison tests were applied (**B**, **C**, and **D**). Bar graphs show means + SEM; *n* = 3 (**B**), *n* = 3–6 (**C**), and *n* = 4 (**D**) experiments. NS, *P* = 0.9806; ***P ≤ 0.001, ****P ≤ 0.0001. (**E**) Coinjection of RNA encoding CDKL1^WT^ rescued ISV defects upon CRISPR/Cas–mediated Cdkl1 knockdown, while neither mutant variant did so. *n* = 3 experiments. *P ≤ 0.05, **P = 0.0040, Brown-Forsythe and Welch’s ANOVA test with Dunnett’s T3 multiple-comparison test. (**F**) Cdkl1 morphants display aortic dilation. Fli-GFP zebrafish embryos were injected with control (CTRL) MO or Cdkl1 splMO. Representative image of the dorsal aorta in embryos 4 days post-fertilization. Red bracket indicates width of the aorta. (**G**) Quantification of aortic dilation. (**H**) Cdkl1 crispants display aortic dilation. Single ribonucleoprotein complexes including 2 guide RNAs (targeting Cdkl1 exons 6 and 7) and Cas9 were injected into the first cell of fertilized zebrafish eggs; as control, eggs were injected with Cas9 only. 2-tailed Mann-Whitney test was applied; *n* = 3 experiments; red lines show medians; ***P = 0.0003, ****P < 0.0001 (**G** and **H**). Circles in the graphs indicate individual experiments, and numbers of embryos analyzed are given (**B**–**E**, **G**, and **H**).

We next assessed whether CDKL1 is involved in vascular biology using a zebrafish model. All affected CDKL1 amino acids described in this study are conserved in zebrafish (Supplemental Figure 1). Whole-mount in situ hybridization during zebrafish development revealed *cdkl1* expression in ciliated tissues including the neural tube, the pronephros, and the olfactory pit (beginning at 13 somite stage [ss], 20 ss, and 24 hours post-fertilization [hpf], respectively) (Supplemental Figure 8A). We blocked Cdkl1 expression by injection of 2 different morpholino oligonucleotides (MOs) targeting either the start codon (ATG MO) or a splice site (splMO; splice blocking efficiency was verified by quantitative reverse transcriptase PCR; Supplemental Figure 8B) in zebrafish embryos and analyzed intersomitic vessels (ISVs). ISVs connect the dorsal aorta or posterior cardinal vein to the dorsal longitudinal anastomotic vessels and are considered a model system for the investigation of vascular development. Knockdown of Cdkl1 by either MO resulted in ISV malformations, shortening, and breakdown (Figure 4, A and B). This could be partially but significantly rescued by coinjection of RNA encoding human wild-type CDKL1 (CDKL1^WT^), thus confirming the specificity of the vascular defects and cross-species conservation of *cdkl1* (Figure 4C). In contrast, coinjection of RNA coding for CDKL1^Cys143Arg^ or CDKL1^Ser206Leu^ did not rescue the ISV phenotype (Figure 4C). Injection of any *CDKL1* RNA (encoding wild type, p.Cys143Arg, or p.Ser206Leu) alone did not produce significant vascular defects in zebrafish embryos (Figure 4C). However, long-term manifestations in adult zebrafish expressing CDKL1 missense variants cannot be excluded.

To model Cdkl1 loss of function and to verify a vascular phenotype in zebrafish, we applied transient CRISPR/Cas9–mediated gene editing: Cdkl1 crispants showed strong ISV malformation (Figure 4D and Supplemental Figure 8C). This could be significantly rescued by coinjection of RNA encoding human wild-type CDKL1 (CDKL1^WT^), but not by coinjection of mutant CDKL1^Cys143Arg^ or CDKL1^Ser206Leu^ (Figure 4E). Next, we analyzed dorsal aortic morphology in 4-day-old zebrafish upon both knockdown and loss of Cdkl1 expression. Blocking Cdkl1 expression by injection of a splice site MO (splMO) resulted in significant aortic dilation (Figure 4, F and G). The dorsal aorta was also significantly dilated upon CRISPR/Cas9–mediated knockout of Cdkl1 in zebrafish embryos compared with embryos injected with Cas9 only (Figure 4H). Summarized, data from the zebrafish model suggest that loss of functional CDKL1 leads to abnormal vascular formation and aortic dilation.

### CDKL1 variants show reduced kinase activities and affect substrate phosphorylation profiling.

**Figure 5:**
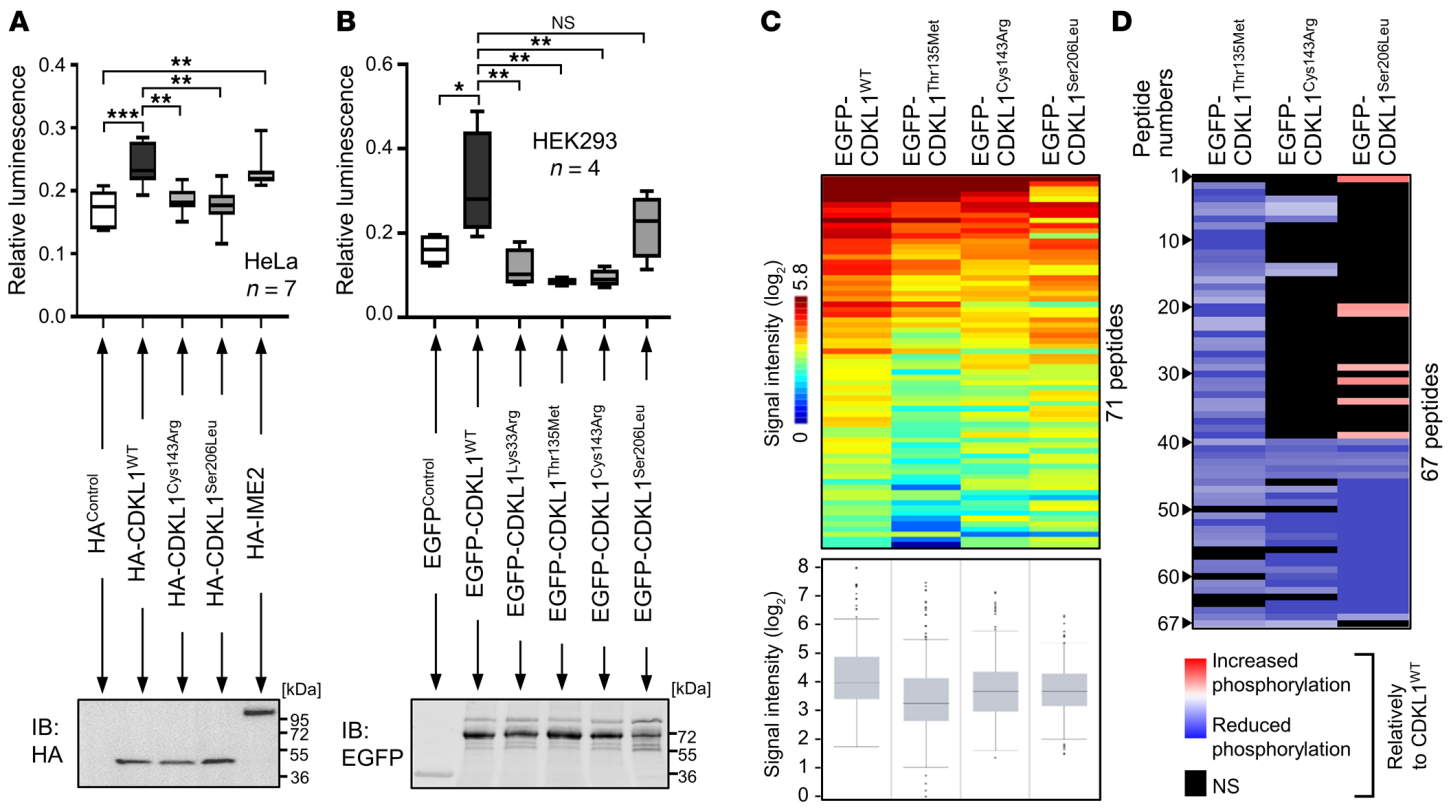
*CDKL1* variants interfere with kinase function. (**A** and **B**) In vitro kinase assays. HeLa (**A**) or HEK293T (**B**) cells were transiently transfected with *CDKL1* and *IME2* constructs or empty vectors (HA^control^ and EGFP^control^). Fusion proteins were immunoprecipitated and subjected to kinase assays using peptide substrate RPRSPGARR and the ADP-Glo kinase assay (Promega). Luminescence intensities of individual measuring points were normalized to the entire luminescence signal of an experiment and to the amount of immunoprecipitated protein (*n* = 7, **A**; *n* = 4, **B**). Medians (50th percentiles, lines in boxes) as well as 0th, 25th, 75th, and 100th percentiles are given in the box plots. Small aliquots were removed from precipitates and subjected to immunoblotting using anti-HA (**A**) and anti-GFP (**B**) antibodies; representative blots are shown. *P ≤ 0.05, **P ≤ 0.01, ***P ≤ 0.001, 1-way ANOVA with Dunnett’s post hoc multiple-comparison test. (**C**) CDKL1 serine/threonine phosphorylation profiling. EGFP-tagged CDKL1 variants were expressed in HEK293T cells, purified using GFP-Trap (ChromoTek), and applied to STK-PamChip arrays (PamGene). Seventy-one peptides passed quality control. The heatmap displays average log_2_-transformed signal intensities for indicated CDKL1 variants. The signals were sorted from high (red) to low (blue) intensity that corresponds to phosphorylation levels. To visualize overall sample variance and group differences, peptide phosphorylation and overall kinase activity are shown in box plot representation. (**D**) Peptide substrates with significantly changed phosphorylation. The heatmap shows significantly differentially phosphorylated peptides between samples treated with disease-associated CDKL1 variants versus CDKL1^WT^. The effects of CDKL1 variants are log ratios [log_2_(disease-associated CDKL1 variant)/log_2_(CDKL1^WT^)]. Blue and red indicate reduced and increased phosphorylation, respectively. Peptides that did not pass the significance threshold (*P* < 0.05, disease-associated CDKL1 variant vs. CDKL1^WT^) are black. Peptide numbers correspond to those in Supplemental Table 2, where details on the substrates are also described.

Two of the identified CDKL1 changes affect the magnesium binding loop (Supplemental Figure 1); therefore, we investigated whether the identified variants interfere with CDKL1 kinase activity. We expressed CDKL1 wild-type (CDKL1^WT^), CDKL1^Cys143Arg^, CDKL1^Ser206Leu^, and CDKL1^Thr135Met^ in HeLa and HEK293T cells and performed in vitro kinase assays. Since no physiological substrate of CDKL1 is known, we used a suboptimal substrate (peptide sequence RPRSPGARR) that has been determined for the *Saccharomyces* *cerevisiae* kinases IME2 and CDC28 and applied previously to measure CDKL1 kinase activity (8, 35). *S*. *cerevisiae* IME2 and the kinase-dead variant CDKL1^Lys33Arg^ were used as positive and negative controls, respectively (8). Compared with empty vector controls and CDKL1^Lys33Arg^, increased kinase activity was observed both for CDKL1^WT^ and IME2 (Figure 5, A and B). We detected significantly reduced kinase activity for CDKL1^Cys143Arg^ (Figure 5, A and B), CDKL1^Ser206Leu^ (Figure 5A), and CDKL1^Thr135Met^ (Figure 5B). These data demonstrate that p.Cys143Arg, p.Ser206Leu, and p.Thr135Met affect CDKL1 kinase function in vitro.

To verify that CDKL1^Cys143Arg^, CDKL1^Ser206Leu^, and CDKL1^Thr135Met^ do impair CDKL1 kinase function, we used the microarray-based PamGene technology (https://pamgene.com/technology/). Our primary aim was not to identify CDKL1 substrates, because this in vitro method does not reveal what takes place physiologically. Assay functionality experiments are shown in Supplemental Figure 9, A and B. We applied recombinant CDKL1^WT^, CDKL1^Thr135Met^, CDKL1^Cys143Arg^, or CDKL1^Ser206Leu^ to kinase reactions on peptide microarrays and quantified the phosphorylation of 144 serine/threonine substrate peptides. We observed differences in peptide phosphorylation patterns and, therefore, CDKL1 serine/threonine kinase function between CDKL1^WT^ and disease-associated CDKL1 variants (Supplemental Figure 9C). Combined replicate results show generally lower signal intensities of phosphorylated peptides for disease-associated CDKL1, as confirmed by box plot analysis (Figure 5C). Phosphorylation of 60 peptides was significantly decreased in CDKL1^Thr135Met^-treated samples compared with CDKL1^WT^, of 30 in CDKL1^Cys143Arg^-treated samples, and of 27 in CDKL1^Ser206Leu^-treated samples (Figure 5D). In addition, application of CDKL1^Ser206Leu^ resulted in increased phosphorylation of 7 peptides (Figure 5D). Phosphorylation of 20 peptide substrates was significantly reduced for all 3 disease-associated CDKL1 variants compared with CDKL1^WT^ (Figure 5D). Information on peptide sequences is given in Supplemental Table 2. These data confirm that CDKL1^Cys143Arg^, CDKL1^Ser206Leu^, and CDKL1^Thr135Met^ interfere with CDKL1 kinase function.

### CDKL1 variants differ in protein complexing properties.

**Figure 6:**
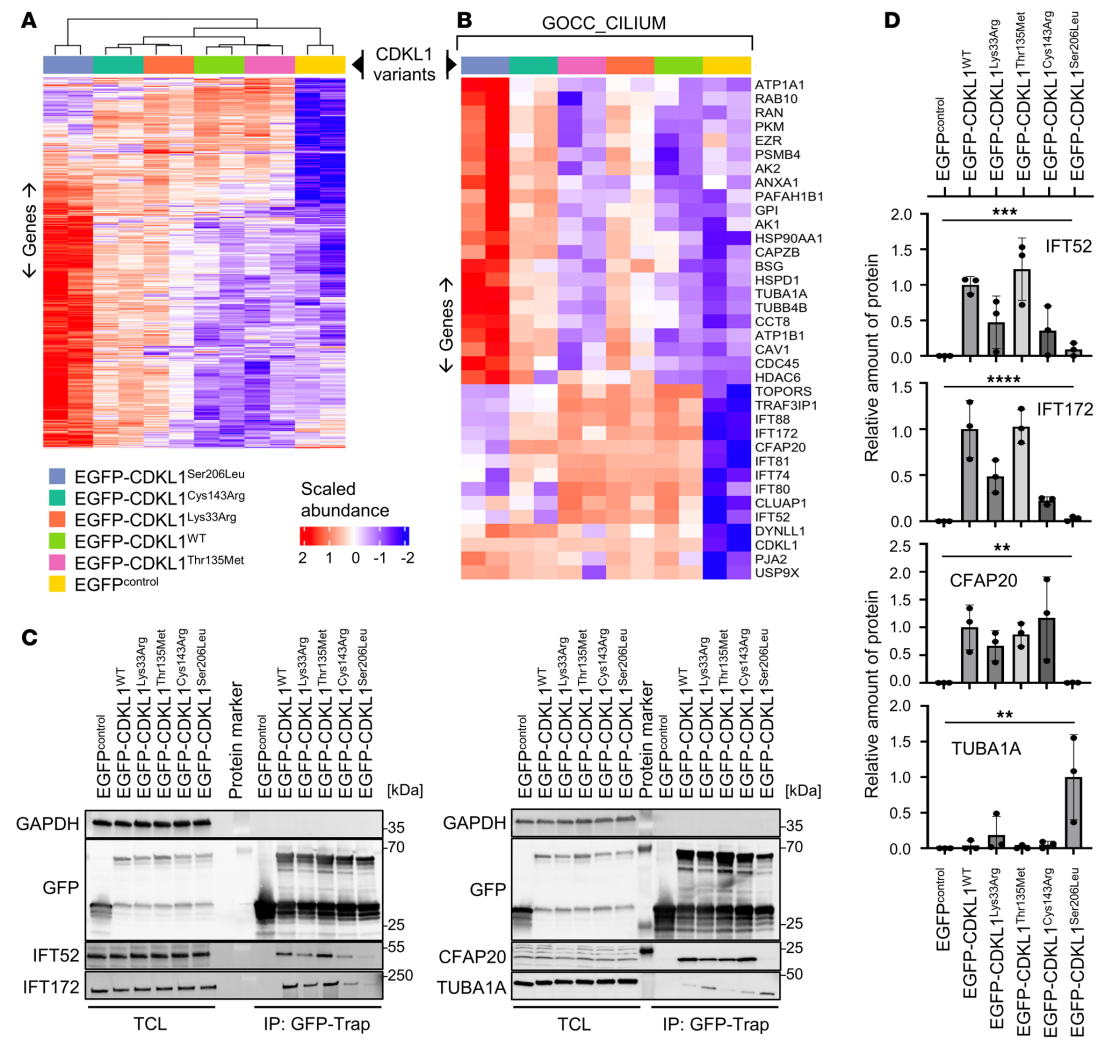
Disease-associated CDKL1 variants show different protein-protein interaction profiles. EGFP-tagged CDKL1 protein variants were expressed in HEK293T cells, purified by GFP-Trap, and subjected to liquid chromatography–tandem mass spectrometry analysis. Experimental duplicates were performed for all conditions. Protein abundances were normalized based on GFP protein peptide abundances. (**A** and **B**) Heatmaps: Pearson correlation–based hierarchical clustering of EGFP-vector, CDKL1^WT^, CDKL1^Lys33Arg^, CDKL1^Thr135Met^, CDKL1^Cys143Arg^, and CDKL1^Ser206Leu^ samples with average linkage, based on 626 ANOVA-significant proteins (**A**) and on 36 cilium proteins, assigned to the GOCC gene set Cilium (**B**) between all analyzed phenotypes (adjusted *P* value < 0.05). Normalized protein abundances were scaled before clustering for visual purposes. Relative protein abundance is coded by colors from red (high abundance) to blue (low abundance). (**C** and **D**) Validation of differentially affected protein-protein interactions. Total cell lysates (TCL) of HEK293T cells expressing EGFP-tagged CDKL1 protein variants or EGFP (control) were subjected to immunoprecipitation (IP) using GFP-Trap beads. CDKL1 expression and precipitation efficiencies were determined by anti-GFP immunoblotting. Coprecipitation and input levels of endogenous IFT52, IFT172, CFAP20, and TUBA1A were assessed by immunoblotting using specific primary antibodies; GAPDH was used as loading control (**C**). The graph shows mean relative amounts of coprecipitated IFT52, IFT172, CFAP20, and TUBA1A normalized to precipitated GFP (**D**). The mean of experiments for CDKL1^WT^ (IFT52, IFT172, CFAP20) and CDKL1^Ser206Leu^ (TUBA1A) was set to 1. **P ≤ 0.01, ***P ≤ 0.001, ****P ≤ 0.0001, 1-way ANOVA. *n* = 3 experiments.

To assess consequences for protein binding properties, we affinity-purified GFP-tagged CDKL1 variants from HEK293T lysates and analyzed precipitates by differential quantitative proteomics (tandem mass spectrometry). In total, we quantified 1,304 proteins; of these, 626 proteins were identified as ANOVA significant (*q* value < 0.05) between all compared variants (Figure 6A and Supplemental Table 3). CDKL1^Thr135Met^ showed a relatively small effect on abundances of coprecipitated proteins compared with CDKL1^WT^, with no *q* value–significant proteins being present. In contrast, protein abundances were strongly affected for CDKL1^Ser206Leu^ and CDKL1^Cys143Arg^ (log_2_ difference > 0.58 or < 0.58, at least 1.5 times more or less abundant than in CDKL1^WT^) considering *P* value–significant proteins (Figure 6A, Supplemental Figure 10, and Supplemental Table 3). Moreover, protein abundances for CDKL1^Ser206Leu^ strongly differed from those for CDKL1^WT^ samples and EGFP controls, as suggested by a high number of *q* value–significant proteins. This may indicate loss of physiological protein-protein interactions and extensive unspecific binding for this variant (Figure 6A, Supplemental Figure 10, and Supplemental Table 3). Finally, protein abundances in kinase-dead CDKL1^Lys33Arg^ samples were similar to those in CDKL1^Cys143Arg^ samples, consistent with impaired kinase activity for both variants (Figure 6A, Supplemental Figure 10, and Supplemental Table 3). Taken together, these data suggest variant-specific consequences for CDKL1 binding properties in addition to mutation effect overlaps. Notably, 36 of 626 (5.75%) significantly differentially abundant proteins between all compared CDKL1 variants were found in the Gene Ontology Cellular Component (GOCC) gene/protein set Cilium (GO:0005929) (Figure 6B). In particular, proteins involved in intraflagellar transport (IFT) (e.g., IFT52, IFT172, etc.) showed major differences between CDKL1 variants (Figure 6B), indicating that disease-associated CDKL1 variants may affect intraflagellar transport to cilia. We validated CDKL1 variant–specific effects on protein-protein interaction with IFT52, IFT172, CFAP20, and TUBA1A by using coimmunoprecipitations and statistical analyses (Figure 6, C and D).

### CDKL1 variants interfere with cilia formation, cilia length, and ciliary localization of CDKL1.

**Figure 7:**
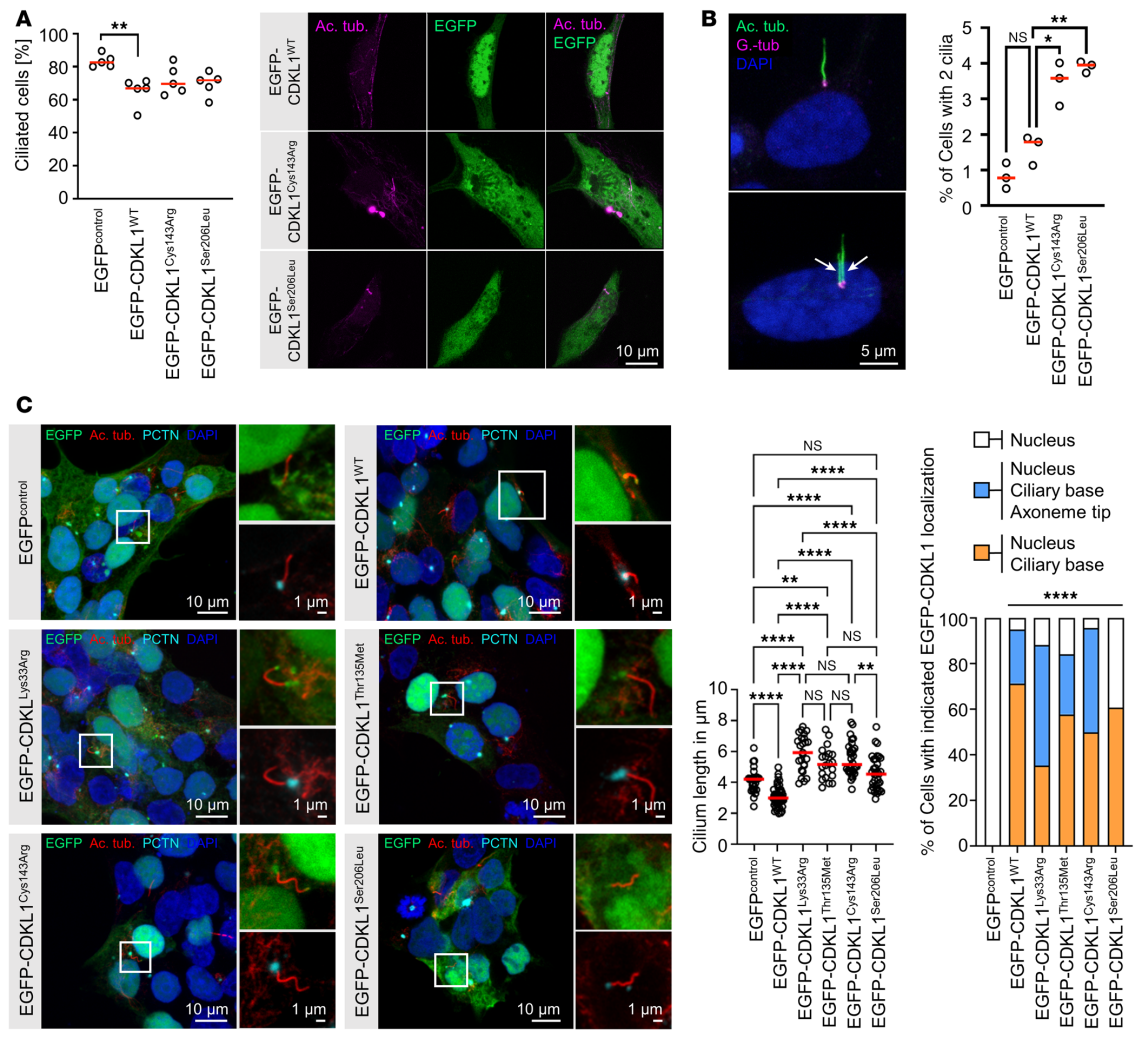
Consequences of CDKL1 variants for ciliary biology. (**A**) Expression of CDKL1 variants affects primary cilia formation. EGFP-CDKL1 variants were transiently expressed in RPE-1 cells (green), serum-starved to induce ciliation, and stained for the axoneme of primary cilia with anti–acetylated tubulin (Ac. tub., magenta). Scale bar: 10 μm. *n* = 5 experiments with more than 100 cells per condition and experiment counted. **P = 0.0083, Kruskal-Wallis test with Dunn’s multiple-comparison post-test. (**B**) Expression of disease-associated CDKL1 variants increases number of bi-ciliated RPE-1 cells. EGFP-CDKL1 variants were transiently expressed in RPE-1 cells (green) and stained for the axoneme with anti–acetylated tubulin (Ac. tub., green) and for the basal body with anti–γ-tubulin (G.-tub., magenta) antibodies. Scale bar: 5 μm. *n* = 3 experiments with 211–441 ciliated cells counted. *P = 0.0309, **P = 0.0070, Brown-Forsythe and Welch’s ANOVA test with Dunnett’s T3 multiple-comparison test. (**C**) CDKL1 variants localize at the basal body (and tip) of the primary cilium and affect cilia length in HEK293T cells. EGFP-CDKL1 variants were transiently expressed in HEK293T cells (green). Cells were stained for the axoneme of primary cilia with anti–acetylated tubulin (red) and for the basal body with anti-pericentrin (cyan) antibodies. Nuclei were stained with DAPI (blue). Shown are representative images of CDKL1-expressing cells with primary cilia; further images are given in Supplemental Figure 11. Scale bars: 10 μm, and 1 μm in close-up images. Quantification of cilium length: *n* = 3 experiments with ≥20 cilia per condition measured; median (red bars) and individual experiments (circles) are indicated. **P < 0.01, ****P < 0.0001, Brown-Forsythe and Bartlett’s ANOVA test with Tukey’s multiple-comparison test. Quantification of CDKL1 localization: Stacked bar graphs summarize 3 experiments with ≥17 cilia per condition analyzed. ****P < 0.0001, Fisher’s exact test. All images (**A**–**C**) were acquired with a confocal microscope.

*Caenorhabditiselegans* Cdkl1 localizes to the transition zone above the basal body and controls cilia length (8, 36, 37). To test whether disease-associated variants affect cilia formation, cilia length, or ciliary localization of CDKL1, we expressed EGFP-tagged CDKL1 variants in RPE-1 and HEK293T cells and costained acetylated tubulin (as a marker for the axoneme) and γ-tubulin or pericentrin (as markers for the basal body). Overexpression of any variant (WT, p.Cys143Arg, p.Ser206Leu) in RPE-1 cells reduced the number of ciliated cells to a similar level, in comparison with vector controls (Figure 7A). Gene dosage appeared to matter, as RPE-1 cells strongly overexpressing EGFP-CDKL1^WT^ no longer formed any cilium, whereas cells strongly overexpressing EGFP-CDKL1^Cys143Arg^ or EGFP-CDKL1^Ser206Leu^ still did (Figure 7A, microscopy images). Moreover, RPE-1 cells transfected with CDKL1 patient variants extended 2 cilia significantly more often than cells expressing wild-type CDKL1 (Figure 7B). In HEK293T cells, expression of CDKL1^WT^ shortened ciliary length in comparison with EGFP^control^ cells, consistent with an inhibitory effect on ciliation seen in RPE-1 cells, and expression of CDKL1^Cys143Arg^, CDKL1^Thr135Met^, and CDKL1^Lys33Arg^ resulted in elongated cilia (Figure 7C and Supplemental Figure 11). Cilia length in cells expressing CDKL1^Ser206Leu^ was similar to that in EGFP^control^ cells (Figure 7C and Supplemental Figure 11). Statistical evaluation confirmed these observations (Figure 7C). To identify whether this may be a direct effect of CDKL1 on cilia, we assessed the subcellular localization more closely. All CDKL1 variants showed a prominent nuclear localization (Figure 7C and Supplemental Figure 11). In addition, CDKL1^WT^ localizes to the tip of the axoneme in 23.8% of cells and to the ciliary base in 95.2% of cells (i.e., 71.4% base + 23.8% base and tip); we observed a similar distribution for CDKL1^Thr135Met^ (Figure 7C and Supplemental Figure 11). The localization at the tip of the axoneme was increased for CDKL1^Cys143Arg^ (45.8%) and CDKL1^Lys33Arg^ (52.9%) (Figure 7C and Supplemental Figure 11). CDKL1^Ser206Leu^ did not localize to the tip of the axoneme and could be observed at the base of the cilium only in 60.7% of cells (Figure 7C and Supplemental Figure 11). These data demonstrate that human CDKL1 controls cilia formation and that disease-associated CDKL1 variants display altered functionality toward cilia.

### CDKL1 variants increase p38 (MAPK14) signaling in HEK293T cells, and knockdown of Cdkl1 induces Vegf signaling in zebrafish.

**Figure 8:**
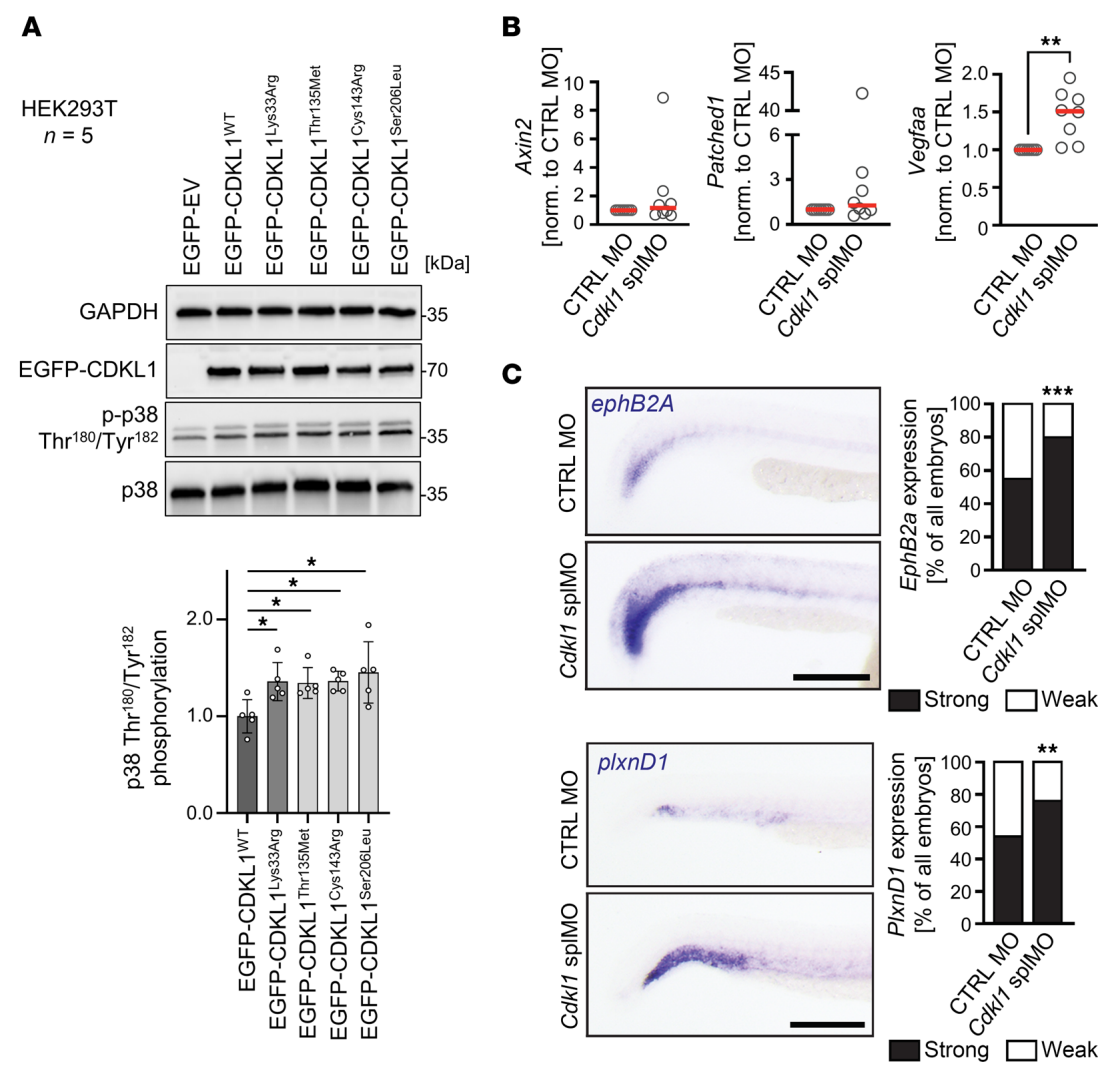
CDKL1 function in cell signaling. (**A**) CDKL1 variants increase signaling via p38 (MAPK8). Total lysates of HEK293T cells transiently expressing CDKL1^WT^, CDKL1^Thr135Met^, CDKL1^Cys143Arg^, or CDKL1^Ser206Leu^ were cultured under basal conditions (DMEM containing 10% serum), lysed, and subjected to immunoblotting as indicated. Cells expressing kinase-dead CDKL1^Lys33Arg^ and cells transfected with empty vector (EV) were used as controls. GAPDH was used as loading control. Representative immunoblots from 5 independent experiments (*n* = 5) are given. Graph shows means (± SD) of the relative phosphorylation of p38 (at Thr^180^ and Tyr^182^) normalized to amounts of total p38 as well as to GAPDH. Phosphorylation levels in cells expressing CDKL1 mutants are relative to those in CDKL1^WT^ cells. One-way ANOVA with Tukey’s multiple-comparison test was used. *P ≤ 0.05, **P ≤ 0.01. (**B**) Knockdown of *Cdkl1* induces Vegfaa expression. Quantitative PCR analysis was performed on total RNA of 24 hpf zebrafish embryos. Graphs show amounts of *axin2*, *patched1*, and *vegfaa* transcripts in *Cdkl1* splMO relative to CTRL MO–treated embryos. *n* = 8 experiments; mean values (red bars) and individual experiments (circles) are indicated. **P = 0.07, 2-tailed Wilcoxon’s test. (**C**) Expression of the arterial marker ephrin B2a (*ephB2a*) and of plexin D1 (*plxnD1*) is stronger in Cdkl1 morphants than in control-injected embryos. Spatial expression was determined by in situ hybridization. Stacked bar graphs summarize 3 experiments with 82 (CTRL MO) and 85 embryos (Cdkl1 splMO) for *ephB2a* and 3 experiments with 63 (CTRL MO) and 42 embryos (Cdkl1 splMO) for *plxnD1*. **P = 0.0018, ***P = 0.0003, 2-sided Fisher’s exact test. All experiments were done at 24 hpf. Scale bars: 200 μm.

To track down CDKL1-induced alterations in specific cell signaling pathways, we carried out extensive immunoblot analyses in HEK293T cells cultured under basal conditions. We could not detect altered expression or activation of proteins associated with cilia biology (NOTCH-, WNT-, and SHH-dependent signaling routes) (Supplemental Figure 12) or involved in cell cycle regulation (cyclins, CDKs, and p21^Waf1/Cip1^) (Supplemental Figure 13). For background information regarding these analyses, see Supplemental Results under the subsection Analysis of candidate CDKL1-dependent signaling pathways. TAAD is closely associated with dysregulation of TGFB signaling (1, 2), and TGFB signaling plays multiple roles in cilia (38). We could not identify an involvement of CDKL1 in canonical TGFB signaling via SMAD2/3 (Supplemental Figure 14A) and non-canonical TGFB signaling branches via MEK-ERK and AKT (Supplemental Figure 14, B and D). TGFB can also rapidly activate the so-called stress-activated protein kinases (SAPKs) p38 (MAPK14) and JNK (MAPK8) in a SMAD-independent (i.e., non-canonical) manner, including in cilia (39, 40). CDKL1 p.Lys33Arg, p.Cys143Arg, p.Thr135Met, and p.Ser206Leu significantly increased p38 phosphorylation (Figure 8A). Moreover, expression of disease-associated CDKL1 variants slightly induced phosphorylation of JNK compared with CDKL1^WT^ (Supplemental Figure 14C). These data suggest that expression of CDKL1 variants results in dysregulation of p38, which is involved in a wide variety of cellular processes, such as proliferation, differentiation, development, survival, apoptosis, senescence, and cytokine production. Next, we tested canonical Wnt signaling (via the downstream effector Axin2), Hedgehog signaling (via the downstream effector Patched1), and Vegf signaling in zebrafish. Only Vegfaa, which is orthologous to human VEGFA, showed significant upregulation upon knockdown of Cdkl1 (Figure 8B). This is consistent with previous reports suggesting that elevated Vegf signaling is associated with ISV defects and induction of plexin D1 expression (41, 42). In line with this, Cdkl1 inhibition resulted in elevated expression of the arterial marker proteins ephB2A and plexin D1 (Figure 8C), which have previously been associated with expansion of arterial cells (43) and aberrant ISV formation, respectively (44).

## Discussion

### Clinical aspects.

Recurrent vascular manifestations in our series of individuals with heterozygous *CDKL1* include aneurysms and dissections of the thoracic/abdominal aorta and of the carotid arteries (Table 1). In 5 of the 6 patients, craniofacial and/or skeletal manifestations were diagnosed (Table 1). Other clinical features in the individuals described in this study, such as hernias, dural ectasia, and myopia, have been reported in hereditary aortopathies, e.g., Marfan syndrome, Loeys-Dietz syndrome, and Ehlers-Danlos syndrome (2, 4). Patients with *CDKL1* variants show overlaps and differences in their clinical manifestations; this may reflect similar and variant-specific cell physiological effects of *CDKL1* variants as detected in our functional tests. In line with the clinical presentation of the patients in this study, several TAAD disease genes (e.g., *SMAD3* and *TGFBR1*) are associated with a broad phenotypic spectrum including aneurysms/dissections of other arteries in addition to the aorta (45). Taken together, the collected clinical data may suggest that *CDKL1*-associated vasculopathy might be characterized by an involvement of large to medium-sized arteries in addition to the clinical manifestations known for hereditary aortopathies (2, 4).

Recently, it was reported that de novo variants in *CDKL1* and *CDKL2* are associated with neurodevelopmental symptoms (46). Regarding the 2 identified *CDKL1* variants, dominant-negative (antimorphic) consequences have been suggested (46). In contrast, our data indicate amorphic/hypomorphic, neomorphic, and hypermorphic consequences of CDKL1 variants identified in our patients (for details, see Supplemental Data: Discussion). Notably, 2 of the patients with *CDKL2* variants show subdural hemorrhage and connective tissue defects (46), suggesting pleiotropic manifestations of *CDKL2* variants. In general, ciliary dysfunction has been associated with a remarkable variability of phenotypic presentation with multiorgan involvement and allelism (47, 48). Clinical presentation ranges from single-organ impairment to complex systemic disorders including renal, endocrine, cardiovascular, skeletal, respiratory, genital, pancreatic, hepatic, retinal, neuronal, and other anomalies (49, 50). In conclusion, *CDKL1* variants may exert allelic and pleiotropic consequences affecting various tissues and organs.

### Genetic perspectives and architecture.

Hereditary aortopathies are characterized by reduced penetrance, highly variable expressivity, and late onset; moreover, the presence of genetic modifiers and reduced-penetrance alleles has been proposed (2, 4, 51, 52). This may also explain the negative family history in family 3 (Table 1). Moreover, a complex genetic architecture (di-/oligo-/polygenic inheritance) with rare variants having large effects and common alleles having low to moderate effect sizes may account for a considerable number of aortic disease (3, 6, 52). Here, we describe variable expressivity (e.g., age of disease onset, affected arteries; Table 1, families 1 and 2) and concomitant variants (patient P6). Therefore, we suggest that adverse functional *CDKL1* variants may represent dynamic disease alleles for a CTD with potentially marked effect sizes depending on the respective genetic background. Taken together, we support the notion that hereditary aortopathies and arterial aneurysms/dissections show a complex genetic architecture with penetrant mutations and variants with small to moderate effect sizes (3, 52).

### Zebrafish vascular defects as indicator of human TAAD-associated genes.

It has been reported that zebrafish *cdkl1* morphants showed eye anomalies, small brains, and curved bodies, and coinjection of wild-type *cdkl1* mRNA partially rescued these phenotypes, so that *zcdkl1* was regarded as an important developmental gene (53). In our hands, however, no gross morphological changes in the embryos devoid of functional Cdkl1 could be observed except for vascular defects. Indeed, we demonstrated that knockdown and transient knockout of *cdkl1* induces aortic dilation and ISV malformation in zebrafish. Several groups have used the zebrafish model to investigate the effects of TAAD-associated genes on the vasculature: Global *Secisbp2* (i.e., zebrafish ortholog of the TAAD disease gene *SECISBP2*) deficiency resulted in dilation of the ventral aorta in zebrafish larvae (54). Injection of TAAD-associated *SMAD3* variant mRNA resulted in enlargement of the dorsal aorta in zebrafish embryos (55). Knockdown of the *mat2aa* gene (i.e., zebrafish ortholog of the TAAD-associated gene *MAT2A*) resulted in developmental defects including malformation of the aortic arches (56). Furthermore, it has been demonstrated that knockout of the Marfan syndrome– and Ehlers-Danlos syndrome–related genes *fbn1*, *col1a2*, *col5a1*, and *col5a2* resulted in various cardiovascular and skeletal defects such as reduced diameter of the aorta and ISVs, inappropriate ISV branching, and scoliosis in zebrafish (57).

### Molecular pathology.

Recently, Canning et al. revealed a role for *C*. *elegans* *cdkl1* in the length regulation of primary cilia, organelles that perform essential roles in human sensory physiology, cell signaling, and development (8, 37); this function was lost in a kinase-dead mutant and by introduction of disease-linked mutations (8). Consequently, a possible role for mammalian CDKL proteins in human physiology and disease has been suggested (8, 37). IFT is required for the assembly and maintenance of cilia, as well as for cilium-dependent signaling; IFT protein complexes function as adaptors facilitating cargo transport between the base and tip of the cilium (58, 59). The basal body anchors and positions the cilium within the cell (58, 59). *C*. *elegans* *cdkl1* is an IFT-associated cargo protein that relies on IFT for its transport to cilia (37). Given the observation that CDKL1 variants interacting with IFT proteins (i.e., CDKL1^WT^, CDKL1^Thr135Met^, CDKL1^Cys143Arg^, and CDKL1^Lys33Arg^) but not IFT binding–deficient CDKL1^Ser206Leu^ localize at the axoneme (Figure 7C), we speculate that impaired binding to the IFT system may result in CDKL1 mislocalization. Consequently, we assume that altered subcellular distribution due to aberrant protein interactions may represent the basic molecular defect for CDKL1^Ser206Leu^, whereas impaired kinase function rather than mislocalization is the primary molecular defect for CDKL1^Thr135Met^, CDKL1^Cys143Arg^, and CDKL1^Lys33Arg^.

Primary cilia are fundamentally important for the structure and the function of VSMCs within the arteries (60, 61). Primary cilia of VSMCs are preferentially oriented, possess proteins critical for cell-ECM interactions, and respond to ECM proteins and mechanical stimulations (62, 63). Thus, the primary cilia of VSMCs can act as mechanochemical sensors (i.e., to sense the surrounding mechanical and chemical stimuli), thereby affecting VSMC function and phenotype (60, 62–69). Since VSMCs and the ECM are essential for regulating vascular tissue homeostasis, differentiation, and wound repair, cilium-mediated functions may be pivotal for maintaining vascular functional integrity (62, 69). More detailed background information is given in the Supplemental Data: Discussion. Based on this knowledge, it was proposed that proteins involved in mechanosensing biology are associated with aortic disease (70). Indeed, ciliary dysfunction can induce aneurysm formation and progression (60, 71–74), and several TAAD disease genes are involved in mechanosensing and the contractile response of cells (3, 51). We detected CDKL1-positive VSMCs in the aortic wall, and we showed that disease-associated, functional CDKL1 variants interfere with cilia formation and length, affect CDKL1 binding with proteins particularly involved in ciliary transport, and increase SAPK (p38) activation. p38 is central for the cellular response to virtually all types of stress, including mechanical forces perceived by cilia (60, 75). We speculate that increased activation of SAPK may result from stress induced by aberrant environmental sensing due to defective sensory cilia in VSMCs. Notably, increased p38 signaling upon cellular stress has been previously associated with different etiologies of cardiovascular disease (76). Upon knockdown of Cdkl1 in zebrafish, we detected increased Vegfaa, which is orthologous to human VEGFA. VEGF has essential roles in both vasculogenesis and angiogenesis, and it has been demonstrated that VEGF expression is stimulated by TGFB and positively regulated by p38 in several cell types (77–80). Moreover, increased VEGF has been associated with abdominal aneurysm in patients (81), and inhibition of VEGFA suppressed aneurysm formation in a mouse model (82). In conclusion, our findings support the idea that pathogenic CDKL1 variants interfere with cilia formation/homeostasis in aortic VSMCs, which causes aberrant environmental sensing by primary cilia and altered cilia-dependent signaling; this molecular dysfunction underlies a CTD associated with aortic/arterial aneurysm and dissection. The presented data, therefore, may expand our knowledge of the molecular pathophysiology of this group of disorders.

### Limitations.

The pedigrees of the families described in this study are small for a complex inherited disorder known for its heterogeneous genetic and environmental factors. Moreover, phenotypes and CDKL1 genotypes of several relatives are not available; thus cosegregation analysis is incomplete. Exome sequencing and targeted next-generation sequencing do not capture structural variants, copy number variations, or non-coding/intronic variants. Thus, it cannot be ruled out that such variants within known disease genes cause the clinical manifestation in our patients. The association, discussed above, of increased SAPK activation in HEK293T cells and enhanced Vegfaa abundance in zebrafish is a speculative attempt at explanation that requires validation. Alternatively, defective ciliary biology first may lead to altered VEGF abundance, resulting in SAPK activation.

## Methods

### Sex as a biological variable.

Sex was not considered as a biological variable.

### Analytical and descriptive procedures.

All experimental work, i.e., all analytical and descriptive analyses, is reported in Supplemental Methods.

### Statistics.

Methods of statistical analysis are given in Supplemental Methods. A *P* value less than 0.05 was considered significant. Types of statistical analyses and — where applicable — specific *P* values are specified in the respective figure legends.

### Study approval.

This study was performed in line with the principles of the Declaration of Helsinki. All analyses were performed clinically as required and approved by national legal regulation (German Genetic Diagnosis Act) and the local Institutional Review Board of the Hamburg Medical Association (votes PV3759, PV7038), Hamburg, Germany. Study participants gave their written informed consent to genetic analyses, clinical data and specimen collection, as well as pseudonymized publication of study results.

### Data availability.

All supporting data including materials and methods are available within the article and supplemental material. Values for all data points in graphs and behind reported means are provided in the Supporting Data Values file. Due to legal and ethical considerations, human sequencing data beyond those published as part of this project will not be made publicly available. Proteomics data have been deposited to the ProteomeXchange Consortium via the PRIDE partner repository with the dataset identifier PXD048044.

## Author contributions

GR conceptualized, supervised, and administered the study. MP, TS, TJD, and GR acquired funding. TN, MP, MK, HV, MA, TJD, and GR wrote (parts of) the original draft of the manuscript. MS, CK, TJD, and GR reviewed and edited the manuscript. TN, SR, MDB, BS, AB, VR, SD, and K Hoffer conducted experiments. TN, MP, CS, K Händler, BS, TM, MK, HV, MA, SK, and GR acquired, analyzed, and curated data. HS, JO, TSM, YVK, EG, MR, TS, and TJD identified the patients, performed clinical assessment, and provided clinical samples.

TN did most of the experimental work and generated most of the data; therefore, she merited the first place in the order of the first authors. The contribution of MP as a principal investigator was critical for the article, as she enabled zebrafish experiments and immunocytochemistry in RPE-1 cells. It was felt that MP merited the second place in the shared first authorship. Both first authors agreed to this order of first authorship.

## Funding support

- Deutsche Forschungsgemeinschaft (DFG) RO3660/3-1 and RO3660/7-1 to GR.
- DFG SA4284/4-1 to TS.
- DFG PH144/4-1, PH144/4-3, and 467868420 (funding of Stellaris 5 confocal microscope) to MP.
- Clinician Scientist Programme of the German Centre for Cardiovascular Research (FKZ 81X3710109 to TJD).
- DFG (no. 247354600 to the Core Facility Mass Spectrometry and Proteomics).

## Supplementary Material

Supplemental data

Unedited blot and gel images

Supplemental table 3

Supporting data values

## Figures and Tables

**Table 1:**
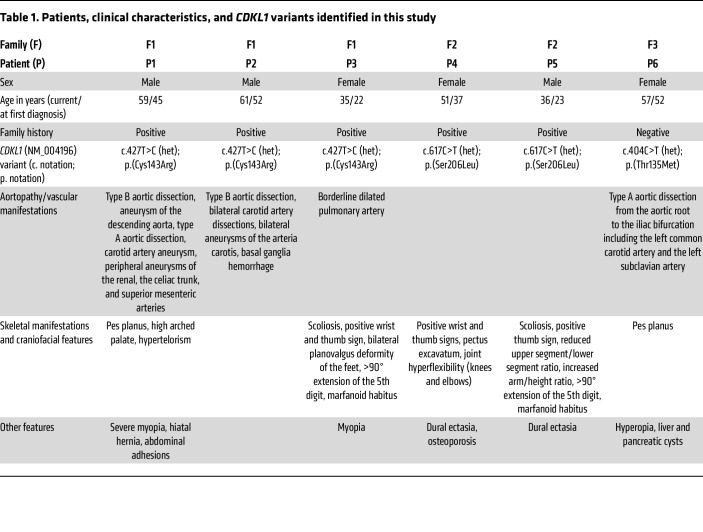
Patients, clinical characteristics, and *CDKL1* variants identified in this study

**Table 2:**
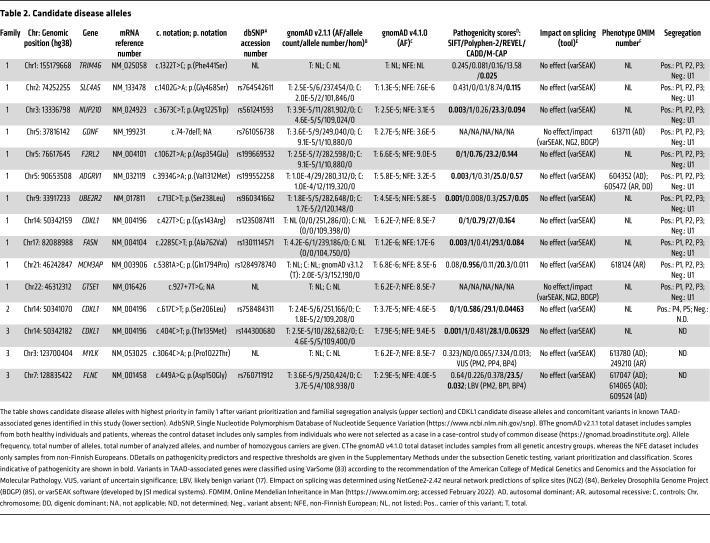
Candidate disease alleles

## Version 1 10/07/2025

In-Press Preview

## Version 2 12/01/2025

Electronic publication
