# Supplementary material for: CDKL1 variants affecting ciliary formation predispose to thoracic aortic aneurysm and dissection
Source: J Clin Invest. 2025 Oct 7;135(23):e186287. doi: 10.1172/JCI186287 (PMC12646653; doi:10.1172/JCI186287)
Supplement: Supplemental data [file jci-135-186287-s231.pdf]

SUPPLEMENTAL MATERIAL

CDKL1 variants affecting ciliary formation predispose to thoracic aortic aneurysm and dissection

Theresa Nauth[1], Melanie Philipp[2], Sina Renner[1], Martin D. Burkhalter[2], Helke Schüler[3], Ceren Saygi[4], Kristian Händler[5], Bente Siebels[6], Alice Busch[1], Thomas Mair[6], Verena Rickassel[1], Sophia Deden[1], Konstantin Hoffer[7,8], Jakob Olfe[9], Thomas S. Mir[9], Yskert von Kodolitsch[3,16], Evaldas Girdauskas[10,17], Meike Rybczynski[3], Malte Kriegs[7,8], Hannah Voß[6,18], Thomas Sauvigny[11], Malte Spielmann[5,12,13], Malik Alawi[4], Susanne Krasemann[14,15], Christian Kubisch[1], Till J. Demal[10], Georg Rosenberger[1,*]

[1]Institute of Human Genetics, University Medical Center Hamburg-Eppendorf, Hamburg, Germany

[2]Department of Experimental and Clinical Pharmacology and Pharmacogenomics, Section of Pharmacogenomics, Eberhard-Karls-University Tübingen, Tübingen, Germany

[3]Department of Cardiology, University Heart & Vascular Center Hamburg, Hamburg, Germany

[4]Bioinformatics Core, University Medical Center Hamburg-Eppendorf, Hamburg, Germany

[5]Institute of Human Genetics, Universitätsklinikum Schleswig-Holstein (UKSH), University of Lübeck and University of Kiel, Lübeck, Germany

[6]Section Mass Spectrometry and Proteomics, Center for Diagnostics, University Medical Center Hamburg-Eppendorf, Hamburg, Germany

[7]University Cancer Center Hamburg (UCCH) Kinomics Core Facility, Hubertus Wald Tumorzentrum - UCCH, University Medical Center Hamburg-Eppendorf, Hamburg, Germany

[8]Department of Radiotherapy and Radiation Oncology, Hubertus Wald Tumorzentrum - University Cancer Center Hamburg (UCCH), University Medical Center Hamburg-Eppendorf, Hamburg, Germany

[9]Clinic for Children's Heart Medicine and Adult Congenital Heart Disease, University Heart & Vascular Center Hamburg, Hamburg, Germany

[10]Department of Cardiovascular Surgery, University Heart & Vascular Center Hamburg, Hamburg, Germany

[11]Department of Neurosurgery, University Medical Center Hamburg-Eppendorf, Hamburg, Germany

[12]Human Molecular Genetics Group, Max Planck Institute for Molecular Genetics, Berlin, Germany

[13]DZHK e.V. (German Center for Cardiovascular Research), Partner Site Hamburg/Kiel/Lübeck, Germany

[14]Institute of Neuropathology, University Medical Center Hamburg-Eppendorf, Hamburg, Germany.

[15]Core Facility for Experimental Histo-Pathology, University Medical Center Hamburg-Eppendorf, Hamburg, Germany.

[16]Present address: Herz- und Diabeteszentrum NRW, Gefäßchirurgie, Bad Oeynhausen, Germany

[17]Present address: Department of Cardiac Surgery, University Clinic Augsburg, Augsburg, Germany.

[18]Present address: Group of Immunoproteomics, Department of Immunodynamics, Institute for Experimental Immunology and Imaging, University Duisburg-Essen, Essen, Germany.

SUPPLEMENTAL RESULTS

Copy number variation (CNV) analysis

To examine whether CNVs such as duplications and deletions may contribute to the clinical manifestations in our patients, we performed CNV analysis on exome data. This resulted in one shared CNV between patients 1, 2, and 3 in family 1 (Figure 1): the deletion chr9:70,108,211-70,126,415 (hg38) that includes 4 exons of the gene *MAMDC2*. The unaffected brother of the index also has this deletion. *MAMDC2* has been associated with Kabuki syndrome (1) and truncating, loss-of-function variants in *MAMDC2* have been reported to underlie an autosomal-dominant myopathy (2). MAMDC2 (MAM domain containing 2) protein is predicted to be secreted and has been detected in extracellular compartments but also retained intracellularly in different cell lines (2, 3). MAMDC2 function has been investigated in different cancer types and an involvement of MAMDC2 in antiviral response in microglia has been reported (2, 3). *MAMDC2* has a pLI (probability of being loss-of-function intolerant) score of 0 and a LOEUF ("loss-of-function observed/expected upper bound fraction") score of 0.86 in the gnomAD database (4), suggesting tolerance to inactivation. Taken together, this information led us to not prioritize *MAMDC2* as a candidate gene for clinical manifestations in family 1. Patient 4 and 5 (family 2, Figure 1) also share one CNV: the deletion chr2:87,785,075-87,792,341 (hg38) that comprises 4 exons of the *RGPD2* gene. This gene has not been linked to any inherited disease and its function has not been studied. It is predicted to contribute to GTPase activator activity and to be involved in NLS (nuclear localization signal)-bearing protein import into nucleus (retrieved from the Alliance of Genome Resources, URL: https://www.alliancegenome.org; 2025-06-15, Alliance release 8.1.0) (5). Except in blood, testis, placenta and liver, *RGPD2* is weakly expressed (gtexportal.org/home/gene/RGPD2, proteinatlas.org/ENSG00000185304-RGPD2; both accessed 2024/12/4). *RGPD2* has a pLI score of 0.73 and a LOEUF score of 0.75 in the gnomAD database (4), suggesting medium tolerance to inactivation. Taken together, this information led us not to consider this CNV as a candidate risk allele for TAAD spectrum disorders.

FBN1 whole gene analysis

To exclude non-coding *FBN1* variants that potentially affect *FBN1* transcripts, we performed whole-genome sequencing in patients 1 and 5 and filtered for *FBN1* variants in coding and non-coding regions of the *FBN1* gene as well as in 10 kB *FBN1* up- and downstream sequence each. We did not detect any exonic *FBN1* variant in patients 1 and 5, which is in line with data from exome analyses as described in the main manuscript. In total we identified 256 *FBN1* variants in non-coding regions including 222 intronic variants, 5 3'-UTR variants, 11 downstream gene variants, 6 intergenic variants, 7 non-coding transcript variants, and 5 upstream regulatory-region variants. To evaluate *FBN1* variants, we applied SpliceAI, an open-source deep learning splicing prediction algorithm with a high ability to predict

splicing defects caused by DNA variations (6). SpliceAI predicts delta scores ranging from 0 to 1, which can be interpreted as the probability of the variant being splice-altering. A cutoff of 0.2 leads to high recall, a cutoff of 0.5 is recommended, and a cutoff of 0.8 leads to high precision (6). The highest delta score for an identified *FBN1* variant was 0.16 in patient 1 (for rs9806595) and 0.13 in patient 5 (for rs3929051). Thus, SpliceAI does not suggest consequence on splicing for any *FBN1* variant. The calculation of the maximal population frequency (MPF) for pathogenic *FBN1* variants resulted in 0.00003 (0.003%) (Supplemental Table 1). We detected only one intronic variant with an allele frequency (gnomAD v4.1) below the calculated MPF (0.00000657142 for rs772037811) and two moderately above the MPF (0.000403573 for rs10587361, 0.000899446 for rs147373570) in patient 5. Patient 1 also carries the *FBN1* variant rs10587361 with an allele frequency of 0.000403573 (gnomAD v4.1). All other FBN1 variants detected in the two patients have allele frequencies (gnomAD v4.1) > 0.002. In addition to applying SpliceAI, we predicted splicing effects for the three rarest variants identified in the two patients by using four different prediction algorithms including SpliceSiteFinder-like, MaxEntScan, NNSPLICE, and GeneSplicer. None of these algorithms indicated any effects of the three *FBN1* variants rs772037811, rs10587361, and rs147373570 on *FBN1* transcripts. In summary, our detailed analyses did not predict any negative consequences on splicing for the detected non-coding *FBN1* variants.

Molecular modelling and conservation analysis - detailed description

CDKL1 is considered to be a member of the cell division control protein 2 (CDC2)-related serine-threonine protein kinase family and shows the conserved MAP kinase Thr-Xaa-Tyr (Thr-Asp-Tyr) dual phosphorylation motif (Supplemental Figure 1) (7, 8). We explored the structural impact of the predicted CDKL1 amino acid changes by using the predicted 3D structure of human CDKL1 (AlphaFold AF-Q00532-F1-model_v4; amino acids Met^{1}–Ile^{357}) in complex with ATP as a template (AlphaFill model AF-Q00532-F1-model_v1) (9-11). Figure 2A shows the spatial position of affected CDKL1 amino acids Cys^{143}, Ser^{206}, and Thr^{135} in relation to conserved protein motifs, secondary structure elements and the protein surface. All affected amino acids are conserved (Figure 2B).

CDKL1 shares homologies with the cell cycle kinases CDK2 and CDK1, and substitutions of the Cys^{143}-neighbouring homologous amino acids in CDK2 and CDK1 (Asp^{145} and Asp^{146}, respectively) within the invariant DFG motif (Supplemental Figure 1) that is important for magnesium binding and protein catalysis (12-14), resulted in loss of kinase activity (15, 16). Bordering the DFG motif Cys^{143} is the first amino acid of the activation loop that is conserved within protein kinases and undergoes large conformational changes to switch kinases between its inactive and active forms (for details see Supplemental Figure 1 and respective legend) (17). In detail, Cys^{143} lies within the so-called magnesium binding loop that chelates two divalent Mg^{2+} ions; many protein kinases require Mg^{2+} ions for optimum

catalysis (14, 18). Specifically, Asp^{144} and Asn^{131} (for the homologues CDK2 kinase) have been demonstrated to be crucial in chelating Mg^{2+} that positions ATP for phosphotransfer (14, 18). Thus, the magnesium binding loop occupies a critical position in the kinase active site and distortions of the loop prohibits correct positioning of the ATP phosphates resulting in decreased kinase activity (14). Molecular replacement of Cys^{143} for an arginine resulted in loss of one VDW contact with the phosphate donor ATP and changes of >25 intramolecular interactions (contacts) with Val^{64}, His^{124}, Asn^{131}, Leu^{133}, Leu^{142}, and Asp^{144} (Figure 2C, Supplemental Figure 2A). The most prevalent rotamer of Arg^{143} strongly collided with Leu^{63}, Val^{64}, and Leu^{133} (Figure 2C, Supplemental Figure 2A), and at minimum 18 VDW clashes were determined by ChimeraX side-chain rotamer analysis. Finally, substitution of the polar, uncharged Cys^{143} by a basic, positively charged arginine may change both surface hydrophobicity and surface electrostatic potential (Supplemental Figure 2B). Taken together these facts and predictions strongly suggest that the p.Cys143Arg change interferes with ATP phosphate positioning and/or protein catalysis.

Ser^{206} is within the GKSDVD protein binding motif that is common in kinases of the CDK family (where it is referred to as GDSEID motif) and implicated in regulator/substrate interaction (Figure 2A, Supplemental Figure 1) (19-21). Moreover, Ser^{206} localizes at the end of an amino acid stretch between the αF and αG helices (Supplemental Figure 3A), which stabilizes the activation loop (Supplemental Figure 1) via intramolecular interactions and, thereby, enables correct substrate positioning (22). Ser^{206} forms >20 intramolecular interactions (VDW contacts) with Arg^{165} in the activation segment and with Val^{208}, Asp^{209}, and Gln^{210} (Supplemental Figure 3A). Molecular replacement of Ser^{206} for leucine resulted in modification of these intramolecular interactions. The most prevalent rotamer of Leu^{206} did not clash with atoms from neighboring amino acids (Supplemental Figure 3A); naturally, other rotamers did, as determined by ChimeraX side-chain rotamer analysis (data not shown). Substitution of the uncharged, polar and hydrophilic Ser^{206} by an uncharged, nonpolar hydrophobic leucine did not affect the surface electrostatic potential, however, surface hydrophobicity has been completely reversed (Figure 2C, Supplemental Figure 3B). Surface hydrophobicity is crucial for protein folding and functions (e.g., protein-protein binding properties) (23). Taken together, our modelling data indicate that the CDKL1 p.Ser206Leu alteration may affect interactions with and/or positioning of yet unreported regulators/substrates.

Thr^{135} borders the serine/threonine protein kinases active-site signature. This protein motif is located in the central part of the catalytic domain and contains a conserved aspartic acid residue (Asp^{126}) that is important for the catalytic activity of the enzyme (Figure 2A, Supplemental Figure 1) (24, 25). Thr^{135} forms >20 intramolecular interactions (VDW contacts) with 6 neighboring amino acids (Supplemental Figure 4A). Two of these, Ile^{134} and Cys^{83} are active-site amino acids, involved in phosphate donor positioning (Cys^{83}) and protein catalysis (Figure 2C, Supplemental Figures 1 and 4B). Moreover, Thr^{135}

localizes in the magnesium binding loop (see above) suggesting a structural function in positioning of Mg^{2+}, and thereby, ATP (Supplemental Figure 1). Molecular replacement of Thr^{135} for the most prevalent methionine rotamer resulted in loss of >10 intramolecular interactions (Supplemental Figure 4A), including the one with Cys^{83} (Figure 2C, Supplemental Figure 4B). Electrostatic potential of CDKL1 surface was not affected; however, surface hydrophobicity might be marginally altered by the p.Thr135Met change because of the opposing hydrophobicity of threonine and methionine (Supplemental Figure 4C). Taken together, due to the spatial proximity of amino acid 135 and the active site of CDKL1, the p.Thr135Met alteration possibly affects the catalytic activity of the kinase.

In summary, structural and physicochemical *in silico* calculations suggest adverse consequences on specific protein features for all identified CDKL1 variants.

CDKL1 expression in human tissue

CDKL1/*CDKL1* is expressed in various human whole tissues with rather low levels and low tissue specificity (proteinatlas.org and www.gtexportal.org, both accessed September 2023) (26, 27). In detail, immunohistological stainings of tissue sections suggested CDKL1 expression in epithelial and endothelial cells, adipocytes, melanocytes, and, in addition, in the walls of blood vessels (Supplemental Figure 5) (27).

Specificity validation of CDKL1 antibodies

We analyzed the expression of CDKL1 by immunohistological stainings of aortic tissue from three patients with TAAD and two control individuals (Figure 3, Supplemental Figure 6). For that, we applied two different CDKL1-specific antibodies: antibody 1 (AB1), Merck Sigma-Aldrich, HPA059605; and antibody 2 (AB2), Thermo Fisher Scientific, PA5-101142. We validated these two antibodies as follows: (i) by IHC stainings of RT4 cells expressing CDKL1 vs. A10 cells that do not express CDKL1 (for AB1 and AB2) (Supplemental Figure 7A); (ii) by IHC stainings of HEK293T cells transfected with EGFP-$CDKL1^{WT}$ construct vs. untransfected HEK293T controls (for AB1 and AB2) (Supplemental Figure 7A); (iii) by IHC staining of consecutive sections of diseased aortic tissue with two different antibodies, AB1 and AB2, which show a congruent CDKL1 expression pattern (Supplemental Figure 7A); (iv) by immunoblotting of lysates from CDKL1-positive (RT4 and Colo205) and CDKL1-negative (A10 and HeLa) cell lines as well as from HEK293T cells transfected with EGFP-tagged or HA-tagged CDKL1 construct (for AB1 and AB2) (Supplemental Figure 7B). Taken together, the results from immunohistochemistry and from immunoblotting were in good agreement, supporting the specificity of the anti-CDKL1 antibodies.

Control experiments for functional kinase profiling

We used the microarray-based PamTechnology (www.pamgene.com) to confirm that $CDKL1^{Cys143Arg}$, $CDKL1^{Ser206Leu}$, and $CDKL1^{Thr135Met}$ do interfere with CDKL1 kinase function. Purified recombinant CDKL1 wild type, p.Thr135Met-, p.Cys143Arg-, and p.Ser206Leu protein variants were applied to kinase

reactions on microarrays and phosphorylation of 144 potential serine/threonine substrate peptides was quantified. Control experiments demonstrated that changes in phosphorylation depend on ATP as well as kinase concentration (Supplemental Figure 9, A and B). Quality control (see Supplemental Methods) resulted in 71 analyzable peptides over the entire experiment. Information on de- or increased phosphorylated peptide sequences is given in the Supplemental Table 2.

Differential quantitative proteomics (MS/MS)

Subsequent MS/MS we quantified 1,304 proteins (Supplemental Table 3). Of these, 626 proteins were identified as ANOVA significant (q-value <0.05, Benjamini-Hochberg FDR) between all compared variants (Figure 6A, Supplemental Table 3). Direct comparisons of two variants each resulted in the following numbers of statistically significant differential abundant proteins [Students t-testing p-value <0.05 and FoldChange (FC) difference > 1.5]: 243 proteins between EV and CDKL1^{WT}, 227 proteins between CDKL1^{Cys143Arg} and CDKL1^{WT}, 437 proteins between CDKL1^{Ser206Leu} and CDKL1^{WT}, 15 proteins between CDKL1^{Thr135Met} and CDKL1^{WT} and 74 proteins between CDKL1^{Lys33Arg} and CDKL1^{WT} (Figure 6A, Supplemental Figure 10, Supplemental Table 3).

Analysis of candidate CDKL1-dependent signaling pathways

Ciliary dysfunction has become the focus of research into the pathogenesis of various human hereditary diseases that typically affect multiple organ systems; this underscores the importance of the cilium in human health and development (28, 29). It has been demonstrated that CDKL1 regulates the length of primary (non-motile) cilia, organelles found in most eukaryotic cells that perform essential roles in human sensory physiology, cell signaling, and development (30, 31). Primary cilia are responsible for blood-flow-driven Notch activation in arterial vessels and vascular myogenesis of zebrafish embryos (32). Therefore, we monitored activation of NOTCH1 by measuring expression levels of cleaved NOTCH1 (Nicd) and its targets HES1, Cyclin D3, and c-Myc. Mutant CDKL1 p.Cys143Arg, p.Ser206Leu, p.Thr135Met, or p.Lys33Arg did not affect NOTCH1 signaling in transiently transfected HEK293T cells cultivated under basal growth conditions (Supplemental Figure 12A). Recent findings suggested a prominent function for canonical and non-canonical WNT signaling in primary cilia assembly and disassembly (33). We therefore measured phosphorylation levels of β-catenin (CTNNB1; phospho-Thr^{41}/Ser^{45}) within the canonical WNT signaling branch and of dishevelled segment polarity protein 2 (DVL2; phospho-Ser^{143}) within the non-canonical WNT signaling axis in HEK293T cells ectopically expressing EGFP-tagged CDKL1 wild type, p.Cys143Arg, p.Ser206Leu, p.Thr135Met, or kinase-dead p.Lys33Arg. Mutant CDKL1 variants did not affect β-catenin (Thr^{41}/Ser^{45}) and DVL2 (Ser^{143}) phosphorylation levels (Supplemental Figure 12B). Accordingly, expression of Wnt downstream effector axin2 was not altered upon knockdown of Cdkl1 in zebrafish embryos (Figure 8B). We conclude that CDKL1 dysfunction does not interfere with WNT signaling. Ciliary signaling is mediated by the

hedgehog (HH) pathways, which act as master regulators of ciliary protein transit and are essential for normal embryonic development (34, 35). Down-regulation of zebrafish *cdkl1* led to decreased sonic hedgehog (SHH) expression in the floor plate, which could be rescued by co-injection with *cdkl1* mRNA (36). We investigated the effects of human CDKL1 mutants on signaling via SHH and its targets SUFU, GLI1, and GLI3 in HEK293T cells. Mutant CDKL1 p.Cys143Arg, p.Ser206Leu, p.Thr135Met, or p.Lys33Arg did not affect any expression level of the tested signaling proteins in transiently transfected HEK293T cells (Supplemental Figure 12C).

It has been reported, that knockdown of CDKL1 resulted in reduction of cell cycle agonists CDK2, CDK4, and Cyclin D1 (CCND1) and upregulation of cell cycle inhibitor $p21^{Waf1/Cip1}$ (CDKN1A) in tumor cell lines (37, 38). We measured expression and/or phosphorylation of CDK2, Cyclin D1, Cyclin E1 (CCNE1), and $p21^{Waf1/Cip1}$. We detected no significant differences in the phosphorylation and/or expression of CDK2, Cyclin D1, Cyclin E1, and $p21^{Waf1/Cip1}$ between HEK293T cells expressing EGFP-$CDKL1^{WT}$, $CDKL1^{Thr135Met}$, $CDKL1^{Cys143Arg}$, $CDKL1^{Ser206Leu}$, or kinase dead $CDKL1^{Lys33Arg}$ Supplemental (Figure 13, A-D). Meanwhile, the article of Li and colleagues (37) has been retracted because of concerns regarding the figures presented.

TAAD is closely associated with dysregulation of transforming growth factor-beta (TGFB) signaling (39, 40). To gain insight into the consequences of the identified CDKL1 variants on TGFB-dependent signal pathways, we measured phosphorylation levels of various downstream molecules within both canonical and non-canonical signaling pathways in cells ectopically expressing EGFP-tagged CDKL1 wild type, p.Cys143Arg, p.Ser206Leu, p.Thr135Met, or kinase-dead p.Lys33Arg. In the canonical signaling branch, we detected no significant differences in SMAD2 and SMAD3 phosphorylation levels between HEK293T cells expressing EGFP-$CDKL1^{WT}$, $CDKL1^{Thr135Met}$, $CDKL1^{Cys143Arg}$, $CDKL1^{Ser206Leu}$, or kinase dead $CDKL1^{Lys33Arg}$ (Supplemental Figure 14A). Within non-canonical signaling branches, expression of any CDKL1 variant did not significantly affect MEK1/2 phosphorylation (Ser^{218}/Ser^{222} and Ser^{222}/Ser^{226}; antibody name p-MEK1/2 $Ser^{217/221}$) and ERK1/2 phosphorylation (Thr^{202}/Thr^{185} and Tyr^{204}/Tyr^{187}; at sequence motif GFLTEYVAT; antibody name p-ERK1/2 Thr^{202}/Tyr^{204}) (Supplemental Figure 14B). To examine non-canonical TGFB-dependent signaling via PI3K-AKT, we switched to another cell line, MCF7 cells, since HEK293T cells have very high phospho (p)-AKT levels and, therefore, represent a poor model in this regard. Expression of CDKL1 p.Cys143Arg, p.Ser206Leu, p.Thr135Met, or p.Lys33Arg did not affect AKT1/2/3 ($Ser^{473/474/472}$; antibody name p-Akt Ser^{473}) phosphorylation compared to CDKL1 wild type or empty vector control (Supplemental Figure 14C).

Taken together, our data suggest that disease-associated CDKL1 variants neither considerably interfere with signaling mediated by SMAD2-SMAD3, MEK-ERK, AKT, WNT, NOTCH, and SHH, nor with cell cycle regulation.

SUPPLEMENTAL DISCUSSION

Zebrafish intersomitic vessel defects as indicator of human vasculopathy genes

Malformation of zebrafish ISV has been associated with multiple angiogenesis-related signaling pathways and genes, such as *Flt1 (vegfr1)-notch, dll4-notch*, *eng-bmp*, *slit-robo,* and *brcc3* (41-46). Clearly, several of the signaling proteins involved in these pathways are valid disease genes of human disorders of the vascular system: (i) pathogenic variants in *ENG* - a transmembrane accessory receptor for TGFB signaling - underlie telangiectasia hereditary hemorrhage type 1 (HHT1; *MIM* #187300) that is characterized by arterial aneurysm; (ii) mutations in *NOTCH 1*, *2,* and *3* cause various disorders with vascular involvement including Adams-Oliver syndrome 5 (MIM# 616028), Aortic valve disease 1 (MIM# 109730), Alagille syndrome 2 (*MIM* #610205), Hajdu-Cheney syndrome (MIM# 102500), Cerebral arteriopathy with subcortical infarcts and leukoencephalopathy 1 (MIM# 125310), and Lateral meningocele syndrome (*MIM* #130720); (iii) variants in *ROBO4* result in Aortic valve disease 3 (*MIM* #618496), that is also characterized by aneurysms of the aortic root and the ascending aorta; (iv) loss of BRCC3 deubiquitinating enzyme causes Moyamoya cerebrovascular angiopathy in humans and knockdown of the zebrafish orthologue *brcc3* resulted in defective ISV angiogenesis (47). Taken together, these data suggest that ISV malformations in zebrafish models can mimic hereditary arterial aneurysmal disease in humans.

Molecular pathology - extended discussion

We observed differences in both binding properties and ciliary localization of CDKL1 variants. In detail, CDKL1^{WT} and CDKL1^{Thr135Met} show similar protein binding patterns (Figure 6, A and B). So do CDKL1^{Cys143Arg} and CDKL1^{Lys33Arg}. CDKL1^{Ser206Leu}, however, behaves the most differently. Quantification of localization of CDKL1 variants showed that in addition to a prominent nuclear localization and a clear localization at the ciliary base for all CDKL1 variants, CDKL1^{Cys143Arg} and CDKL1^{Lys33Arg} showed an increased localization to the tip of the axoneme compared to CDKL1^{WT} and CDKL1^{Thr135Met}; in contrast, CDKL1^{Ser206Leu} did not localize to the tip of the axoneme at all (Figure 7C). It has been reported, that *C. elegans* CDKL-1 is an intraflagellar transport (IFT)-associated cargo protein that relies on IFT for its transport to cilia (31). IFT is required for the assembly and maintenance of cilia, as well as for cilium-dependent signaling. IFT complexes function as adaptors that mediate interactions between molecular motors and ciliary cargoes, facilitating cargo transport between the base and tip of the cilium (48, 49). The base of the cilium is the basal body, a structure composed of microtubule triplets, of which duplets extent and form the axoneme of the cilium; in addition, basal bodies serve as anchors, which facilitate correct positioning of the cilium within the cell (48, 49). Here we show, that - when comparing protein abundancies with control cells (EGFP^{control}) - wild type CDKL1 (CDKL1^{WT}) and CDKL1^{Thr135Met} bind to proteins involved in anterograde transport within the intraflagellar transport (IFT) system, such as

IFT52, IFT74, IFT80, IFT81, IFT88, and IFT172 (49-51). This is in line with previous results for *C. elegans* CDKL-1 protein kinase (31). Moreover, these two variants show binding with various proteins required for ciliogenesis and localizing at the ciliary base (basal body) and at the microtubule-based backbone of the cilium including TOPORS, USP9X, TRAF3IP1, CLUAP1, and CFAP20 (52-54). Only HDAC6, which localizes at the basal body and is involved in cilia disassembly, binds to CDKL1^{Thr135Met} but not with CDKL1^{WT} (Figure 6B). CDKL1^{Cys143Arg} and CDKL1^{Lys33Arg} also interact with IFT proteins (IFT52, IFT74, IFT80, IFT81, IFT88, and IFT172) and with a.m. proteins involved in ciliogenesis (TOPORS, USP9X, TRAF3IP1, CLUAP1, HDAC6, and CFAP20), when comparing protein abundances with control cells (EGFP^{control}) (Figure 6B). In addition, CDKL1^{Cys143Arg} and CDKL1^{Lys33Arg} show increased binding with various other proteins, including basal body proteins (e.g., BSG, CAV1, RAB10, RAN, and CCT8) (55-58), ciliary membrane molecules (ATP1A1, ATP1B1, ANXA1) (59), and diverse other essential proteins for cilia formation and hemostasis (e.g., TUBB4B, TUBA1A, AK1, and AK2) (49) (Figure 6B). Since CDKL1^{WT} does not bind to any of these proteins, these may represent aberrant protein complexes of CDKL1^{Cys143Arg} and CDKL1^{Lys33Arg} at the cilia base and at cargo complexes resulting in partly unphysiological subcellular distribution of these CDKL1 variants (Figure 7C). CDKL1^{Ser206Leu} shows significantly decreased binding to various IFT proteins (IFT74, IFT80, IFT81, IFT88, and IFT172) and to ciliogenesis proteins TOPORS, TRAF3IP1, and CFAP20, compared to the other CDKL1 variants (Figure 6B). Loss of binding with anterograde transport proteins IFT74, IFT80, IFT81, IFT88, and IFT172 suggests, that CDKL1^{Ser206Leu} cannot be transported to its proposed axonemal localization, and, possibly, sequesters in the cilium base (Figure 7C). Moreover and in contrast to CDKL^{WT}, CDKL1^{Ser206Leu} very strongly co-precipitated diverse ciliary proteins (e.g., BSG, CAV1, RAB10, RAN, CCT8, ATP1A1, ATP1B1, ANXA1, TUBB4B, TUBA1A, AK1, and AK2) (Figure 6B) suggesting massively altered protein-protein binding properties. In conclusion, there is a good correlation between data on cellular distribution of CDKL1 variants and binding properties of CDKL1 variants. However, it is difficult to define whether incorrect localization leads to aberrant binding or whether aberrant binding leads to incorrect localization. Given the observation that CDKL1 variants interacting with IFT proteins (i.e., CDKL1^{WT}, CDKL1^{Thr135Met}, CDKL1^{Cys143Arg}, and CDKL1^{Lys33Arg}) but not IFT binding-deficient CDKL1^{Ser206Leu} partly localize at the axoneme (Figure 7C), we speculate, that impaired binding to the IFT system result in CDKL1 mislocalization. Consequently, we assume that altered subcellular distribution due to aberrant protein interactions may represent the basic molecular defect for CDKL1^{Ser206Leu}, whereas impaired kinase function rather than mislocalization is the primary molecular defect for CDKL1^{Thr135Met}, CDKL1^{Cys143Arg}, and CDKL1^{Lys33Arg}.

Notably, although some information on the function of CDKL1 in the cilium is available now (this study; 30, 31), the role of CDKL1 in the nucleus remains largely unclear.

We showed that disease-associated, functional CDKL1 variants interfere with cilia formation and length, affect CDKL1 binding with proteins particularly involved in ciliary transport, and increase SAPK (p38) activation. The question arises why this leads to aortic disease. And whether CDKL1 function is involved in pathomechanisms already described for aortic disease, such as ECM homeostasis and VSMC biology (60), or whether our data point to an alternative pathophysiology. We put forward following hypothesis:

In the vasculature, primary cilia of VSMCs are preferentially oriented, possess proteins critical for cell-extracellular matrix (ECM) interactions, and respond to ECM proteins and mechanical stimulations (61, 62). Thus, the primary cilia of VSMCs can act as mechanochemical sensors (i.e., to sense the surrounding mechanical and chemical stimuli), thereby, affecting VSMC function and phenotype (61-69). Since VSMCs and the ECM are essential for regulating vascular tissue homeostasis, differentiation, and wound repair, cilium-mediated functions may be pivotal for maintaining vascular functional integrity (61, 69). Differentiated VSMCs in a healthy vessel are quiescent and maintain vascular tone; they are termed contractile. After mechanical damage or biochemical stimulation, VSMCs switch from the contractile (differentiated) to a synthetic (de-differentiated or proliferative) phenotype that is characterized by a low contractile profile and the overproduction of extracellular matrix components. This process is called phenotypic switching and is associated with the beginning and progression of vascular diseases (70, 71). In addition to several other signaling cascades, MAPK pathways play a critical role in VSMC phenotypic transitions, involving serine/threonine protein kinases such as ERK1/2, JNK, and p38 MAPK (72). It has become clear that the function of synthetic VSMCs is to repair or replace injured or damaged vessels (70, 73, 74). In TAAD tissue, more re-differentiated, non-contractile VSMCs were detected than in control tissue (75, 76), which can be interpreted as evidence of an involvement in the reparative remodeling of TAAD aortic tissue. Synthetic VSMCs are prone to further re-differentiate into alternative phenotypes, e.g., fibroblast-like and mesenchymal-like VSMCs that have been detected in aortic aneurysms (70, 77, 78).

Taken together we hypothesize that impaired sensory potential of primary cilia in vascular wall cells (caused by pathogenic CDKL1 variants) may induce malfunctions in VSMC phenotype switching. This may result in an unstable vascular wall structure and ultimately to the manifestation of aortic/arterial disease. Overall, this molecular/cellular pathogenesis could point to new therapeutic targets in life-threatening TAAD. In this context and underscoring the relevance of cell differentiation in the pathogenesis of TAAD it should be mentioned here that several genes/proteins involved in VSMC stability and phenotype transition have been implicated in aortic disease, with the most prominent examples involved in canonical TGFB signaling including TGFBR1, TGFBR2, TGFB2, TGFB3, SMAD2, SMAD3 and SMAD4 (39, 60, 79). TGFB signaling is required for terminal cellular differentiation often through endothelial-to-mesenchymal transition (EMT) in multiple tissue types, including

cardiovascular tissues; cell autonomous loss of canonical TGFB signalling leads to phenotypic instability, with VSMCs adopting a less contractile state that is prone to ECM degradation (60).

***CDKL1* variants have amorphic/hypomorphic, neomorphic and hypermorphic consequences**

Our zebrafish experiments suggest an amorphic (loss-of-function) or hypomorphic (partial loss-of-function) mechanism for CDKL1 missense variants, as injection of any *CDKL1* RNA (encoding wildtype, p.Cys143Arg, or p.Ser206Leu) alone did not produce significant vascular defects; notabene, here we measured short-term effects (in zebrafish embryos). We identified short-term vascular defects only upon knockdown (via morpholinos) or knockout (via Crispr/Cas9) of Cdkl1 which are clearly loss-of-function manipulations. However, we did not determine long-term effects in adult zebrafishes expressing CDKL1 missense variants, thus, long-term manifestations cannot be excluded. Furthermore, missense variants underlying long-term manifestations may have consequences on protein function other than loss of function, such as antimorphic (dominant negative), hypermorphic (gain-of-function), or neomorphic (aberrant function) effects. Accordingly, data from our biochemical and cell biological experiments suggest (i) amorphic/hypomorphic consequences regarding kinase activities of CDKL1 variants (Figure 5), (ii) amorphic/hypomorphic and neomorphic consequences regarding protein complexing properties (Figure 6; examples: amorphic: loss of interaction of $CDKL1^{Ser206Leu}$ with IFT80 compared to $CDKL^{WT}$; hypomorphic: decreased interaction of $CDKL1^{Cys143Arg}$ with IFT52 compared to $CDKL^{WT}$; neomorphic: $CDKL1^{Ser206Leu}$ but not $CDKL^{WT}$ interacts with TUBA1A), and (iii) hypermorphic consequences regarding cilia length (Figure 7) and SAPK signaling (Figure 8). In summary, the classification of variants by effect on protein function largely depends on the specific function being analyzed.

Clinical aspects

Regarding the skeletal manifestations of patients 1, 3, 4, 5, and 6, all with pathogenic variants in *CDKL1*, there is extensive literature on the function of primary cilia in skeletal development and homeostasis (80-82). Numerous skeletal phenotypes, such as polydactyly, scoliosis, a narrow thorax, and various anomalies in bone and cartilage, have been described in disorders linked to ciliary dysfunction (83). Most skeletal defects reported in the light of cilia consist of impaired bone formation (i.e., delayed mineralization or shorter bones) (80-82). There exist, however, also reports demonstrating that bone overgrowth can be associated with genes (i.e., *FGFR3*) regulating cilia (84-86). In addition, polydactyly, which could be considered excessive bone formation is linked to genes involved in cilia biology and often appears in typical ciliopathies such as Meckel Gruber syndrome (87-89). Because a critical function of the primary cilium in bone is to sense and transduce chemical and mechanical signals (90), we speculate that pathogenic CDKL1 variants underlie dysregulated mechano- or chemosensation by primary cilia in bone, which may result in aberrant cilia-derived signaling and dysregulated bone homeostasis. Notably, p38, which is hyperactive in mutant CDKL1 expressing HEK293T cells (Figure 8)

is involved in the regulation of bone formation via Wnt/β-catenin signaling (91-94). This molecular pathomechanisms may ultimately lead to the skeletal manifestations in our patients.

It is interesting that the patients described in our study do not show renal manifestations, liver manifestations, severe ocular manifestations, or central nervous system manifestations, as it is typical for many ciliopathies (95-97). On the other hand, five out of six patients have craniofacial and/or skeletal manifestations. Therefore, we would most likely assume a skeletal ciliopathy for patients with CDKL1 variants (95-97). However, if necessary we would prefer to use the term "attenuated ciliopathy" for the condition that we describe in our study, because:

(i) With few exceptions, ciliopathies are inherited in an autosomal recessive manner, and affected individuals manifest during childhood or early adolescence (95, 96). This is not the case for the patients/families described in our study.

(ii) Roughly one half and even more of pathogenic variants in diverse ciliopathy-disease genes result in putatively loss-of-gene-expression, which include nonsense variants, splice variants, small deletions, small insertions, and small indels [HGMD® Professional 2025.1 (98)]. We conclude that the absence of the respective gene product explains a large part of the underlying pathophysiology of ciliopathies. We did not detect any nonsense variant, splice variant, small deletion, small insertion, or small indel in our patients. Thus, absence of the gene product may not represent the predominant pathomechanisms in *CDKL1*-associated disease. Accordingly, data from our biochemical and cell biological experiments suggest amorphic/hypomorphic, neomorphic, and hypermorphic consequences depending on the protein properties analyzed each.

In summary, both in terms of the typical mode of inheritance (i) and the underlying pathophysiology (ii), the *CDKL1*-associated disease differs from other ciliopathies. We hypothesize that different effects on protein function (i.e., absence of gene product/loss-of-function *vs*. hypomorphic/neomorphic/hypermorphic consequences) may result in different manifestations. Loss of CDKL1 expression due to loss-of-gene-expression variants may result in manifestations typically linked to ciliary dysfunction (what still needs to be proven), whereas hypomorphic/neomorphic/hypermorphic CDKL1 variants could have a pathogenic effect only on individual organs/tissues such as the skeletal system and the vasculature. Strong "effect on protein function-phenotype correlations" have been reported for other autosomal dominant disorders, such as Marfan syndrome. There, premature termination codon variants in *FBN1* were associated with a shorter life expectancy, a high risk of aortic event, and severe scoliosis; on the other hand, variants increasing the cysteine content in fibrillin-1 have been associated with severe ophthalmologic manifestations (99). In line with these findings and hypotheses, disorders associated with cilia dysfunction have been described to exist as a phenotypic spectrum modulated by the type, number, and location of the underlying mutations (96).

(iii) All ciliopathies have in common that cilia are changed in number, length, morphology and/or function. However, ciliopathies differ greatly in their clinical representation as there are syndromic and isolated phenotypes possible. *CEP290* gene variants, for instance, can lead to isolated retinal degeneration, but may also precipitate in syndromic forms of disease including for instance brain and/or renal malformations (100). Moreover, not all ciliopathies are congenital. Autosomal dominant polycystic kidney disease for instance is often characterized by an onset later in life (usually around the 4th to 5th decade). The latter is also a perfect example for a ciliopathy, for which a “second hit” mechanism is discussed meaning that in addition to the damaging variant of a ciliary gene, a second event (i.e., unreported gene variant or environmental impact) is necessary to trigger the disease (101). Therefore and alternatively to the “effect on protein function-phenotype hypothesis” (see above), it is possible that our patient cohort was too small to detect additional, typical ciliopathy symptoms or these may become only evident later in life.

SUPPLEMENTAL METHODS

Proband recruitment and clinical examination

Probands presented at the University Heart and Vascular Center Hamburg in our specialized outpatient clinic for connective tissue diseases or in the pediatric outpatient clinic for hereditary aortopathies between 01/2016 and 03/2022. Subsequent standardized clinical examination, 3 affected members of family 1 and 320 consecutive patients were included to this study according to following criteria: clinical features were suggestive of non-syndromic TAAD, syndromic TAAD [Marfan syndrome (MFS), Loeys-Dietz syndrome (LDS), Ehlers-Danlos syndrome (EDS), congenital contractural arachnodactyly (CCA), etc.] (39, 40), or an unspecified heritable disorder of connective tissue with vascular involvement (102-104). Blood samples of the probands were obtained and exome sequencing or targeted next-generation sequencing (tNGS, i.e., gene panel sequencing) was performed. The patients with *CDKL1* variants described in this study were referred to the clinical genetics outpatient clinic of the University Medical Center Hamburg-Eppendorf for genetic counselling and further clinical examination.

Genetic testing, variant prioritization and classification

Exome sequencing

Exome sequencing (ES) was performed on genomic DNA extracted from leukocytes of 3 affected individuals from family 1 (patients P1, P2, and P3), one healthy individual from family 1 (U1), and of patients P4 and P5 (family 2) at the Center for Genomics and Transcriptomics (CeGaT, Tübingen, Germany). Coding regions including surrounding intronic sequences were isolated and enriched from DNA samples using the in-solution technology SureSelectXT Human All Exon V6 (Agilent). Captured libraries were sequenced on a HiSeq platform (Illumina) in 2x100 bp paired-end mode. On average, 89.28% and 94.04% of targeted regions were covered 30x and 20x, respectively. Trimmomatic v.0.36 (105) was used to trim sequences of sequencing adapters and suffixes of low quality (Phred quality score below 10).

Variant calling: Variant calling was performed following the Genome Analysis Toolkit's (GATK v.4.1.9.0) best practice recommendations (106). Briefly, the trimmed reads were first aligned to the human reference genome (hg38) using the Burrows-Wheeler Aligner BWA-MEM v.0.7.17 (107) and duplicate reads were removed with Picard tools v.2.20.4 (http://broadinstitute.github.io/picard). GATK was used for base quality score recalibration, calling variants using the HaplotypeCaller, joint genotyping, and variant quality score recalibration. AnnoVar v.2018-04-16 (108) was employed to annotate alterations using information from public databases [dbSNP138, ClinVar, Exome Variant Server, Genome Aggregation Database (gnomAD) and 1000 Genomes Project].

Filtering strategy: Pedigrees of affected families suggested that TAAD is either a Mendelian disorder (a variant in a gene explains a considerable part of the phenotype) or a monogenic disorder (a variant explains the complete phenotype) (40, 109-111). Therefore, we searched for unreported and very rare variants with anticipated substantial effect size shared by affected family members. Because of reduced penetrance and late onset of TAAD, non-affected family members were not included as mutation-negative controls.

Variant prioritization: Subsequent filtering on synonymous, missense, and nonsense variants, coding indels, and intronic alterations at exon-intron boundaries ranging from -10 to +10, ES data were screened for sequence variants in 66 known disease or risk genes for vascular/connective tissue disorders (Supplemental Table 1, from *ABL1* to *ZNF469*). These disorders are relatively late-onset diseases and unlikely to have a huge effect on fitness and hence allele frequencies. Therefore, only variants in these genes with an allele frequency (AF) ≤0.05, i.e., 5%, according to the gnomAD database v2.1.1 (4) were considered to be potentially disease-relevant. Variants with AF >0.05 in our in-house database were excluded, too. This cut-off AF is very conservative, since it is much higher than any predicted maximum population frequency (MPF) for respective causative variants (Supplemental Table 1). The MPF was calculated based on the respective disease prevalence, penetrance, and the genetic/allelic contribution of the respective gene/disease for all analyzed genes (Supplemental Table 1) (112). MPF calculator is available at cardiodb.org/allelefrequencyapp/ (accessed February 2022). Next, only variants that were private (absent in public database), and very rare (with an allele AF ≤0.0002, i.e., 0.02%, and no homozygotes in gnomAD controls database) were retained; this allele frequency is based on MPF of dominant causative alleles for TAAD (Supplemental Table 1): all but one (DCHS1 variants) are below 0.0002. Mutational effects were predicted with SIFT (sorting intolerant from tolerant), PolyPhen-2 (prediction of functional effects of human nsSNPs), REVEL (Rare exome variant ensemble learner), CADD (Combined Annotation Dependent Depletion), and M-CAP (Mendelian Clinically Applicable Pathogenicity) scoring systems; pathogenicity thresholds were selected according the respective authors' recommendations: SIFT (<0.05); PolyPhen2 (≥0.85), REVEL (≥0.5), CADD (≥20), M-CAP (≥0.025) (113-117). Moreover, we applied VarSome (version 11.2) for assessment of pathogenicity of variants (118). Splice site predictors were applied in case a sequence alteration (i) localized near exon intron borders, or (ii) might result in the formation of a new consensus splice site: NetGene2-2.42, neural network predictions of splice sites (119); BDGP, Berkeley Drosophila Genome Project (120); varSEAK (JSI medical systems). For confirmation and interpretation of variant calls, genomic regions of interest were visualized with the Integrative Genomics Viewer IGV v.2.11.9 (121, 122).

For CNV detection, the CNV calling algorithm ExomeDepth (123) was applied on the exome data using standard parameters. Common and false-positive CNVs were excluded by filtering against common

CNV calls from the Database of Genomic Variants (DGV) (124), the Database of Genomic Structural Variation (dbVar) (125), the gnomAD structural variant data set, and our in-house database. Correlation scores were 0.99258 for patient P1, 0.99342 for patient P2, 0.99067 for patient P3, 0.99643 for patient P4, and 0.99502 for patient P5. A candidate CNV was rejected if at least N matches with at least 95% overlap were observed in at least one of the aforementioned databases (with N=5 for DGV, N=10 for dbVar, and N=1 for the *in-house* database).

Genome sequencing

Genome sequencing was performed on genomic DNA extracted from leukocytes of patients P1 and P5 at the Center for Genomics and Transcriptomics (CeGaT, Tübingen, Germany). Library was prepared by using 100 ng DNA and TruSeq DNA Nano library preparation kit (Illumina). Library was sequenced on a NovaSeq X Plus platform (Illumina) in 2 x 151 bp paired-end mode. Q30 value of sequencing (DNA) was 93.54%. Demultiplexing of the sequencing reads was performed with Illumina bcl2fastq (version 2.20). If more output was generated for a sample than requested, the reads of this sample were downsampled to at least 20% above the ordered output. Adapters were trimmed with Skewer (version 0.2.2) (126). Quality trimming of the reads has not been performed. The MultiQC report was generated using MultiQC version 1.22.2 (https://multiqc.info/). The quality of the FASTQ files was analyzed using FastQC on Illumina's DRAGEN Bio-IT Platform (version 4.2.4). Plots were created using ggplot2 (127) in R (version 4.0.4) (R Core Team 2015; https://www.R-project.org/) (128). Sequence reads were processed with fastp (129) (v0.23.4) to remove sequences originating from sequencing adapters and sequences of low quality. The alignment was performed with BWA mem (107) (v0.7.18) and SAMtools (130) (v1.21) was used to mark putative duplicates. Variants were called using Manta (131) (v1.6.0) and Strelka2 (132) (v2.9.10). Annotation of variants was carried out with the Ensembl Variant Predictor (133) (v112).

Targeted next-generation sequencing and virtual gene panel

Genomic DNA was extracted from peripheral blood using standard laboratory procedures. In each of the 320 consecutive patients, 62 genes (inclusively *CDKL1*; Supplemental Table 1, indicated in bold font) were analyzed using a virtual gene panel on exome data or a targeted NGS (tNGS) approach. tNGS was performed at the Institute for Human Genetics, University Medical Center Hamburg-Eppendorf, Hamburg, Germany as previously reported by our group (134, 135). 40 bp of 5' and 3' intronic sequence for each exon were defined as regions of interest (ROI). These genes are either associated with thoracic aortic aneurysm/dissection-spectrum disorders or with connective tissue disease or they have crucial functions in connective tissue homeostasis (Supplemental Table 1). Enrichment of the (ROI) was performed with the Nextera™ Flex for Enrichment Kit (Illumina), according to the manufacturer's instructions. Briefly, following the fragmentation of genomic DNA, fragmented DNA was amplified, and

patient-specific (index) adapters were added by PCR. Samples from 12 patients were combined into one single hybridization mix containing target-specific capture probes. The DNA-probe hybrids were then captured with streptavidin beads, and non-targeted DNA fragments as well as unspecific binding were removed by heated washes. Next, the captured DNA library was eluted from the beads, purified, and amplified by PCR. The concentration of each library was measured by Qubit fluorometric quantification (Life Technologies). For the generation of clusters and subsequent sequencing of the targeted DNA samples on a flow cell, a sequencing reagent kit from Illumina was used. High-throughput NGS data were generated on an Illumina sequencing platform. ROI sequences were aligned to the human reference genome (hg19) and visualized and evaluated by using the Sequence Pilot module SeqNext (JSI Medical Systems). To determine true positive variants and eliminate sequencing artefacts we defined a minimal coverage of 20 reads, irrespective of the sequencing direction. Moreover, only variants covered by ≥10% of the reads, both in forward and reverse direction, were called. Synonymous, missense, and nonsense variants, coding indels, and intronic alterations at exon-intron boundaries ranging from -10 to +10 were included into the analysis. Variants with an allele frequency (AF) according to the gnomAD database v2.1.1 (4) higher than the predicted MPF of respective gene variants were excluded from further analysis. The MPF was calculated as described above and in Supplemental Table 1, resulting in an MPF of 0.0001 for *CDKL1* (112). Variants passing these filters were classified according to their likelihood for pathogenicity based on the American College of Medical Genetics and Genomics and the Association for Molecular Pathology (ACMG/AMP) guidelines on variant interpretation: pathogenic variant (PV), likely pathogenic variant (LPV), variant of uncertain significance (VUS), likely benign variant (LBV), and benign variant (BV) (136, 137). Individual ACMG/AMP criteria were assigned according to an automated *in-silico* VarSome analysis (version 11.2) (118). Criteria based on *in-silico* splicing prediction and familial segregation were added manually. VUS, LPVs, and PVs were reported.

Sanger sequencing

Familial segregation of candidate risk alleles and confirmation of *CDKL1* variants detected by exome/targeted next-generation sequencing was done on leukocyte-derived DNA by Sanger sequencing. PCR conditions and primer sequences are available on request. Amplicons were directly sequenced using the ABI BigDye Terminator Sequencing Kit (Applied Biosystems, Thermo Fisher Scientific) and an automated capillary sequencer (ABI 3500; Applied Biosystems). Sequence electropherograms were analyzed using the Sequence Pilot software (JSI Medical Systems).

Molecular Modelling

Molecular graphics were developed with UCSF ChimeraX (version 1.4) (138). For molecular replacement and analysis (e.g., surface hydrophobicity and electrostatic potential), UCSF ChimeraX

build-in tools were used. For all replaced amino acids, the most favorable/prevalent torsion of the respective side chains as predicted by UCSF ChimeraX (structure editing tools – rotamers) is shown. For modelling the structural environment of CDKL1 (NP_004187.2, NM_004196.4) amino acids 135, 143, and 206, and of CDKL1 surface, the predicted 3D structure of human CDKL1 (AlphaFold AF-Q00532-F1-model_v4; amino acids Met^{1}–Ile^{357}) in complex with ATP as a template (AlphaFill model AF-Q00532-F1-model_v1) or the crystallographic structure of CDKL1 (amino acids 2-301) (PDBe 4agu) was used (9-11, 30). Overlaps of atomic Van-der Waals (VDW) spheres within a 5.0 Å (Ångström) range were identified by using UCSF ChimeraX structure analysis tools. Contacts were defined as VDW overlaps ≥ -0.40 Å, and non-covalent clashes were defined as VDW overlaps ≥ 0.60 Å (as suggested by ChimeraX default settings). Interactions between atoms separated by four bonds or less as well as intra-residue contacts were ignored. Surface hydrophobicity was calculated by using the surface coloring feature of the UCSF ChimeraX tool (version 1.4).

Conservation analysis

To identify residues that are important for protein function, the degree of evolutionary conservation of CDKL1 amino acids was analyzed using the ConSurf server (consurf.tau.ac.il) (139). 150 sequences across species with maximal 95% identity between sequences and 35% minimal identity for homologs (i.e., stringent default settings) were extracted from the UNIREF90 database (a clustered version of the UniProt database), aligned (by using MAFFT), and scored for position specific conservation by the ConSurf Server with the Bayesian method (139, 140). The homolog search algorithm HMMER (E-value, 0.0001; No. of iterations, 1) was used (hmmer.org). Full sequence alignments are available on request.

Immunohistochemistry

Tissue samples

Aortic tissue samples were collected during replacement surgery for aortic root and/or the aorta ascendens (for patients) or aortic valve replacement (for controls). The sample aneurysm 1 comes from a male patient with ascending aortic aneurysm (55 mm), aortic valve insufficiency, and a bicuspid aortic valve; he underwent replacement surgery at the age of 57 years. The patient was not genetically examined and the etiology of the aortic aneurysm is unclear. The sample aneurysm 2 comes from a female patient with aneurysm of the aortic root, of the ascending aorta (54 mm), and of the infrarenal aorta (32 mm); she underwent replacement surgery at the age of 56 years. Genetic testing excluded class 3, 4, and 5 variants in *ACTA2*, *BGN*, *CBS*, *COL1A1*, *COL2A1*, *COL3A1*, *EFEM2*, *ELN*, *EMILIN1*, *FBN1*, *FLNA*, *LOX*, *MFAP5*, *MYH11*, *MYLK*, *NOTCH1*, *PRKG1*, *SKI*, *SLC2A10*, *SMAD2*, *SMAD3*, *SMAD4*, *SMAD6*, *TGFB2*, *TGFB3*, *TGFBR1*, *TGFBR2,* and *THSD4*; in *FBN2* a class 3 variant was identified. The sample aneurysm 3 comes from a male patient with an ascending aortic aneurysm (56 mm) and aortic valve stenosis. Moreover, he had disc herniation, retinal detachment, and varicose veins. He underwent

replacement surgery at the age of 65 years. The patient was not genetically examined and the etiology of the aortic aneurysm is unclear. The sample control 1 comes from a male individual with severe aortic valve insufficiency; he underwent replacement surgery at the age of 67 years. The sample control 2 comes from a female individual with severe aortic valve stenosis; she underwent replacement surgery at the age of 75 years.

Production of formalin-fixed paraffin-embedded cell blocks for antibody validation

Cells were fixed in formaldehyde, washed in PBS, scraped from the tissue culture surface, and harvested by centrifugation. Cells were post-fixed in formaldehyde solution for at least 48 h to model the fixation time of human tissues. As a positive control, HEK293T cells were transfected with EGFP-CDKL1^{WT} and harvested after 12 h as described above. Non-transfected cells served as negative control, respectively. Cell pellets were then embedded in 3% agarose and processed for dehydration and paraffin embedding using a Leica ASP300S tissue processor.
Paraffin sections of cells were cut at 2 µm and processed as described for human tissues. Transfected and the corresponding non-transfected negative cell preparations were always cut next to each other on one slide to enable identical staining conditions.

Immunohistochemical staining of human tissues and cell blocks

Human tissues were fixed in 4% buffered formaldehyde solution for at least 48 h and processed for paraffin embedding. For the detection of specific proteins, paraffin sections were cut (2 µm) and mounted. After dewaxing and inactivation of endogenous peroxidases (3% hydrogen peroxide), antibody specific antigen retrieval was performed. Immuno-histochemical staining were performed using a Ventana Benchmark XT autostainer (Ventana). Anti-CDKL1 antibodies were evaluated on paraffin-embedded cell blocks (AB1: #HPA059605, Merck; AB2: #PA5-101142, Thermo Fischer Scientific). Samples for a given analysis including negative controls were always performed in one staining run to avoid unwanted bias. For detection of smooth muscle actin (ACTA2) or the vascular marker CD31 in human tissue the antibodies #MA5-11547, (Thermo Fischer Scientific, 1:300) and #M0823 (Dako, 1:250) were used, respectively. All staining were developed using the Ultra View Universal 3,3´-Diaminobenzidine (DAB) Detection Kit (Ventana, Roche) which contains both secondary antibodies (anti-rabbit and anti-mouse), DAB stain and counter staining reagent. Slides were examined in a blinded fashion and representative images were taken with a Zeiss Axioscope 5 microscope and Axiocam 208 color camera.

scRNA-Seq analysis from public repository

Publicly available human aortic single-cell RNA-seq datasets (141, 142) were downloaded and reanalyzed for *CDKL1* expression. In short, filtered and annotated h5ad data was loaded into Seurat v5

(143) and integrated using harmony (144). Cluster annotation was validated based on differentially expressed marker genes. All plots were made using Seurat v5 and standard R packages.

Zebrafish experiments

Zebrafish maintenance and manipulation

Zebrafish were kept in a tank rack system (Tecniplast) with automatic monitoring and adjustment of the water quality and under a 14 hours light/10 hours dark cycle. Fish lines used in this study were EK and AB wild-type strains as well as Fli-GFP for visualization of blood vessels (145). Fertilized eggs were obtained by natural matings and were allowed to develop until the desired stage at 28.5 ºC. Morpholino microinjections were performed at the 1-2 cell stage using a Femtojet microcompressor, pulled capillaries and a Narishige micromanipulator (146). Antisense morpholino oligonucleotides (MO) (GeneTools) were custom synthesized based upon submitted sequences: *cdkl1* ATG MO: 5'-CTCATACTTCTCCATCGCTACAGCA, *cdkl1* splMO: 5'- TGCTGCACACACAGTGTAAAGACCT. As control a standard control MO (CTRL MO) provided by GeneTools was used. For rescue experiments, capped RNA encoding human wild-type or patient-derived, mutated *CDKL1* was co-injected with MOs as indicated. Crispr/Cas9-mediated gene editing was transiently performed by injection of two guide RNAs targeting exon 6 (5'-AGATTTGCCCGGCCACAAGG) and exon 7 (5'-GTTGATTCCTCGACATCAAC), respectively (IDT). Both guideRNAs were assembled into one single ribonucleoprotein complex (RNP) with HiFi Cas9 (IFT) according to IDT's standard protocol and injected directly into the first cell. As control eggs were injected with Cas9 only. To assess clutch quality, non-injected embryos were included in all experiments. Maintenance as well as manipulation of zebrafish were approved by the Veterinary Care Unit at Ulm University and University of Tübingen, respectively and the animal welfare commissioner of the regional board for scientific animal experiments in Tübingen, Germany. Experiments were performed according to the European Union Directive 2010/63/EU for the protection of animals used for experimental and other scientific purposes.

Verification of splice blocking efficiency

Splice blocking upon injection by the *cdkl1* splMO was verified as follows: RNA was isolated from 24 hours post fertilization (hpf) embryos (Qiagen RNeasy mini) and equal amounts were transcribed into cDNA using SuperscriptII (Thermo Fisher Scientific). PCR was then performed with the following primers: Forward 5'-TTGAGTACTGCGACCACACTG, Reverse 5'-CAGAGAGTAGCTCCGCAAAAA. Both, the band of the expected size as well as the larger band were excised and sequenced. As control *gapdh* was assessed: Forward: 5'- ACATTAAGTGGGGTGATGCAG, Reverse: 5'- CCATCAACGGTCTTCTGTGTT. In addition, *cdkl1* content was analysed by qPCR on a Roche LC480 cycler and the Universal Probe system (Roche, Mannheim, Germany) and the Luna® Universal Probe qPCR Master Mix (NEB): *cdkl1* (left

primer: 5'- ggaataaagacaccggacaga; right primer: 5'- ttcaccaggtttggatgtttt, UP29), *b2m* (left primer: 5'-acatcactgtacaggggaaagtc; right primer: 5'- tccgttcttcagcagttcaa, UP65).

Verification of Crispr efficiency

Individual embryos injected with an RNP containing both gRNAs and such injected with Cas9 only were digested in 50 µl 50 mM NaOH at 95 ºC for 5 minutes, cooled down quickly, and buffered by adding 5 µl 1M Tris pH 8. 5 µL of each digest per 10 µl reaction volume was used for amplification of a 610 band using the following primers: Forward 5'-TGCCAGGATTCTTAGTGCGT, reverse 5'-CACCATCTCCTGTGGCTCAG. The resulting bands were Sanger-sequenced (Microsynth).

Cloning and preparation of RNA for whole mount in situ hybridization and injection

The open reading frame of human CDKL1 was cloned into pCS2+. Site-directed mutagenesis was applied to introduce mutants as detected in patients. For in vitro transcription of capped RNA plasmids were linearized using NotI. RNA was transcribed using the SP6 mMessage mMachine Kit (Ambion, Thermo Fisher Scientific). For detection of *cdkl1* mRNA in zebrafish an 872 bp fragment of *cdkl1* (Genbank accession no. NM_001003773.1) was cloned by TOPO TA cloning into pCRII, linearized using Eco RV, and in vitro transcribed with SP6 RNA polymerase (NEB, Frankfurt, Germany) and the Roche DIG labelling mix. A *plexin D1* in situ probe (1485 bp fragment of Genbank accession no. NM_205697.2) was similarly prepared after linearization with Hind III and transcription using T7 RNA polymerase (NEB).

Whole mount in situ hybridization

Spatial expression of *cdkl1* during zebrafish development and expression of genes relevant for blood vessel development were analysed by whole mount in situ hybridization, which was performed according to standard protocols.

Imaging of zebrafish embryos

Live zebrafish embryos and embryos processed by whole mount in situ hybridization were imaged on a Leica M125 with a MC190HD or Flexacam C1 camera (Leica Microsystems). Fluorescence images of intersomitic blood vessels were imaged with a Leica M205FA equipped with a DFC365FX camera. Confocal z-stacks of the dorsal aorta of 4 dpf Fli-GFP embryos were acquired using a Leica Stellaris 5 system and the width of the aorta was measured using FIJI. Adobe Photoshop was used for image processing with adjustments (brightness, contrast, or colour balance) being equally applied to the whole image and equally to same treatment groups. Adobe Illustrator was used to assemble final figures.

qPCR analysis of genes regulating blood vessel development

Total RNA of 24 hpf zebrafish embryos was isolated with the Zymo RNA Microprep kit, which included a DNaseI step to remove genomic DNA. cDNA was generated with NEB's Protoscript II kit and the qPCR was performed using Roche's Universal Probe Library and the Luna® Universal Probe qPCR Master Mix (NEB). Primers used were: axin2 (left primer: 5'- caagtgtctctgcctccgtt; right primer: 5'- gtcgctccagtctggctatc, UP36), patched1 (left primer: 5'- ccaatgtttccttcttgctca; right primer: 5'- ctgctacaatatcgctttgacg, UP68), vegfaa (left primer: 5'- gctgagtttcacagaacacacc; right primer: 5'- catttacaggtgagggggtcc, UP29), gapdh (left primer: 5'- caggcataatggttaaagttggta; right primer: 5'- catgtaatcaaggtcaatgaatgg, UP147).

Statistical analysis of zebrafish data

For statistical analyses Prism 7 (GraphPad) was used. The data was first analysed for normal distribution, before a statistical test (parametric or non-parametric) as indicated in the figure legends was applied. Experimental groups with more than two conditions were analysed by One-way ANOVA. Otherwise t-tests were applied. A *P* value less than 0.05 was considered significant. For Figure 4E, Brown-Forsythe and Welch ANOVA test with Dunnett's T3 multiple comparisons test was applied. Individual values are: **, *P*=0.0040 (Cas9 *vs*. Cdkl1 Crispr); *, *P*=0.0223 (Cdkl1 Crispr *vs*. Cdkl1 Crispr + $CDKL1^{WT}$ RNA), 0.0202 (Cdkl1 Crispr *vs*. Cdkl1 Crispr + $CDKL1^{C143R}$ RNA), and 0.0359 (Cdkl1 Crispr *vs*. Cdkl1 Crispr + $CDKL1^{S206L}$ RNA).

Cell culture

HEK293T [purchased from the German Collection of Microorganisms and Cell Cultures GmbH (DSMZ), product no. ACC635], MCF-7 (purchased from DSMZ, product no. ACC 115), A10 (purchased from DSMZ, product no. ACC 132), HeLa (purchased from DSMZ; product no. ACC 57), and EA.hy926 [purchased from American Type Culture Collection (ATCC); product code CRL-2922] cells were cultured in Dulbecco's Modified Eagle Medium (DMEM; Gibco, Thermo Fisher Scientific) containing 10% serum (Sigma-Aldrich, Merck) and penicillin-streptomycin (100 U/ml and 100 mg/ml, respectively) (Sigma-Aldrich) at 37°C and 5% CO_2. For transient expression of CDKL1 variants, cells were transfected with pEGFP-C1-(TEV) constructs of EGFP-$CDKL1^{WT}$, EGFP-$CDKL1^{Lys33Arg}$, EGFP-$CDKL1^{Thr135Met}$, EGFP-$CDKL1^{Cys143Arg}$, EGFP-$CDKL1^{Ser206Leu}$, and EGFP-C1-TEV, or EGFP-C1 empty vector with jetOPTIMUS (1:1.6) for 24 h according to manufacturer's protocol (Polyplus transfection).

RT4 (purchased from DSMZ, product no. ACC 412) were cultured in Eagle's minimum essential medium (EMEM; Gibco, Thermo Fisher Scientific) containing 10% serum (Sigma-Aldrich, Merck), penicillin-streptomycin (100 U/ml and 100 mg/ml, respectively) (Sigma-Aldrich), and 1% non-essential amino acids (Gibco, Thermo Fisher Scientific) at 37°C and 5% CO_2.

Colo205 cells (purchased from Cytion; product number 300380) were cultured in RPMI 1640 medium (Gibco, Thermo Fisher Scientific) containing 10% serum (Sigma-Aldrich, Merck) and penicillin-streptomycin (100 U/ml and 100 mg/ml, respectively) (Sigma-Aldrich) at 37°C and 5% CO_2.

A431 (purchased from DSMZ, product no. ACC 91) were cultured in Roswell Park Memorial Institute 1640 medium (RPMI 1640 medium; Gibco, Thermo Fisher Scientific) containing 10% serum (Sigma-Aldrich, Merck) and penicillin-streptomycin (100 U/ml and 100 mg/ml, respectively) (Sigma-Aldrich) at 37°C and 5% CO_2.

RPE-1 cells (purchased from ATCC; product code CRL-4000) were cultured in DMEM/F-12 containing fetal calf serum and penicillin/streptomycin and transiently transfected using Lipofectamine 3000 (all Thermo Fisher). Two days post transfection, ciliation was induced using starvation medium (DMEM/F-12 containing only 0.1% serum). Cells were analyzed after three days of starvation.

Constructs

We amplified the coding region of wild-type *CDKL1* (NM_004196.7, NP_004187.3) using specific PCR primers and *CDKL1* cDNA in plasmid pCR4-Topo (Addgene) as a template. The coding region of Saccharomyces *cerevisiae* S288C protein kinase *IME2* (NM_001181539.1, NP_012429.1) in pcDNA3.1 was obtained from GeneScript. Purified PCR products were cloned into mammalian expression vectors pMT2sm-HA (N-terminal HA epitope), pEGFP-C1 (N-terminal EGFP epitope; Takara Bio Inc.) and/or pIREShyg3 (N-terminal EGFP epitope; Takara Bio Inc.) by using In-Fusion HD cloning Kit (Takara Bio Inc.) according to the provided protocol. *CDKL1* variants c.427T>C p.(Cys143Arg), c.404C>T p.(Thr135Met), and c.617C>T p.(Ser206Leu) were established by PCR-mediated mutagenesis using the QuikChange II site directed mutagenesis kit (Agilent) according to the provided protocol. All constructs were sequenced for integrity.

Immunoblotting

Cells were washed twice with ice-cold PBS and lysed on ice for 10 min with cell lysis buffer [50 mM Tris–HCl, pH 8.0; 150 mM NaCl; 1% Nonidet P-40; supplemented with complete Mini Protease Inhibitors (EDTA free) and PhosStop (Roche)]. After cell lysis and centrifugation (14,000 rpm, 10 min, 4°C) samples were supplemented with sample buffer, proteins were separated on SDS-polyacrylamide gels and transferred to PVDF membranes. Following blocking [20 mM Tris–HCl, pH 7.4; 150 mM NaCl; 0.1% Tween-20; 5% non-fat dry milk or 5% bovine serum albumin (BSA)] and washing (20 mM Tris–HCl, pH 7.4; 150 mM NaCl; 0.1% Tween-20), membranes were incubated in antibody solution (20 mM Tris–HCl, pH 7.4; 150 mM NaCl; 0.1% Tween-20; 5% BSA or 5% non-fat dry milk) containing appropriate antibodies overnight 4°C (Supplemental Table 4). Membranes were washed and incubated with adequate secondary antibodies (Supplemental Table 4). After final washing, proteins were visualized using the ChemiDoc MP Imaging System (Bio-Rad Laboratories, Inc.).

In vitro kinase assay

HeLa and HEK293T cells were transiently transfected with CDKL1 expression constructs (pMT2sm-HA and pEGFP-C1, respectively) using TurboFect Transfection reagent (Thermo Fisher Scientific) according to manufacturer's protocol. Cells were washed and scraped off in ice-cold PBS, pelleted, and taken up in kinase lysis buffer. HeLa cells transfected with CDKL1 in pMT2sm-HA were lysed on ice for 10 min in 500 µl kinase buffer (25 mM Tris, 150 mM NaCl, 5 mM EDTA, 1% Triton-X-100, 1 mM DTT) and clarified by centrifugation (14,000 rpm, 10 min, 4°C). Supernatants were incubated with 20 µl Pierce Anti-HA magnetic beads (Thermo Fisher Scientific) at 4°C with rotation for 120 min. Beads were magnetically separated from cell lysates and subsequently washed (3 times) with kinase buffer. Small aliquots were removed for immunoblotting. HEK293T cells transfected with CDKL1 in pEGFP-C1 were lysed on ice for 15 min in 750 µl GFP-Trap lysis buffer [50 mM Tris-HCl, 120 mM NaCl, 1 mM EDTA, 0.5% (v/v) NP-40; pH 8.0] and clarified by centrifugation (14,000 rpm, 10 min, 4°C). Supernatants were incubated with 20 µl GFP-Trap beads (ChromoTek) at 4°C with rotation for 120 min. Beads were separated from cell lysates by centrifugation and subsequently washed (3 times) with GFP-Trap lysis buffer. Small aliquots were removed for immunoblotting. To determine kinase activity of CDKL1 variants, ADP-Glo™ Kinase Assay (Promega) was used according to manufacturer's protocol. This system is luminescence-based and determines the activity of an ADP-generating enzyme by quantifying ADP produced during a kinase or ATPase reaction. Anti-HA magnetic beads or GFP-Trap beads with immobilized protein were taken up in 10 µL of kinase reaction buffer (50 mM HEPES, 10 mM $MgCl_2$, 2 mM $MnCl_2$, 0.2 mM DTT; pH 7.5). 150 µM ATP and 200 µM peptide substrate in kinase reaction buffer was added and solution was incubated at 37°C for 40 min. Since no physiological substrate of CDKL1 is known, we used a suboptimal substrate (peptide sequence RPRSPGARR), that has been determined for the S. *cerevisiae* kinases IME2 and CDC28 and applied previously to measure CDKL1 kinase activity (30, 147). We used S. *cerevisiae* IME2 and the kinase-dead variant human CDKL1 p.Lys33Arg as positive and negative control, respectively (30). The reaction mixture was then transferred to a 96-well plate and incubated for 40 min at room temperature with 25 µL of the ADP-Glo™ reagent, which terminated kinase reaction and depleted remaining ATP. Next conversion of ADP to ATP was induced by adding 50 µl of a kinase detection reagent and incubation for 40 min. The newly generated ATP was used to activate a luciferase-luciferin reaction and the resulting luminescence correlated with the amount of ADP converted in the kinase reaction and, thus, the kinase activity. Luminescence was measured with a BioTek Synergy H1 Multimode Reader (Agilent).

For statistical analyses of data from in vitro kinase assay we used Prism 8 (GraphPad). The luminescence intensities of individual measuring points in the in vitro kinase assay were normalized to the entire luminescence signal of an experiment and to the amount of immunoprecipitated protein. One-way ANOVA with a Dunnett post-hoc multiple comparison test was used. Data are presented as

box blots including 0th, 25th, 50th (median), 75th, and 100th percentiles and medians were considered significant at P-value ≤ 0.05.

Functional kinome profiling

We used a PamStation 12 (located at the UCCH Kinomics Core Facility, Hamburg, Germany) and serine/threonine kinase STK-PamChip arrays containing 144 potential serine/threonine phosphorylation substrate peptides spotted on a chip to profile CDKL1 kinase activity according to the manufacturer's instructions (PamGene International). Peptide sequences are available on request.

Recombinant CDKL1 protein for control experiments was purchased (#C43-30G, Signal Chem Biotech.); 0.05 µg, 0.1 µg, or 0.2 µg CDKL1 protein together with 400 µM ATP were used per array. To determine the effects of disease-associated variants on CDKL1 kinase profile, HEK293T cells transfected with CDKL1 variants in pEGFP-C1 were washed in PBS and lysed in 1 ml GFP-Trap lysis buffer [50 mM Tris-HCl pH 8.0, 120 mM NaCl, 1 mM EDTA, 0.5% (v/v) NP-40; 1 Complete Mini protein inhibitor cocktail tablet (Roche)] on ice for 10 minutes. Samples were centrifuged afterwards for 15 minutes at 16,000 *g* at 4°C in a precooled centrifuge. After removing small aliquots (for immunoblotting), supernatants were incubated with 25 µl GFP-Trap beads (ChromoTek) at 4°C with rotation for 120 min. Beads and bound CDKL proteins were separated from cell lysates by repeated centrifugation and washing with GFP-Trap lysis buffer (2 times) and wash buffer [10 mMTris-HCl (pH 8.0), 150 mM NaCl, 1 mM EDTA] (2 times). Samples were resuspended in 30 µl glycine elution buffer (200 mM glycine, pH 2.5) and centrifuged on ice (2,500 rpm; 2 min). Supernatants containing CDKL1 protein were transferred to new microfuge tubes containing 5 µl neutralization buffer (1 M Tris-HCl, pH 10.4). Small aliquots were removed for immunoblotting. Protein quantification was performed with the Pierce 660 Protein Assay Reagent according to the manufacturer's instructions (Thermo Fisher Scientific) and immunoblotting verified CDKL1 enrichment. Per array 0.2 µg purified protein and 400 µM ATP were applied for each CDKL1 protein variant.

Three replicates for each CDKL1 variant were analyzed and sequence-specific peptide serine/threonine phosphorylation was detected by using anti-phospho-Ser/Thr antibodies during the reaction followed by detection with a secondary antibody (polyclonal swine anti-rabbit Immunoglobulin-FITC; PamGene International). Signals were recorded using a CCD camera and Evolve software (PamGene International). After quality control (i.e., removal of low signal spots) and batch effect adjustment (ComBat), the final signal intensities were log2-transformed and used for further data and upstream kinase analyses with BioNavigator software version 5.1 (PamGene International). Data are expressed as the average signal intensity (±SD) of the analyzed peptides based on end levels of the phosphorylation curve.

Sample preparation for proteomics

HEK293T cells transfected with CDKL1 in pEGFP-C1 were washed twice with ice-cold PBS, lysed on ice for 10 min in 700 µl GFP-Trap lysis buffer [50 mM Tris-HCl, 120 mM NaCl, 1 mM EDTA, 0.5% (v/v) NP-40; pH 8.0, supplemented with complete Mini Protease Inhibitors and PhosStop (Roche)] and clarified by centrifugation (16,000 g, 10 min, 4°C). After removing small aliquots (for immunoblotting), supernatants were incubated with 20 µl GFP-Trap Magnetic Agarose beads (ChromoTek) at 4◦C with rotation for 120 min. Beads and bound CDKL proteins were magnetically separated from cell lysates and subsequently washed with GFP-Trap lysis buffer (3 times by rotation for 3 min at room temperature). After final wash, beads were supplemented with sample buffer [80 mM Tris-HCl pH 6.8, 33% glycerol, 0.3 M DTT, 6.7% SDS, 0.01% bromphenol blue], heated to 95°C and subjected to SDS-PAGE and immunoblotting as well as sample preparation for proteomics. In-gel digestion was done following established protocols (148). Shrinking and swelling was performed with 100% acetonitrile (ACN) and 100 mM NH_4HCO_3. In-gel reduction was achieved with 10 mM dithiothreitol (dissolved in 100 mM NH_4HCO_3). Alkylation was performed with 55 mM iodacetamide (dissolved in 100 mM NH_4HCO_3). Proteins in the gel pieces were digested by covering them with a trypsin solution (8 ng/µl sequencing-grade trypsin, dissolved in 50 mM NH_4HCO_3 containing 10% ACN) and incubating the mixture at 37◦C for overnight. Tryptic peptides were yielded by extraction with 2% formic acid (FA), 80% ACN. The extract was evaporated. For liquid chromatography–mass spectrometry (LC–MS/MS) analysis, samples were dissolved in 20 µl 0.1% FA.

Differential quantitative proteomics

Chromatographic separation of peptides was achieved by nano ultra-performance liquid chromatography (UPHLC, UltiMate™ 3000 RSLCnano System, Thermo Fisher Scientific) with a two-buffer system (buffer A: 0.1% FA in water, buffer B: 0.1% FA in ACN). Attached to the UHPLC was a peptide trap (100 µm × 20 mm, 100 Å pore size, 5 µm particle size, Acclaim PepMap, Thermo Fisher Scientific) for online desalting and purification followed by a 25-cm C18 reversed-phase column (75 µm × 200 mm, 130 Å pore size, 1.7 µm particle size, Peptide BEH C18; Waters, Eschborn, Germany). Peptides were separated using an 80-min gradient with linearly increasing ACN concentration from 2% to 30% ACN in 60 minutes. The eluting peptides were analyzed on a Quadrupole Orbitrap hybrid mass spectrometer (QExactive; Thermo Fisher Scientific). Here, the ions being responsible for the 12 highest signal intensities per precursor scan (1×10^6 ions, 70,000 Resolution, 240 ms fill time) were analyzed by MS/MS (higher-energy collisional dissociation (HCD) at 25 normalized collision energy, 1×10^5 ions, 17,500 resolution, 50 ms fill time) in a range of 400–1200 m/z. A dynamic precursor exclusion of 20s was used.

Raw spectra were reprocessed, using Proteome Discoverer 3.1 (Version 3.1.0.638, Thermo Fisher Scientific) against reviewed Homo sapiens FASTA database and Green Fluorescent Protein (GFP)

database. Carbamidomethylation was set as fixed modification for cysteine residues and the oxidation of methionine as well as acetylation and methionine loss of the protein N-terminus were allowed as variable modifications. A maximum number of 2 missing tryptic cleavages was set. Peptides between 6 and 144 amino acids where considered. A strict cut off [false discovery rate (FDR) <0.01] was set for peptide and protein identification.

Protein abundances were normalized in Proteome Discoverer to the abundance of GFP peptides. Data was log2 transformed. Data imputation was performed under the MNAR (missing not at random) – hypothesis in Perseus (149), to account for batch effects arising from two measurement batches. Data points were imputed from the normal distribution per sample, with a downshift of 1.8. Batch-effect reduction was performed for imputed data by using the BERT algorithm (150) in RStudio (151) in default ComBat mode. After data harmonization across batches, 1,304 proteins remained and were used for further statistical testing. For CDKL1 and GFP, the expected abundance distribution was visualized in Perseus as profile plot. ANOVA testing was performed in Perseus with Benjamini Hochberg FDR-correction. For visual purposes, corrected data was scaled across proteins, prior to hierarchical clustering based on Pearson correlation. For direct comparisons to the WT, Student's T-testing was performed with permutation-based FDR correction. All visualizations were performed in RStudio (version 4.3.3) (151) using the packages "ComplexHeatmap" (152, 153) and "ggplot2" (127). The mass spectrometry proteomics data have been deposited to the ProteomeXchange Consortium via the PRIDE partner repository with the dataset identifier PXD048044 (154). File Annotations can be found in Supplemental Table 3.

Co-Immunoprecipitation

HEK293T cells transfected with CDKL1 variants in pEGFP-C1 were washed twice with ice-cold PBS, lysed on ice for 10 min in 700 µl GFP-Trap lysis buffer [50 mM Tris-HCl, 120 mM NaCl, 1 mM EDTA, 0.5% (v/v) NP-40; pH 8.0, supplemented with complete Mini Protease Inhibitors and PhosStop (Roche)] and clarified by centrifugation (16,000 g, 10 min, 4°C). After removing small aliquots (for immunoblotting), supernatants were incubated with 20 µl GFP-Trap Magnetic Agarose beads (ChromoTek) at 4◦C with rotation for 120 min. Beads were magnetically separated from cell lysates by centrifugation and subsequently washed (3 times) with GFP-Trap lysis buffer. After final wash, beads were supplemented with sample buffer, heated to 95°C and precipitated proteins were subjected to SDS-PAGE and immunoblotting or to sample preparation for proteomics.

Immunocytochemistry

Analysis of ciliation in RPE-1 cells: Two days after transfection, full medium was replaced by starvation medium containing only 0.1% FCS to induce ciliation. After 72 hours of starvation, cells were fixed in ice-cold methanol and processed for immunofluorescence as described before (155). Antibodies:

mouse anti-acetylated tubulin (Sigma, cat.no. T6793, 1:1,000), rabbit anti-gamma tubulin (Sigma, cat.no. T5192, 1:1,000), Alexa-labeled secondary antibodies (Thermo Fisher). Cilia were imaged using a Leica Stellaris 5 confocal setup. Figures shows maximum projections of z-stacks acquired with optimized number of z-steps. For statistical evaluation of ciliation, Kruskal-Wallis test with Dunn's multiple comparison post-test (Figure 7A) and Brown-Forsythe and Welch ANOVA test with Dunnett's T3 multiple comparison test (Figure 7B) were applied.

HEK293T cells were seeded on poly-L-lysine coated coverslips (100,000 cells / 3.5 cm^2) and cultivated overnight. Cells were transfected with EGFP-C1 constructs of CDKL1 variants as described above. After 24h, cells were rinsed with PBS, fixed with 4% paraformaldehyde (Sigma-Aldrich) in PBS and washed three times with PBS. After treatment with permeabilization/blocking solution (2% BSA, 3% goat serum, 0.5% Nonidet P40 in PBS), cells were subsequently incubated in antibody solution (3% goat serum and 0.1% Nonidet P40 in PBS) containing rabbit polyclonal pericentrin antibody (Abcam; ab4448; 1:2,000) and mouse monoclonal acetylated tubulin antibody (Merck, Sigma-Aldrich; 1:800), respectively. Cells were washed with PBS and incubated with Fluorophore-conjugated secondary goat anti-mouse Alexa Fluor568 antibody (Thermo Fisher Scientific; A-11031) and goat anti-rabbit Alexa Fluor633 (Thermo Fisher Scientific; A-21071) in antibody solution. After extensive washing with PBS, cells were embedded in ProLong Diamond Antifade Mountant with DAPI (P36962, Life Technologies). Fixed samples were analyzed with a confocal laser scanning microscope (Leica TCS SP8, 63x HC PL APO Oil CS2, NA 1.4; Leica). Length of cilia was measured in multiple cells from 3 independent transfection experiments per construct: 28 cells for $EGFP^{control}$, 46 cells for EGFP-$CDKL1^{WT}$, 25 cells for EGFP-$CDKL1^{Lys33Arg}$, 20 cells for EGFP-$CDKL1^{Thr135Met}$, 32 cells for EGFP-$CDKL1^{Cys143Arg}$, and 34 cells for EGFP-$CDKL1^{Ser206Leu}$. For statistical evaluation of cilium length, Brown-Forsythe and Bartlett's ANOVA test with Tukey's multiple comparison test was applied (Figure 7C). CDKL1 localization was determined in multiple cells from 3 independent transfection experiments per construct: 17 cells for $EGFP^{control}$, 21 cells for EGFP-$CDKL1^{WT}$, 17 cells for EGFP-$CDKL1^{Lys33Arg}$, 19 cells for EGFP-$CDKL1^{Thr135Met}$, 24 cells for EGFP-$CDKL1^{Cys143Arg}$, and 28 cells for EGFP-$CDKL1^{Ser206Leu}$. For statistical evaluation Fisher's exact test as contingency analysis was applied (Figure 7C).

SUPPLEMENTAL REFERENCES

1. Kuniba H, et al. Molecular karyotyping in 17 patients and mutation screening in 41 patients with Kabuki syndrome. *J Hum Genet.* 2009;54(5):304-9.
2. Mavillard F, et al. Ablation of the carboxy-terminal end of MAMDC2 causes a distinct muscular dystrophy. *Brain.* 2023;146(12):5235-48.
3. Lee H, et al. MAM domain containing 2 is a potential breast cancer biomarker that exhibits tumour-suppressive activity. *Cell Prolif.* 2020;53(9):e12883.
4. Karczewski KJ, et al. The mutational constraint spectrum quantified from variation in 141,456 humans. *Nature.* 2020;581(7809):434-43.
5. Consortium AoGR. Updates to the Alliance of Genome Resources central infrastructure. *Genetics.* 2024;227(1).
6. Jaganathan K, et al. Predicting Splicing from Primary Sequence with Deep Learning. *Cell.* 2019;176(3):535-48 e24.
7. Taglienti CA, et al. Molecular cloning of the epidermal growth factor-stimulated protein kinase p56 KKIAMRE. *Oncogene.* 1996;13(12):2563-74.
8. Meyerson M, et al. A family of human cdc2-related protein kinases. *EMBO J.* 1992;11(8):2909-17.
9. Jumper J, et al. Highly accurate protein structure prediction with AlphaFold. *Nature.* 2021;596(7873):583-9.
10. Varadi M, et al. AlphaFold Protein Structure Database: massively expanding the structural coverage of protein-sequence space with high-accuracy models. *Nucleic Acids Res.* 2022;50(D1):D439-D44.
11. Hekkelman ML, et al.: Cold Spring Harbor Laboratory; 2021.
12. Hubbard SR. Crystal structure of the activated insulin receptor tyrosine kinase in complex with peptide substrate and ATP analog. *EMBO J.* 1997;16(18):5572-81.
13. Nagar B. c-Abl tyrosine kinase and inhibition by the cancer drug imatinib (Gleevec/STI-571). *J Nutr.* 2007;137(6 Suppl 1):1518S-23S; discussion 48S.
14. Nolen B, et al. Regulation of protein kinases; controlling activity through activation segment conformation. *Mol Cell.* 2004;15(5):661-75.
15. Hong KU, et al. Cdk1-cyclin B1-mediated phosphorylation of tumor-associated microtubule-associated protein/cytoskeleton-associated protein 2 in mitosis. *J Biol Chem.* 2009;284(24):16501-12.
16. Hu B, et al. S and G2 phase roles for Cdk2 revealed by inducible expression of a dominant-negative mutant in human cells. *Mol Cell Biol.* 2001;21(8):2755-66.
17. Huse M, Kuriyan J. The conformational plasticity of protein kinases. *Cell.* 2002;109(3):275-82.
18. Bao ZQ, et al. Briefly bound to activate: transient binding of a second catalytic magnesium activates the structure and dynamics of CDK2 kinase for catalysis. *Structure.* 2011;19(5):675-90.
19. Song H, et al. Phosphoprotein-protein interactions revealed by the crystal structure of kinase-associated phosphatase in complex with phosphoCDK2. *Mol Cell.* 2001;7(3):615-26.
20. De Bondt HL, et al. Crystal structure of cyclin-dependent kinase 2. *Nature.* 1993;363(6430):595-602.
21. Fleig UN, et al. A dominant negative allele of p34cdc2 shows altered phosphoamino acid content and sequesters p56cdc13 cyclin. *Mol Cell Biol.* 1992;12(5):2295-301.

22. Kornev AP, Taylor SS. Dynamics-Driven Allostery in Protein Kinases. *Trends Biochem Sci.* 2015;40(11):628-47.
23. Tang S, et al. Predicting Protein Surface Property with its Surface Hydrophobicity. *Protein Pept Lett.* 2021;28(8):938-44.
24. Knighton DR, et al. Crystal structure of the catalytic subunit of cyclic adenosine monophosphate-dependent protein kinase. *Science.* 1991;253(5018):407-14.
25. de Castro E, et al. ScanProsite: detection of PROSITE signature matches and ProRule-associated functional and structural residues in proteins. *Nucleic Acids Res.* 2006;34(Web Server issue):W362-5.
26. Consortium GT. Human genomics. The Genotype-Tissue Expression (GTEx) pilot analysis: multitissue gene regulation in humans. *Science.* 2015;348(6235):648-60.
27. Sjostedt E, et al. An atlas of the protein-coding genes in the human, pig, and mouse brain. *Science.* 2020;367(6482).
28. Moore ER. Primary Cilia: The New Face of Craniofacial Research. *Biomolecules.* 2022;12(12).
29. Shi W, et al. Novel functions of the primary cilium in bone disease and cancer. *Cytoskeleton (Hoboken).* 2019;76(3):233-42.
30. Canning P, et al. CDKL Family Kinases Have Evolved Distinct Structural Features and Ciliary Function. *Cell Rep.* 2018;22(4):885-94.
31. Park K, et al. CDKL kinase regulates the length of the ciliary proximal segment. *Curr Biol.* 2021;31(11):2359-73 e7.
32. Chen X, et al. Cilia Control Vascular Mural Cell Recruitment in Vertebrates. *Cell Rep.* 2017;18(4):1033-47.
33. Lee KH. Involvement of Wnt signaling in primary cilia assembly and disassembly. *FEBS J.* 2020;287(23):5027-38.
34. Ingham PW, et al. Mechanisms and functions of Hedgehog signalling across the metazoa. *Nat Rev Genet.* 2011;12(6):393-406.
35. Varjosalo M, Taipale J. Hedgehog: functions and mechanisms. *Genes Dev.* 2008;22(18):2454-72.
36. Hsu LS, et al. Zebrafish cyclin-dependent protein kinase-like 1 (zcdkl1): identification and functional characterization. *Int J Mol Sci.* 2011;12(6):3606-17.
37. Li W, et al. Downregulation of CDKL1 suppresses neuroblastoma cell proliferation, migration and invasion. *Cell Mol Biol Lett.* 2019;24:19.
38. Song Z, et al. RNAi-mediated downregulation of CDKL1 inhibits growth and colony-formation ability, promotes apoptosis of human melanoma cells. *J Dermatol Sci.* 2015;79(1):57-63.
39. Chou EL, Lindsay ME. The genetics of aortopathies: Hereditary thoracic aortic aneurysms and dissections. *Am J Med Genet C Semin Med Genet.* 2020;184(1):136-48.
40. Verstraeten A, et al. Aetiology and management of hereditary aortopathy. *Nat Rev Cardiol.* 2017;14(4):197-208.
41. Lu T, et al. Adamts18 deficiency in zebrafish embryo causes defective trunk angiogenesis and caudal vein plexus formation. *Biochem Biophys Res Commun.* 2020;521(4):907-13.
42. Chappell JC, et al. Flt-1 (vascular endothelial growth factor receptor-1) is essential for the vascular endothelial growth factor-Notch feedback loop during angiogenesis. *Arterioscler Thromb Vasc Biol.* 2013;33(8):1952-9.
43. Liu LY, et al. Motor neuron-derived Thsd7a is essential for zebrafish vascular development via the Notch-dll4 signaling pathway. *J Biomed Sci.* 2016;23(1):59.

44. Zhang D, et al. Endoglin is a conserved regulator of vasculogenesis in zebrafish - implications for hereditary haemorrhagic telangiectasia. *Biosci Rep.* 2019;39(5).
45. Lo YH, et al. GTP-Binding Protein 1-Like (GTPBP1l) Regulates Vascular Patterning during Zebrafish Development. *Biomedicines.* 2022;10(12).
46. Hirashima M. Regulation of endothelial cell differentiation and arterial specification by VEGF and Notch signaling. *Anat Sci Int.* 2009;84(3):95-101.
47. Miskinyte S, et al. Loss of BRCC3 deubiquitinating enzyme leads to abnormal angiogenesis and is associated with syndromic moyamoya. *Am J Hum Genet.* 2011;88(6):718-28.
48. Bhogaraju S, et al. Intraflagellar transport complex structure and cargo interactions. *Cilia.* 2013;2(1):10.
49. Lechtreck KF. IFT-Cargo Interactions and Protein Transport in Cilia. *Trends Biochem Sci.* 2015;40(12):765-78.
50. Pigino G. Intraflagellar transport. *Curr Biol.* 2021;31(10):R530-R6.
51. Bizet AA, et al. Mutations in TRAF3IP1/IFT54 reveal a new role for IFT proteins in microtubule stabilization. *Nat Commun.* 2015;6:8666.
52. Yu F, et al. Ciliopathies: Does HDAC6 Represent a New Therapeutic Target? *Trends Pharmacol Sci.* 2016;37(2):114-9.
53. Sanchez de Diego A, et al. Dido3-dependent HDAC6 targeting controls cilium size. *Nat Commun.* 2014;5:3500.
54. Chakarova CF, et al. TOPORS, implicated in retinal degeneration, is a cilia-centrosomal protein. *Hum Mol Genet.* 2011;20(5):975-87.
55. Rangel L, et al. Caveolin-1alpha regulates primary cilium length by controlling RhoA GTPase activity. *Sci Rep.* 2019;9(1):1116.
56. Reiter JF, et al. The base of the cilium: roles for transition fibres and the transition zone in ciliary formation, maintenance and compartmentalization. *EMBO Rep.* 2012;13(7):608-18.
57. Zhao H, et al. Ciliogenesis membrane dynamics and organization. *Semin Cell Dev Biol.* 2023;133:20-31.
58. Babbey CM, et al. Rab10 associates with primary cilia and the exocyst complex in renal epithelial cells. *Am J Physiol Renal Physiol.* 2010;299(3):F495-506.
59. Kuhlmann K, et al. The membrane proteome of sensory cilia to the depth of olfactory receptors. *Mol Cell Proteomics.* 2014;13(7):1828-43.
60. Chou E, et al. Genetics and mechanisms of thoracic aortic disease. *Nat Rev Cardiol.* 2023;20(3):168-80.
61. Hosio M, et al. Primary Ciliary Signaling in the Skin-Contribution to Wound Healing and Scarring. *Front Cell Dev Biol.* 2020;8:578384.
62. Lu CJ, et al. Non-random distribution and sensory functions of primary cilia in vascular smooth muscle cells. *Kidney Blood Press Res.* 2008;31(3):171-84.
63. Pala R, et al. The Roles of Primary Cilia in Cardiovascular Diseases. *Cells.* 2018;7(12).
64. Wu J, et al. Characterization of primary cilia in human airway smooth muscle cells. *Chest.* 2009;136(2):561-70.
65. Poole CA, et al. Confocal analysis of primary cilia structure and colocalization with the Golgi apparatus in chondrocytes and aortic smooth muscle cells. *Cell Biol Int.* 1997;21(8):483-94.
66. Qian Q, et al. Analysis of the polycystins in aortic vascular smooth muscle cells. *J Am Soc Nephrol.* 2003;14(9):2280-7.

67. Shi ZD, Tarbell JM. Fluid flow mechanotransduction in vascular smooth muscle cells and fibroblasts. *Ann Biomed Eng.* 2011;39(6):1608-19.
68. Reho JJ, et al. Smooth Muscle Cell-Specific Disruption of the BBSome Causes Vascular Dysfunction. *Hypertension.* 2019;74(4):817-25.
69. Raines EW. The extracellular matrix can regulate vascular cell migration, proliferation, and survival: relationships to vascular disease. *Int J Exp Pathol.* 2000;81(3):173-82.
70. Cao G, et al. How vascular smooth muscle cell phenotype switching contributes to vascular disease. *Cell Commun Signal.* 2022;20(1):180.
71. Toral M, et al. The NO signalling pathway in aortic aneurysm and dissection. *Br J Pharmacol.* 2022;179(7):1287-303.
72. Xin Y, et al. Elucidating VSMC phenotypic transition mechanisms to bridge insights into cardiovascular disease implications. *Front Cardiovasc Med.* 2024;11:1400780.
73. Elmarasi M, et al. Phenotypic switching of vascular smooth muscle cells in atherosclerosis, hypertension, and aortic dissection. *J Cell Physiol.* 2024;239(4):e31200.
74. Jaminon A, et al. The Role of Vascular Smooth Muscle Cells in Arterial Remodeling: Focus on Calcification-Related Processes. *Int J Mol Sci.* 2019;20(22).
75. Shen YH, et al. Stem cells in thoracic aortic aneurysms and dissections: potential contributors to aortic repair. *Ann Thorac Surg.* 2012;93(5):1524-33.
76. Cao G, et al. Single-cell RNA sequencing reveals the vascular smooth muscle cell phenotypic landscape in aortic aneurysm. *Cell Commun Signal.* 2023;21(1):113.
77. Chen PY, et al. Smooth Muscle Cell Reprogramming in Aortic Aneurysms. *Cell Stem Cell.* 2020;26(4):542-57 e11.
78. Nolasco P, et al. Impaired vascular smooth muscle cell force-generating capacity and phenotypic deregulation in Marfan Syndrome mice. *Biochim Biophys Acta Mol Basis Dis.* 2020;1866(1):165587.
79. Frismantiene A, et al. Smooth muscle cell-driven vascular diseases and molecular mechanisms of VSMC plasticity. *Cell Signal.* 2018;52:48-64.
80. Abraham SP, et al. Cilia kinases in skeletal development and homeostasis. *Dev Dyn.* 2022;251(4):577-608.
81. Huber C, Cormier-Daire V. Ciliary disorder of the skeleton. *Am J Med Genet C Semin Med Genet.* 2012;160C(3):165-74.
82. Li X, et al. Role of Primary Cilia in Skeletal Disorders. *Stem Cells Int.* 2022;2022:6063423.
83. Lai B, et al. Skeletal ciliopathy: pathogenesis and related signaling pathways. *Mol Cell Biochem.* 2024;479(4):811-23.
84. Kunova Bosakova M, et al. Regulation of ciliary function by fibroblast growth factor signaling identifies FGFR3-related disorders achondroplasia and thanatophoric dysplasia as ciliopathies. *Hum Mol Genet.* 2018;27(6):1093-105.
85. Deng C, et al. Fibroblast growth factor receptor 3 is a negative regulator of bone growth. *Cell.* 1996;84(6):911-21.
86. Colvin JS, et al. Skeletal overgrowth and deafness in mice lacking fibroblast growth factor receptor 3. *Nat Genet.* 1996;12(4):390-7.
87. Lee JE, Gleeson JG. A systems-biology approach to understanding the ciliopathy disorders. *Genome Med.* 2011;3(9):59.
88. Ferkol TW, Leigh MW. Ciliopathies: the central role of cilia in a spectrum of pediatric disorders. *J Pediatr.* 2012;160(3):366-71.

89. Taylor SP, et al. Mutations in DYNC2LI1 disrupt cilia function and cause short rib polydactyly syndrome. *Nat Commun.* 2015;6:7092.
90. Anderson CT, et al. Primary cilia: cellular sensors for the skeleton. *Anat Rec (Hoboken).* 2008;291(9):1074-8.
91. Tu X, et al. Noncanonical Wnt signaling through G protein-linked PKCdelta activation promotes bone formation. *Dev Cell.* 2007;12(1):113-27.
92. Chang J, et al. Noncanonical Wnt-4 signaling enhances bone regeneration of mesenchymal stem cells in craniofacial defects through activation of p38 MAPK. *J Biol Chem.* 2007;282(42):30938-48.
93. Bikkavilli RK, et al. p38 mitogen-activated protein kinase regulates canonical Wnt-beta-catenin signaling by inactivation of GSK3beta. *J Cell Sci.* 2008;121(Pt 21):3598-607.
94. Caverzasio J, Manen D. Essential role of Wnt3a-mediated activation of mitogen-activated protein kinase p38 for the stimulation of alkaline phosphatase activity and matrix mineralization in C3H10T1/2 mesenchymal cells. *Endocrinology.* 2007;148(11):5323-30.
95. Braun DA, Hildebrandt F. Ciliopathies. *Cold Spring Harb Perspect Biol.* 2017;9(3).
96. Tobin JL, Beales PL. The nonmotile ciliopathies. *Genet Med.* 2009;11(6):386-402.
97. Alzarka B, et al. Diseases of the primary cilia: a clinical characteristics review. *Pediatr Nephrol.* 2025;40(3):611-27.
98. Stenson PD, et al. The Human Gene Mutation Database (HGMD((R))): optimizing its use in a clinical diagnostic or research setting. *Hum Genet.* 2020;139(10):1197-207.
99. Arnaud P, et al. Clinical relevance of genotype-phenotype correlations beyond vascular events in a cohort study of 1500 Marfan syndrome patients with FBN1 pathogenic variants. *Genet Med.* 2021;23(7):1296-304.
100. Coppieters F, et al. CEP290, a gene with many faces: mutation overview and presentation of CEP290base. *Hum Mutat.* 2010;31(10):1097-108.
101. Pei Y. A "two-hit" model of cystogenesis in autosomal dominant polycystic kidney disease? *Trends Mol Med.* 2001;7(4):151-6.
102. Vanakker O, et al. The Genetics of Soft Connective Tissue Disorders. *Annu Rev Genomics Hum Genet.* 2015;16:229-55.
103. Bateman JF, et al. Genetic diseases of connective tissues: cellular and extracellular effects of ECM mutations. *Nat Rev Genet.* 2009;10(3):173-83.
104. Murphy-Ryan M, et al. Hereditary disorders of connective tissue: a guide to the emerging differential diagnosis. *Genet Med.* 2010;12(6):344-54.
105. Bolger AM, et al. Trimmomatic: a flexible trimmer for Illumina sequence data. *Bioinformatics.* 2014;30(15):2114-20.
106. McKenna A, et al. The Genome Analysis Toolkit: a MapReduce framework for analyzing next-generation DNA sequencing data. *Genome Res.* 2010;20(9):1297-303.
107. Li H, Durbin R. Fast and accurate short read alignment with Burrows-Wheeler transform. *Bioinformatics.* 2009;25(14):1754-60.
108. Yang H, Wang K. Genomic variant annotation and prioritization with ANNOVAR and wANNOVAR. *Nat Protoc.* 2015;10(10):1556-66.
109. Brownstein AJ, et al. Genes Associated with Thoracic Aortic Aneurysm and Dissection: 2018 Update and Clinical Implications. *Aorta (Stamford).* 2018;6(1):13-20.
110. Pinard A, et al. Genetics of Thoracic and Abdominal Aortic Diseases. *Circ Res.* 2019;124(4):588-606.
111. Zhang L, Wang HH. The genetics and pathogenesis of thoracic aortic aneurysm disorder and dissections. *Clin Genet.* 2016;89(6):639-46.

112. Whiffin N, et al. Using high-resolution variant frequencies to empower clinical genome interpretation. *Genet Med.* 2017;19(10):1151-8.
113. Ioannidis NM, et al. REVEL: An Ensemble Method for Predicting the Pathogenicity of Rare Missense Variants. *Am J Hum Genet.* 2016;99(4):877-85.
114. Jagadeesh KA, et al. M-CAP eliminates a majority of variants of uncertain significance in clinical exomes at high sensitivity. *Nat Genet.* 2016;48(12):1581-6.
115. Kircher M, et al. A general framework for estimating the relative pathogenicity of human genetic variants. *Nat Genet.* 2014;46(3):310-5.
116. Adzhubei IA, et al. A method and server for predicting damaging missense mutations. *Nat Methods.* 2010;7(4):248-9.
117. Vaser R, et al. SIFT missense predictions for genomes. *Nat Protoc.* 2016;11(1):1-9.
118. Kopanos C, et al. VarSome: the human genomic variant search engine. *Bioinformatics.* 2019;35(11):1978-80.
119. Hebsgaard SM, et al. Splice site prediction in Arabidopsis thaliana pre-mRNA by combining local and global sequence information. *Nucleic Acids Res.* 1996;24(17):3439-52.
120. Reese MG, et al. Improved splice site detection in Genie. *J Comput Biol.* 1997;4(3):311-23.
121. Robinson JT, et al. Variant Review with the Integrative Genomics Viewer. *Cancer Res.* 2017;77(21):e31-e4.
122. Thorvaldsdottir H, et al. Integrative Genomics Viewer (IGV): high-performance genomics data visualization and exploration. *Brief Bioinform.* 2013;14(2):178-92.
123. Plagnol V, et al. A robust model for read count data in exome sequencing experiments and implications for copy number variant calling. *Bioinformatics.* 2012;28(21):2747-54.
124. MacDonald JR, et al. The Database of Genomic Variants: a curated collection of structural variation in the human genome. *Nucleic Acids Res.* 2014;42(Database issue):D986-92.
125. Lappalainen I, et al. DbVar and DGVa: public archives for genomic structural variation. *Nucleic Acids Res.* 2013;41(Database issue):D936-41.
126. Jiang H, et al. Skewer: a fast and accurate adapter trimmer for next-generation sequencing paired-end reads. *BMC Bioinformatics.* 2014;15:182.
127. Wickham H. *ggplot2: Elegant Graphics for Data Analysis.* Springer-Verlag New York; 2016.
128. *R: A language and environment for statistical computing*. Version 4.2.0. R Foundation for Statistical Computing; 2022. https://www.R-project.org/.
129. Chen S, et al. fastp: an ultra-fast all-in-one FASTQ preprocessor. *Bioinformatics.* 2018;34(17):i884-i90.
130. Li H, et al. The Sequence Alignment/Map format and SAMtools. *Bioinformatics.* 2009;25(16):2078-9.
131. Chen X, et al. Manta: rapid detection of structural variants and indels for germline and cancer sequencing applications. *Bioinformatics.* 2016;32(8):1220-2.
132. Kim S, et al. Strelka2: fast and accurate calling of germline and somatic variants. *Nat Methods.* 2018;15(8):591-4.
133. McLaren W, et al. The Ensembl Variant Effect Predictor. *Genome Biol.* 2016;17(1):122.
134. Renner S, et al. Next-generation sequencing of 32 genes associated with hereditary aortopathies and related disorders of connective tissue in a cohort of 199 patients. *Genet Med.* 2019;21(8):1832-41.

135. Demal et al. Expanding the clinical spectrum of COL2A1 related disorders by a MASS like phenotype. *Scientific Reports.* 2022;11.
136. Biesecker LG, Harrison SM. The ACMG/AMP reputable source criteria for the interpretation of sequence variants. *Genet Med.* 2018.
137. Richards S, et al. Standards and guidelines for the interpretation of sequence variants: a joint consensus recommendation of the American College of Medical Genetics and Genomics and the Association for Molecular Pathology. *Genet Med.* 2015;17(5):405-24.
138. Pettersen EF, et al. UCSF Chimera--a visualization system for exploratory research and analysis. *J Comput Chem.* 2004;25(13):1605-12.
139. Ashkenazy H, et al. ConSurf 2016: an improved methodology to estimate and visualize evolutionary conservation in macromolecules. *Nucleic Acids Res.* 2016;44(W1):W344-50.
140. Landau M, et al. ConSurf 2005: the projection of evolutionary conservation scores of residues on protein structures. *Nucleic Acids Res.* 2005;33(Web Server issue):W299-302.
141. Chou EL, et al. Aortic Cellular Diversity and Quantitative Genome-Wide Association Study Trait Prioritization Through Single-Nuclear RNA Sequencing of the Aneurysmal Human Aorta. *Arterioscler Thromb Vasc Biol.* 2022;42(11):1355-74.
142. Pirruccello JP, et al. Deep learning enables genetic analysis of the human thoracic aorta. *Nat Genet.* 2022;54(1):40-51.
143. Hao Y, et al. Dictionary learning for integrative, multimodal and scalable single-cell analysis. *Nat Biotechnol.* 2024;42(2):293-304.
144. Korsunsky I, et al. Fast, sensitive and accurate integration of single-cell data with Harmony. *Nat Methods.* 2019;16(12):1289-96.
145. Lawson ND, Weinstein BM. In vivo imaging of embryonic vascular development using transgenic zebrafish. *Dev Biol.* 2002;248(2):307-18.
146. Tena TC, Philipp M. Assessing Smoothened-mediated Hedgehog signaling in zebrafish. *Methods Cell Biol.* 2016;132:147-64.
147. Holt LJ, et al. Evolution of Ime2 phosphorylation sites on Cdk1 substrates provides a mechanism to limit the effects of the phosphatase Cdc14 in meiosis. *Mol Cell.* 2007;25(5):689-702.
148. Shevchenko A, et al. In-gel digestion for mass spectrometric characterization of proteins and proteomes. *Nat Protoc.* 2006;1(6):2856-60.
149. Tyanova S, et al. The Perseus computational platform for comprehensive analysis of (prote)omics data. *Nat Methods.* 2016;13(9):731-40.
150. High Performance Data Integration for Large-Scale Analyses of Incomplete Omic Profiles Using Batch-Effect Reduction Trees (BERT). https://github.com/HSU-HPC/BERT.
151. Posit team: RStudio: Integrated Development Environment for R (http://www.posit.co/). 2024.
152. Gu Z, et al. Complex heatmaps reveal patterns and correlations in multidimensional genomic data. *Bioinformatics.* 2016;32(18):2847-9.
153. Gu Z. Complex heatmap visualization. *Imeta.* 2022;1(3):e43.
154. Perez-Riverol Y, et al. The PRIDE database resources in 2022: a hub for mass spectrometry-based proteomics evidences. *Nucleic Acids Res.* 2022;50(D1):D543-D52.
155. Burkhalter MD, et al. Imbalanced mitochondrial function provokes heterotaxy via aberrant ciliogenesis. *J Clin Invest.* 2019;129(7):2841-55.

156. Lolli G, et al. The crystal structure of human CDK7 and its protein recognition properties. *Structure.* 2004;12(11):2067-79.
157. Xie X, et al. Crystal structure of JNK3: a kinase implicated in neuronal apoptosis. *Structure.* 1998;6(8):983-91.
158. Brown NR, et al. The structural basis for specificity of substrate and recruitment peptides for cyclin-dependent kinases. *Nat Cell Biol.* 1999;1(7):438-43.
159. Ogawa Y, et al. Development of a novel selective inhibitor of the Down syndrome-related kinase Dyrk1A. *Nat Commun.* 2010;1:86.
160. Lu S, et al. CDD/SPARCLE: the conserved domain database in 2020. *Nucleic Acids Res.* 2020;48(D1):D265-D8.
161. Favelyukis S, et al. Structure and autoregulation of the insulin-like growth factor 1 receptor kinase. *Nat Struct Biol.* 2001;8(12):1058-63.
162. Yang J, et al. Crystal structure of an activated Akt/protein kinase B ternary complex with GSK3-peptide and AMP-PNP. *Nat Struct Biol.* 2002;9(12):940-4.
163. Madhusudan, et al. Crystal structure of a transition state mimic of the catalytic subunit of cAMP-dependent protein kinase. *Nat Struct Biol.* 2002;9(4):273-7.
164. Adham S, et al. Classical Ehlers-Danlos syndrome with a propensity to arterial events: A new report on a French family with a COL1A1 p.(Arg312Cys) variant. *Clin Genet.* 2020;97(2):357-61.
165. Morgan DO. Principles of CDK regulation. *Nature.* 1995;374(6518):131-4.
166. Fu Z, et al. Activation of a nuclear Cdc2-related kinase within a mitogen-activated protein kinase-like TDY motif by autophosphorylation and cyclin-dependent protein kinase-activating kinase. *Mol Cell Biol.* 2005;25(14):6047-64.
167. Canagarajah BJ, et al. Activation mechanism of the MAP kinase ERK2 by dual phosphorylation. *Cell.* 1997;90(5):859-69.
168. Wu SY, et al. Discovery of a novel family of CDK inhibitors with the program LIDAEUS: structural basis for ligand-induced disordering of the activation loop. *Structure.* 2003;11(4):399-410.
169. Russo AA, et al. Structural basis of cyclin-dependent kinase activation by phosphorylation. *Nat Struct Biol.* 1996;3(8):696-700.
170. Martinez AM, et al. Dual phosphorylation of the T-loop in cdk7: its role in controlling cyclin H binding and CAK activity. *EMBO J.* 1997;16(2):343-54.
171. Hubbard SR, et al. Crystal structure of the tyrosine kinase domain of the human insulin receptor. *Nature.* 1994;372(6508):746-54.
172. Van Bergen NJ, et al. CDKL5 deficiency disorder: molecular insights and mechanisms of pathogenicity to fast-track therapeutic development. *Biochem Soc Trans.* 2022;50(4):1207-24.
173. Demal TJ, et al. Expanding the clinical spectrum of COL2A1 related disorders by a mass like phenotype. *Sci Rep.* 2022;12(1):4489.
174. Wang X, et al. Germline mutations in ABL1 cause an autosomal dominant syndrome characterized by congenital heart defects and skeletal malformations. *Nat Genet.* 2017;49(4):613-7.
175. Chen CA, et al. The expanding clinical phenotype of germline ABL1-associated congenital heart defects and skeletal malformations syndrome. *Hum Mutat.* 2020;41(10):1738-44.
176. Milewicz DM, Cecchi AC. Heritable Thoracic Aortic Disease Overview. In: Adam MP, Feldman J, Mirzaa GM, Pagon RA, Wallace SE, and Amemiya A eds. *GeneReviews(®).* Seattle (WA): University of Washington; 1993.

177. Marzin P, et al. Weill-Marchesani Syndrome. In: Adam MP, Feldman J, Mirzaa GM, Pagon RA, Wallace SE, and Amemiya A eds. *GeneReviews(®)*. Seattle (WA): University of Washington; 1993.
178. Tan KL, et al. Ari-1 Regulates Myonuclear Organization Together with Parkin and Is Associated with Aortic Aneurysms. *Dev Cell.* 2018;45(2):226-44 e8.
179. Brady AF, et al. The Ehlers-Danlos syndromes, rare types. *Am J Med Genet C Semin Med Genet.* 2017;175(1):70-115.
180. Sacharow SJ, et al. Homocystinuria Caused by Cystathionine Beta-Synthase Deficiency. In: Adam MP, Feldman J, Mirzaa GM, Pagon RA, Wallace SE, and Amemiya A eds. *GeneReviews(®)*. Seattle (WA): University of Washington; 1993.
181. Malfait F, et al. Musculocontractural Ehlers-Danlos Syndrome (former EDS type VIB) and adducted thumb clubfoot syndrome (ATCS) represent a single clinical entity caused by mutations in the dermatan-4-sulfotransferase 1 encoding CHST14 gene. *Hum Mutat.* 2010;31(11):1233-9.
182. Janecke AR, et al. The phenotype of the musculocontractural type of Ehlers-Danlos syndrome due to CHST14 mutations. *Am J Med Genet A.* 2016;170A(1):103-15.
183. Malfait F, et al. Classic Ehlers-Danlos Syndrome. In: Adam MP, Feldman J, Mirzaa GM, Pagon RA, Wallace SE, and Amemiya A eds. *GeneReviews(®)*. Seattle (WA): University of Washington; 1993.
184. Barat-Houari M, et al. The expanding spectrum of COL2A1 gene variants IN 136 patients with a skeletal dysplasia phenotype. *Eur J Hum Genet.* 2016;24(7):992-1000.
185. Terhal PA, et al. A study of the clinical and radiological features in a cohort of 93 patients with a COL2A1 mutation causing spondyloepiphyseal dysplasia congenita or a related phenotype. *Am J Med Genet A.* 2015;167A(3):461-75.
186. Wang DD, et al. Mutation Spectrum of Stickler Syndrome Type I and Genotype-phenotype Analysis in East Asian Population: a systematic review. *BMC Med Genet.* 2020;21(1):27.
187. Mortier G. Stickler Syndrome. In: Adam MP, Feldman J, Mirzaa GM, Pagon RA, Wallace SE, and Amemiya A eds. *GeneReviews(®)*. Seattle (WA): University of Washington; 1993.
188. Gregersen PA, Savarirayan R. Type II Collagen Disorders Overview. In: Adam MP, Feldman J, Mirzaa GM, Pagon RA, Wallace SE, and Amemiya A eds. *GeneReviews(®)*. Seattle (WA): University of Washington; 1993.
189. Byers PH. Vascular Ehlers-Danlos Syndrome. In: Adam MP, Feldman J, Mirzaa GM, Pagon RA, Wallace SE, and Amemiya A eds. *GeneReviews(®)*. Seattle (WA): University of Washington; 1993.
190. Plaisier E, Ronco P. COL4A1-Related Disorders. In: Adam MP, Feldman J, Mirzaa GM, Pagon RA, Wallace SE, and Amemiya A eds. *GeneReviews(®)*. Seattle (WA): University of Washington; 1993.
191. Nozu K, et al. Alport Syndrome. In: Adam MP, Feldman J, Mirzaa GM, Pagon RA, Wallace SE, and Amemiya A eds. *GeneReviews(®)*. Seattle (WA): University of Washington; 1993.
192. Clemenceau A, et al. Deleterious variants in DCHS1 are prevalent in sporadic cases of mitral valve prolapse. *Mol Genet Genomic Med.* 2018;6(1):114-20.
193. Loeys B, et al. EFEMP2-Related Cutis Laxa. In: Adam MP, Feldman J, Mirzaa GM, Pagon RA, Wallace SE, and Amemiya A eds. *GeneReviews(®)*. Seattle (WA): University of Washington; 1993.

194. Duz MB, et al. A novel case of autosomal dominant cutis laxa in a consanguineous family: report and literature review. *Clin Dysmorphol.* 2017;26(3):142-7.
195. Capuano A, et al. Diagnostic Exome Sequencing Identifies a Novel Gene, EMILIN1, Associated with Autosomal-Dominant Hereditary Connective Tissue Disease. *Hum Mutat.* 2016;37(1):84-97.
196. Luyckx I, et al. Aortic aneurysm: An underestimated serious finding in the EP300 mutation phenotypical spectrum. *Eur J Med Genet.* 2019;62(2):96.
197. Park JE, et al. Identification of de novo EP300 and PLAU variants in a patient with Rubinstein-Taybi syndrome-related arterial vasculopathy and skeletal anomaly. *Sci Rep.* 2021;11(1):15931.
198. Van Maldergem L, Loeys B. FBLN5-Related Cutis Laxa. In: Adam MP, Feldman J, Mirzaa GM, Pagon RA, Wallace SE, and Amemiya A eds. *GeneReviews(®).* Seattle (WA): University of Washington; 1993.
199. Collod-Beroud G, et al. Update of the UMD-FBN1 mutation database and creation of an FBN1 polymorphism database. *Hum Mutat.* 2003;22(3):199-208.
200. Dietz H. FBN1-Related Marfan Syndrome. In: Adam MP, Feldman J, Mirzaa GM, Pagon RA, Wallace SE, and Amemiya A eds. *GeneReviews(®).* Seattle (WA): University of Washington; 1993.
201. Frederic MY, et al. The FBN2 gene: new mutations, locus-specific database (Universal Mutation Database FBN2), and genotype-phenotype correlations. *Hum Mutat.* 2009;30(2):181-90.
202. Callewaert B. Congenital Contractural Arachnodactyly. In: Adam MP, Feldman J, Mirzaa GM, Pagon RA, Wallace SE, and Amemiya A eds. *GeneReviews(®).* Seattle (WA): University of Washington; 1993.
203. Giunta C, et al. FKBP14 Kyphoscoliotic Ehlers-Danlos Syndrome. In: Adam MP, Feldman J, Mirzaa GM, Pagon RA, Wallace SE, and Amemiya A eds. *GeneReviews(®).* Seattle (WA): University of Washington; 1993.
204. Robertson S, Wade E. FLNA-Related Otopalatodigital Spectrum Disorders. In: Adam MP, Feldman J, Mirzaa GM, Pagon RA, Wallace SE, and Amemiya A eds. *GeneReviews(®).* Seattle (WA): University of Washington; 1993.
205. Chen MH, Walsh CA. FLNA Deficiency. In: Adam MP, Feldman J, Mirzaa GM, Pagon RA, Wallace SE, and Amemiya A eds. *GeneReviews(®).* Seattle (WA): University of Washington; 1993.
206. Muchtar E, et al. Restrictive Cardiomyopathy: Genetics, Pathogenesis, Clinical Manifestations, Diagnosis, and Therapy. *Circ Res.* 2017;121(7):819-37.
207. Kuang SQ, et al. FOXE3 mutations predispose to thoracic aortic aneurysms and dissections. *J Clin Invest.* 2016;126(3):948-61.
208. Shi LM, et al. GATA5 loss-of-function mutations associated with congenital bicuspid aortic valve. *Int J Mol Med.* 2014;33(5):1219-26.
209. Gu JY, et al. Novel GATA5 loss-of-function mutations underlie familial atrial fibrillation. *Clinics (Sao Paulo).* 2012;67(12):1393-9.
210. van der Linde D, et al. Birth prevalence of congenital heart disease worldwide: a systematic review and meta-analysis. *J Am Coll Cardiol.* 2011;58(21):2241-7.
211. Wei D, et al. GATA5 loss-of-function mutation responsible for the congenital ventriculoseptal defect. *Pediatr Cardiol.* 2013;34(3):504-11.
212. Yang YQ, et al. Mutational spectrum of the GATA5 gene associated with familial atrial fibrillation. *Int J Cardiol.* 2012;157(2):305-7.

213. Van Gucht I, et al. A human importin-beta-related disorder: Syndromic thoracic aortic aneurysm caused by bi-allelic loss-of-function variants in IPO8. *Am J Hum Genet.* 2021;108(6):1115-25.
214. Ziegler A, et al. Bi-allelic variants in IPO8 cause a connective tissue disorder associated with cardiovascular defects, skeletal abnormalities, and immune dysregulation. *Am J Hum Genet.* 2021;108(6):1126-37.
215. Bertoli-Avella AM, et al. Combining exome/genome sequencing with data repository analysis reveals novel gene-disease associations for a wide range of genetic disorders. *Genet Med.* 2021;23(8):1551-68.
216. Richter GT, Friedman AB. Hemangiomas and vascular malformations: current theory and management. *Int J Pediatr.* 2012;2012:645678.
217. Guo DC, et al. Genetic Variants in LRP1 and ULK4 Are Associated with Acute Aortic Dissections. *Am J Hum Genet.* 2016;99(3):762-9.
218. Inanir I, et al. Prevalence of skin conditions in primary school children in Turkey: differences based on socioeconomic factors. *Pediatr Dermatol.* 2002;19(4):307-11.
219. Guo DC, et al. LTBP3 Pathogenic Variants Predispose Individuals to Thoracic Aortic Aneurysms and Dissections. *Am J Hum Genet.* 2018;102(4):706-12.
220. Callewaert BL, Urban Z. LTBP4-Related Cutis Laxa. In: Adam MP, Feldman J, Mirzaa GM, Pagon RA, Wallace SE, and Amemiya A eds. *GeneReviews(®)*. Seattle (WA): University of Washington; 1993.
221. Mordi I, Tzemos N. Bicuspid aortic valve disease: a comprehensive review. *Cardiol Res Pract.* 2012;2012:196037.
222. Meester JAN, et al. Overlapping but distinct roles for NOTCH receptors in human cardiovascular disease. *Clin Genet.* 2018.
223. Lehman A, et al. Adams-Oliver Syndrome - RETIRED CHAPTER, FOR HISTORICAL REFERENCE ONLY. In: Adam MP, Feldman J, Mirzaa GM, Pagon RA, Wallace SE, and Amemiya A eds. *GeneReviews* University of Washington; 1993.
224. Boudin E, et al. Bi-allelic Loss-of-Function Mutations in the NPR-C Receptor Result in Enhanced Growth and Connective Tissue Abnormalities. *Am J Hum Genet.* 2018;103(2):288-95.
225. Yeowell HN, et al. Mutational analysis of the lysyl hydroxylase 1 gene (PLOD) in six unrelated patients with Ehlers-Danlos syndrome type VI: prenatal exclusion of this disorder in one family. *Hum Mutat.* 2000;16(1):90.
226. Salo AM, et al. A connective tissue disorder caused by mutations of the lysyl hydroxylase 3 gene. *Am J Hum Genet.* 2008;83(4):495-503.
227. Greene D, et al. Genetic association analysis of 77,539 genomes reveals rare disease etiologies. *Nat Med.* 2023;29(3):679-88.
228. Dhooge T, et al. More than meets the eye: Expanding and reviewing the clinical and mutational spectrum of brittle cornea syndrome. *Hum Mutat.* 2021;42(6):711-30.
229. Schoenmakers E, et al. Selenoprotein deficiency disorder predisposes to aortic aneurysm formation. *Nat Commun.* 2023;14(1):7994.
230. Schepers D, et al. The SMAD-binding domain of SKI: a hotspot for de novo mutations causing Shprintzen-Goldberg syndrome. *Eur J Hum Genet.* 2015;23(2):224-8.
231. Greally MT. Shprintzen-Goldberg Syndrome. In: Adam MP, Feldman J, Mirzaa GM, Pagon RA, Wallace SE, and Amemiya A eds. *GeneReviews(®)*. Seattle (WA): University of Washington; 1993.

232. Callewaert B, et al. Arterial Tortuosity Syndrome. In: Adam MP, Feldman J, Mirzaa GM, Pagon RA, Wallace SE, and Amemiya A eds. *GeneReviews(®)*. Seattle (WA): University of Washington; 1993.
233. Giunta C, et al. Spondylocheiro dysplastic form of the Ehlers-Danlos syndrome--an autosomal-recessive entity caused by mutations in the zinc transporter gene SLC39A13. *Am J Hum Genet.* 2008;82(6):1290-305.
234. Micha D, et al. SMAD2 Mutations Are Associated with Arterial Aneurysms and Dissections. *Hum Mutat.* 2015;36(12):1145-9.
235. Granadillo JL, et al. Variable cardiovascular phenotypes associated with SMAD2 pathogenic variants. *Hum Mutat.* 2018;39(12):1875-84.
236. Cannaerts E, et al. Novel pathogenic SMAD2 variants in five families with arterial aneurysm and dissection: further delineation of the phenotype. *J Med Genet.* 2019;56(4):220-7.
237. Zhang W, et al. Exome sequencing identified a novel SMAD2 mutation in a Chinese family with early onset aortic aneurysms. *Clin Chim Acta.* 2017;468:211-4.
238. Loeys BL, Dietz HC. Loeys-Dietz Syndrome. In: Adam MP, Feldman J, Mirzaa GM, Pagon RA, Wallace SE, and Amemiya A eds. *GeneReviews(®)*. Seattle (WA): University of Washington; 1993.
239. Loughborough WW, et al. Cardiovascular Manifestations and Complications of Loeys-Dietz Syndrome: CT and MR Imaging Findings. *Radiographics.* 2018;38(1):275-86.
240. McDonald J, Stevenson DA. Hereditary Hemorrhagic Telangiectasia. In: Adam MP, Feldman J, Mirzaa GM, Pagon RA, Wallace SE, and Amemiya A eds. *GeneReviews(®)*. Seattle (WA): University of Washington; 1993.
241. Lin AE, et al. Myhre Syndrome. In: Adam MP, Feldman J, Mirzaa GM, Pagon RA, Wallace SE, and Amemiya A eds. *GeneReviews(®)*. Seattle (WA): University of Washington; 1993.
242. Larsen Haidle J, et al. Juvenile Polyposis Syndrome. In: Adam MP, Feldman J, Mirzaa GM, Pagon RA, Wallace SE, and Amemiya A eds. *GeneReviews(®)*. Seattle (WA): University of Washington; 1993.
243. Wunnemann F, et al. Aortic Dilatation Associated With a De Novo Mutation in the SOX18 Gene: Expanding the Clinical Spectrum of Hypotrichosis-Lymphedema-Telangiectasia Syndrome. *Can J Cardiol.* 2016;32(1):135 e1-7.
244. Li Y, et al. Variants of Focal Adhesion Scaffold Genes Cause Thoracic Aortic Aneurysm. *Circ Res.* 2021;128(1):8-23.
245. Frederic MY, et al. A new locus-specific database (LSDB) for mutations in the TGFBR2 gene: UMD-TGFBR2. *Hum Mutat.* 2008;29(1):33-8.
246. Elbitar S, et al. Pathogenic variants in THSD4, encoding the ADAMTS-like 6 protein, predispose to inherited thoracic aortic aneurysm. *Genet Med.* 2021;23(1):111-22.
247. Fokkema IF, et al. LOVD v.2.0: the next generation in gene variant databases. *Hum Mutat.* 2011;32(5):557-63.
248. Landrum MJ, et al. ClinVar: improving access to variant interpretations and supporting evidence. *Nucleic Acids Res.* 2018;46(D1):D1062-D7.

SUPPLEMENTAL FIGURES AND SUPPLEMENTAL FIGURE LEGENDS

Supplemental Figure 1

```
H.sapiens          1    -----------MEKYEKIGKIGEGSYGVVFKCRNRDTGQIVAIKKFLESEDDPVIKKIALREIRMLKQLKHPNLVNLLEVFRRKRRLHLVFEYCDHTVLH    89
P.troglodytes      1    ----------MMEKYEKIGKIGEGSYGVVFKCRNRDTGQIVAIKKFLESEDDPVIKKIALREIRMLKQLKHPNLVNLLEVFRRKRRLHLVFEYCDHTVLH    90
C.lupus            1    ----------MMEKYEKIGKIGEGSYGVVFKCRNRDTGQIVAIKRFLESEDDPVIKKIALREIRMLKQLKHPNLVNLLEVFRRKRKLHLVFEYCDHTVLH    90
B.taurus           1    -----------MEKYEKIGKIGEGSYGVVFKCRNRDTGQIVAIKKFLESEDDPVIKKIALREIRMLKQLKHPNLVNLIEVFRRKRRLHLVFEYCDHTVLH    89
M.musculus         1    -----------MEKYEKIGKIGEGSYGVVFKCRNRDTGQIVAIKRFLETEDDPVIKKIALREIRMLKQLKHPNLVNLLEVFRRKRRLHLVFEYCDHTVLH    89
R.norvegicus       1    -----------MEKYEKIGKIGEGSYGVVFKCRNRDTGQIVAIKRFLETEDDPVIKKIALREIRMLKQLKHPNLVSLLEVFRRKRRLHLVFEYCHHTVLH    89
G.gallus           1    -----------MERYEKLGKVGEGSYGVVFKCRNRETGQIVAIKKFLESEEDPVIRKIALREVRMLKQLKHPNLVNLLEVFRRKRKLHLVFEYCDHTVLH    89
D.rerio            1    -----------MEKYEKISKIGEGSYGVVFKCRNKDTGQIVAIKKFVESEDDPIIKKIALREIRMLKQLKHPNLVNLMEVFRRKRKLHLVFEYCDHTVLN    89
D.melanogaster    99    FSLRRPQGSSKMDRYEKLSRLGEGSYGVVYKCRDRETGALVAVKRFVESEDDPAIRKIALREIRLLKNLKHPNLVSLLEVFRRKRRLHLVFEFCELTVLH   198
A.gambiae         72    FS--RSRGSSRMDRYEKLSRLGEGSYGVVYKCRDRETGSLVAVKRFVESEDDPAIRKIALREIRLLKNLKHPNLVCLLEVFRRKRRLHLVFEFCEHTVLH   169
C.elegans         20    LRNSSKRRCEAMDKYDRLSKLGEGSYGVVYKCKNRDTGQIVAIKKFVETEDDPHIKKIALREIRMLKQLKHQNLVGLIEVFKRNRKLHLVFELCDRTVLH   119
X.tropicalis       1    -----------MEKYEKIGKIGEGSYGVVFKCRNRDTGQIVAIKKFLESEDDPVIKKIALREIRMLKQLKHPNLVNLLEVFRRKRKLHLVFEYCDHTVLH    89

                                                                    Mg2+ binding loop
                                                                  •-----------------•
H.sapiens         90    ELDRYQRGVPEHLVKSITWQTLQAVNFCHKHNCIHRDVKPENILITKHSVIKLCDFGFARLLTGPSDYYTDYVATRWYRSPELLVGDTQYGPPVDVWAIG   189
P.troglodytes     91    ELDRYQRGVPEHLVKSITWQTLQAVNFCHKHNCIHRDVKPENILITKHSVIKLCDFGFARLLTGPSDYYTDYVATRWYRSPELLVGDTQYGPPVDVWAIG   190
C.lupus           91    ELDRHQRGVPEHLVKNITWQTLQAVNFCHKHDCIHRDVKPENILITKHSVIKLCDFGFARLLTGPSDYYTDYVATRWYRSPELLVGDTQYGPPVDVWAIG   190
B.taurus          90    ELDRHQRGVPEHLVKSITWQTLQAVNFCHKHSCIHRDVKPENILVTKQSVIKLCDFGFARLLTGPSDYYTDYVATRWYRSPELLVGDTQYGPPVDVWAIG   189
M.musculus        90    ELDRYQRGVPEPLVKNITWQTLQAVNFCHKHNCIHRDVKPENILITKQSAIKLCDFGFARLLTGPGDYYTDYVATRWYRSPELLVGDTQYGPPVDVWAIG   189
R.norvegicus      90    ELDRYQRGVPEPLVKNITWQTLQAVNFCHKHNCIHRDVKPENILITKHSVIKLCDFGFARLLTGPGDYYTDYVATRWYRSPELLVGDTQYGPPVDVWAIG   189
G.gallus          90    ELDKHPRGVPEQLVRSITWQTLQAVNFCHKHNCIHRDVKPENILITKHSVIKLCDFGFARMLTGPGDYYTDYVATRWYRSPELLVGDTQYGPPVDVWAIG   189
D.rerio           90    ELDRYPRGVPEHMVKSIIWQTLQAVNFCHKQNCIHRDVKPENILITKHQVIKLCDFGFARILTGPCDYYTDCVATRWYRAPELLVGDTQYGPPVDVWAVG   189
D.melanogaster   199    ELERHPQGCPEHLTKQICYQTLLGVAYCHKQGCLHRDIKPENILLTAQGQVKLCDFGFARMLS-PGENYTDYVATRWYRAPELLVGDTQYGTPVDVWAIG   297
A.gambiae        170    ELERHPEGCPDNLTKQITYQTIQGVAYCHKQGCLHRDIKPENILLTAQGQVKLCDFGFARMLS-PGENYTDYVATRWYRAPELLVGDTQYGTPVDVWAIG   268
C.elegans        120    ELEKNPHGVNDELIKKIIYQLLEALKFCHSHKCIHRDVKPENIFLTRNDQVKLGDFGFARIIN-TTEMYTDYVATRWYRSPELLVGDVQYGPPVDIWAVG   218
X.tropicalis      90    ELDKYPRGVPEHLVKSITWQTLQAVNFCHKHHCIHRDVKPENILITRHSVIKLCDFGFARLLTGPSDYYTDYVATRWYRAPELLVGDTQYGPPVDVWAIG   189

H.sapiens        190    CVFAELLSGVPLWPGKSDVDQLYLIRKTLGDLIPRHQQVFSTNQYFSGVK   239 ...
P.troglodytes    191    CVFAELLSGVPLWPGKSDVDQLYLIRKTLGDLIPRHQQVFSMNQYFSGVK   240 ...
C.lupus          191    CVFAELLSGVPLWPGKSDVDQLYLIRKTLGDLIPRHQQVFSMNQYFSGVK   240 ...
B.taurus         190    CVFAELLSGVPLWPGKSDVDQLYLIRKTLGDLIPRHQHVFSTNQYFSGVK   239 ...
M.musculus       190    CVFAELLSGVPLWPGKSDVDQLYLIRKTLGDLIPRHQQVFSMNQYFSGVK   239 ...
R.norvegicus     190    CVFAELLSGVPLWPGKSDVDQLYLIRKTLGDLIPRHQQVFSMNQYFSGVK   239 ...
G.gallus         190    CVFAELLSGVPLWPGKSDVDQLYLIRRTLGDLIPRHQQVFSTNQFFSGVT   239 ...
D.rerio          190    CVFAELLSGAPLWPGKSDVDQLYLIRKTLGELIPRHQQVFSTNQFFSGVC   239 ...
D.melanogaster   298    CLFAELVRGEALWPGRSDVDQLYLIRKTLGDLLPRHIQIFGQNEYFKGIT   347 ...
A.gambiae        269    CVFAELVRGDALWPGRSDVDQLYLIRRTLGDLLPRHLAIFNQNEFFKGIT   318 ...
C.elegans        219    CVYAELLTGEALWPGRSDIDQLYHIRKTLGEFLPRHISIFRTNQFFFGLS   268 ...
X.tropicalis     190    CVFAELLSGIPLWPGKSDVDQLYLIRKSQGDLIARHQQVFSTNQFFSGVS   239 ...
```

Protein Acc.	Gene	Organism
NP_004187.3	*CDKL1*	H.sapiens
XP_003314364.1	*CDKL1*	P.troglodytes
XP_851358.1	*CDKL1*	C.lupus
NP_001094586.1	*CDKL1*	B.taurus
NP_899117.1	*Cdkl1*	M.musculus
NP_001020292.1	*Cdkl1*	R.norvegicus
XP_421464.3	*CDKL1*	G.gallus
NP_001003773.1	*cdkl1*	D.rerio
NP_608950.2	*CG7236*	D.melanogaster
XP_312877.4	*AgaP_AGAP003180*	A.gambiae
NP_001256290.1	*CELE_Y42A5A.4*	C.elegans
XP_002938169.2	*cdkl1*	X.tropicalis

Supplemental Figure 1. Evolutionary conservation of CDKL1. Species, partial amino acids sequences, species-specific gene names and protein database accession numbers (www.ncbi.nlm.nih.gov/protein) are given. Alignments demonstrate high evolutionary conservation of CDKL1. Alignments were generated with NCBI HomoloGene (www.ncbi.nlm.nih.gov/homologene accessed October 2022). Orange letters indicate CDKL1 amino acids affected by variants identified in this study. Dark blue letters indicate amino acids involved in ATP binding; based on structure PDB 4AGU and on structures of other CMGC kinases bound with ATP or ATP analogs (30, 156-160). Dark blue and light blue letters together indicate protein kinases ATP-binding region and serine/threonine protein kinases active-site signatures: (i) The first region (IGEGSYGVVFKCRNRDTGQIVAIK), which is located in the N-terminal extremity of the catalytic domain, is a glycine-rich stretch of residues in the vicinity of a lysine residue, which has been shown to be involved in ATP binding. (ii) The second region (CIHRDVKPENILI) is located in the central part of the catalytic domain and contains a conserved aspartic acid residue, which is important for the catalytic activity of the enzyme (24, 25). Dark green; polypeptide substrate binding sites; based on structures of other CMGC kinases and members of the superfamily bound with their peptide substrates (18, 158, 160-163). Dark green/light green letters indicate substrate and regulator binding regions including: (i) KKIALRE putative cyclin binding site (without experimental evidence) (8, 164, 165). (ii) CDFGFARLLTGPSDYYTDYVATRW activation loop (also called A-loop and regulatory T loop). Phosphorylation within the A-loop is required for full activation of most protein kinases. The activation loop undergoes large conformational changes, which switches the kinase between its inactive and active forms. CDKs are phosphorylated in their T-loops by CDK-activating kinases. Most MAPKs contain a TxY phosphorylation motif in the activation loop which is targeted by MAP2Ks, leading to the activation of the kinase. A TDY motif is present in MAP kinases, cyclin dependent kinases (CDKs) and CDKL1-5 (17, 160, 166-170). The DFG motif located at the beginning of the activation loop is highly conserved among kinases and is important for magnesium binding and protein catalysis (13, 14). In active kinases, the DFG motif adopts an “in” conformation and the Asp residue is oriented toward bound ATP, which is able to coordinate the magnesium ion bound to the β- and γ- phosphate groups

of ATP (12). In inactive kinases, the conformation of the DFG motif is flipped outward, such that the Asp no longer coordinates the magnesium at the catalytic site (171). For the CDK2 kinase it has been demonstrated, that a second Mg^{2+} is coordinated by Asn^{131} (18). The position of the magnesium binding loop is indicated above the amino acid sequence (14, 18). (iii) The GKSDVD sequence is homologous to the GDSEID protein binding motif in kinases of the CDK family, which is implicated in regulator and substrate binding (19-21). Turquoise letters: In addition to ATP binding and substrate/regulator binding sites/regions, Leu^{88} and His^{89} (LH) belong to the so-called active site. This is based on the structures of human CDK2 and DYRK1A bound with ATP or inhibitor and substrate peptides, and on the structures of other CMGC kinases, bound with ATP or ATP analogs (18, 156-158, 160).

Supplemental Figure 2

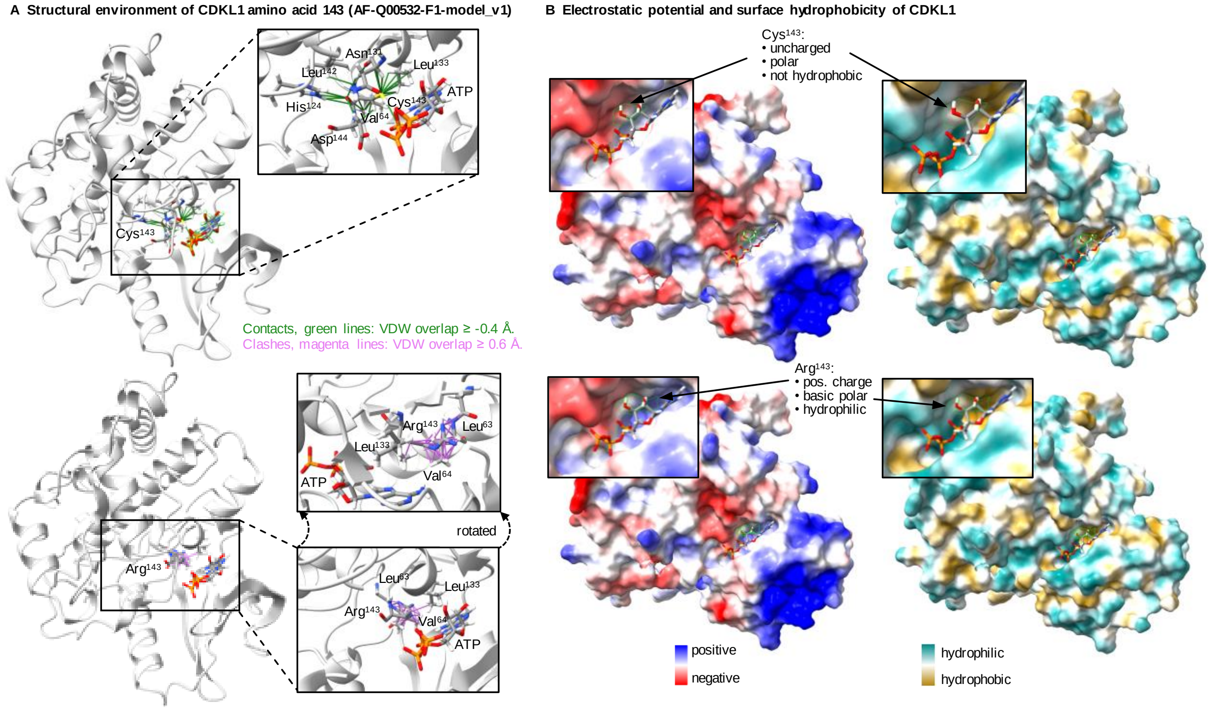

Supplemental Figure 2. *In silico* analysis of the CDKL1 p.Cys143Arg amino acid change A. Structural and physicochemical impact of the CDKL1 p.Cys143Arg change. Ribbon representations of CDKL1 amino acids 1-303 (AlphaFold AF-Q00532-F1-model_v4; AlphaFill AF-Q00532-F1-model_v1) show amino acid 143 and surrounding residues within a radius of 5 Å as sticks. The ATP nucleotide was transplanted to the AlphaFold model by using the AlphaFill algorithm. Sidechains are colored by element (hydrogen: white; carbon: grey; oxygen: red; nitrogen: blue; sulfur: yellow; phosphorus: orange). The upper model shows the structural environment of Cys^{143} with Van-der-Waals (VDW) overlaps ≥ -0.4 Å (contacts, green lines), whereas the lower model depicts the structural environment of Arg^{143} with VDW overlaps ≥ 0.6 Å (clashes, magenta lines). Parts of Supplemental Figure 2A are shown in Figure 2C of the main manuscript. **B. Visualization of surface hydrophobicity and electrostatic potential of CDKL1.** Amino acid 143 is labeled in each model. The upper and lower models show CDKL1 before and after molecular replacement of Cys^{143} by arginine, respectively. The enlarged sections show the nucleotide binding pocket with ATP. The left models depict the electrostatic potential of wild-type (p.Cys143) and altered (p.Arg143) CDKL1, the right models show the surface hydrophobicity of wild-type (p.Cys143) and altered (p.Arg143) CDKL1. Different colors indicate the hydrophobicity properties and electrostatic potential of amino acids. The most hydrophilic residues are in cyan and the most hydrophobic residues are in tan. The electrostatic potential ranges from negative (red) to positive (blue). Both, hydrophobicity and electrostatic potential were calculated by using the surface coloring feature of the UCSF ChimeraX tool (version 1.4).

Supplemental Figure 3

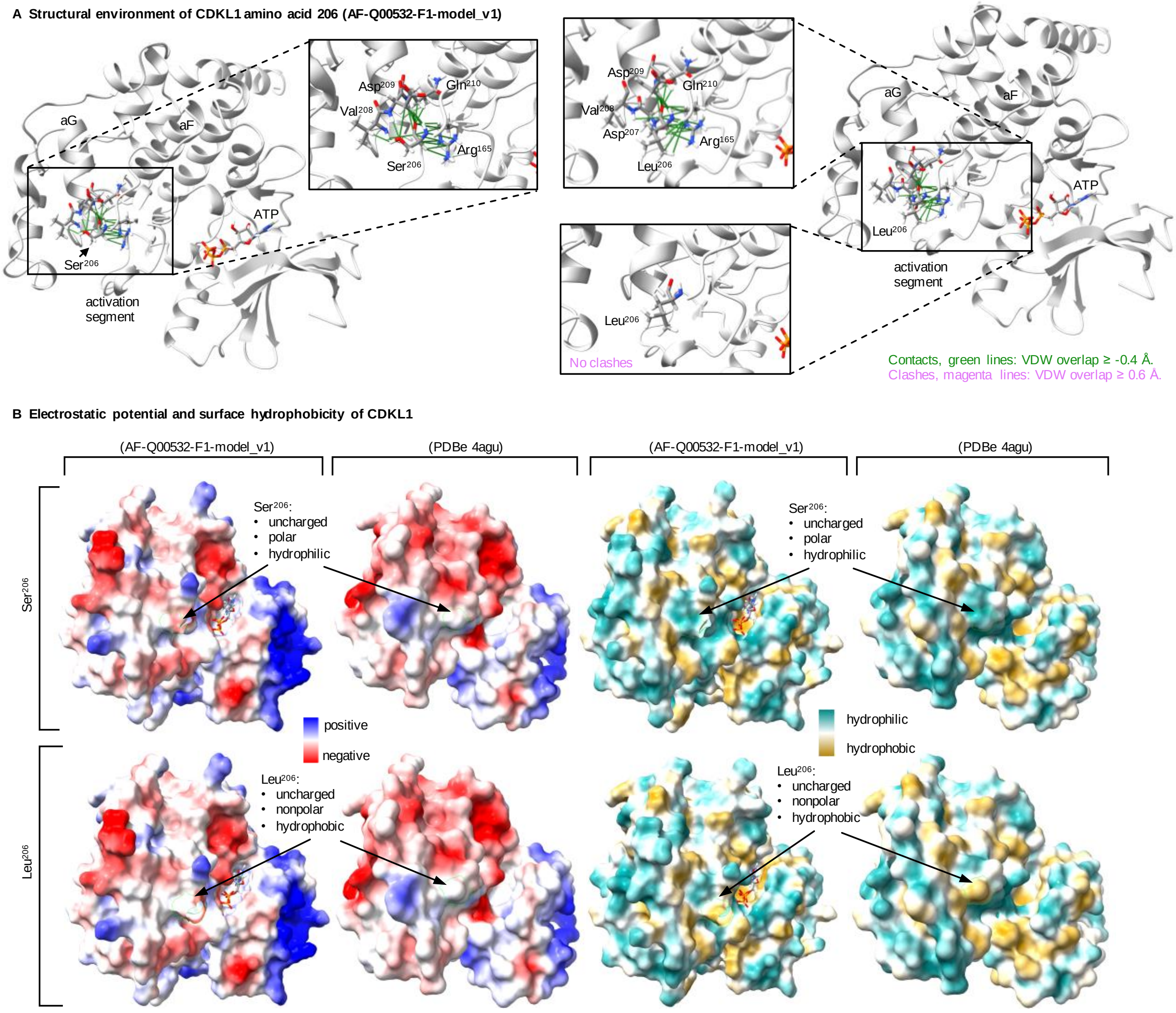

Supplemental Figure 3. *In silico* analysis of the CDKL1 p.Ser206Leu amino acid change A. Structural and physicochemical impact of the CDKL1 p.Ser206Leu amino acid change. Ribbon representations of CDKL1 amino acids 1-303 (AlphaFold AF-Q00532-F1-model_v4; AlphaFill AF-Q00532-F1-model_v1) show amino acid 206 and surrounding residues within a radius of 5 Å as sticks. The ATP nucleotide was transplanted to the AlphaFold model by using the AlphaFill algorithm. Sidechains are colored by element (hydrogen: white; carbon: grey; oxygen: red; nitrogen: blue; phosphorus: orange). The models shows the structural environment of Ser^{206} (left) or Leu^{206} (right) with Van-der-Waals (VDW) overlaps ≥ -0.4 Å (contacts, green lines). VDW overlaps ≥ 0.6 Å (clashes, magenta lines) were not identified. The secondary structure elements αF and αG helices are indicated and the activation segment is marked. **B. Visualization of electrostatic potential and surface hydrophobicity of CDKL1.** For modelling two alternative structures, AF-Q00532-F1-model_v1 and PDBe 4agu, were used. Amino acid 206 is labeled in each model. The upper and lower models show CDKL1 before and after molecular replacement of Ser^{206} by leucine, respectively. The left four models show the electrostatic potential of wild-type (p.Ser206, upper models) and altered (p.Leu206, lower models) CDKL1. The right four models depict the surface hydrophobicity of wild-type (p.Ser206, upper models) and altered (p.Leu206, lower models) CDKL1. Different colors indicate the electrostatic potential and the hydrophobicity of amino acids. The electrostatic potential ranges from negative (red) to positive (blue). The most hydrophilic residues are in cyan and the most hydrophobic residues are in tan. Both, electrostatic potential and hydrophobicity were calculated by using the surface coloring feature of the UCSF ChimeraX tool (version 1.4). Parts of Supplemental Figure 3B are shown in Figure 2C of the main manuscript.

Supplemental Figure 4

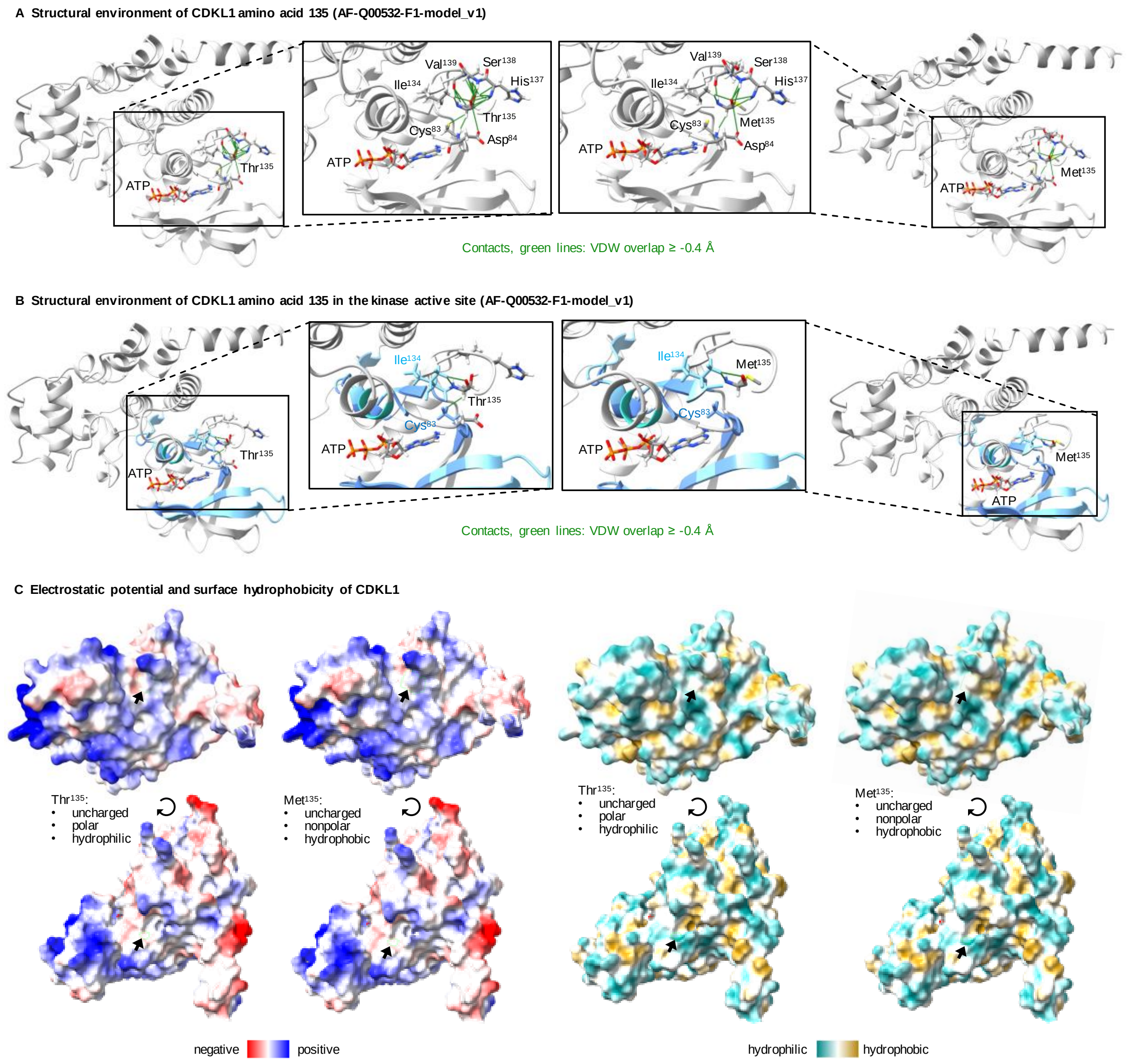

Supplemental Figure 4. ***In silico*** **analysis of the CDKL1 p.Thr135Met amino acid change. A. Structural and physicochemical impact of the CDKL1 p.Thr135Met alteration.** Ribbon representations of CDKL1 amino acids 1-303 (AlphaFold AF-Q00532-F1-model_v4; AlphaFill AF-Q00532-F1-model_v1) show amino acid 135 and surrounding residues within a radius of 5 Å as sticks. The ATP nucleotide was transplanted to the AlphaFold model by using the AlphaFill algorithm. Sidechains are colored by element (hydrogen: white; carbon: grey; oxygen: red; nitrogen: blue; sulfur: yellow; phosphorus: orange). The left and the right models show the structural environment of Thr^{135} and Met^{135} with Van-der-Waals (VDW) overlaps ≥ -0.4 Å (contacts, green lines). **B. Spatial proximity of amino acid 135 and a conserved kinase active site signature.** Different colors in the ribbon model indicate amino acids involved in ATP positioning (dark blue) and protein catalysis (light blue). Sidechains of amino acids Cys^{83}, Ile^{134}, Thr^{135}, and Met^{135} and ATP are colored by element (hydrogen: white; carbon: grey; oxygen: red; nitrogen: blue; sulfur: yellow; phosphorus: orange). Parts of Supplemental Figure 4B are shown in Figure 2C of the main manuscript. **C. Visualization of electrostatic potential and surface hydrophobicity of CDKL1.** Amino acid 135 is indicated in each model. The left and right models of each pair show CDKL1 before and after molecular replacement of Thr^{135} by methionine, respectively. The top and bottom models each show two different protein views. The four models on the left show the electrostatic potential of wild-type (p.Thr135) and altered (p.Met135) CDKL1; the four models on the

right depict the surface hydrophobicity of wild-type (p.Thr135) and altered (p.Met135) CDKL1. Different colors indicate the electrostatic potential and hydrophobicity properties of amino acids. The electrostatic potential ranges from negative (red) to positive (blue). The most hydrophilic residues are in cyan and the most hydrophobic residues are in tan. Both, electrostatic potential and hydrophobicity were calculated by using the surface coloring feature of the UCSF ChimeraX tool (version 1.4).

Supplemental Figure 5

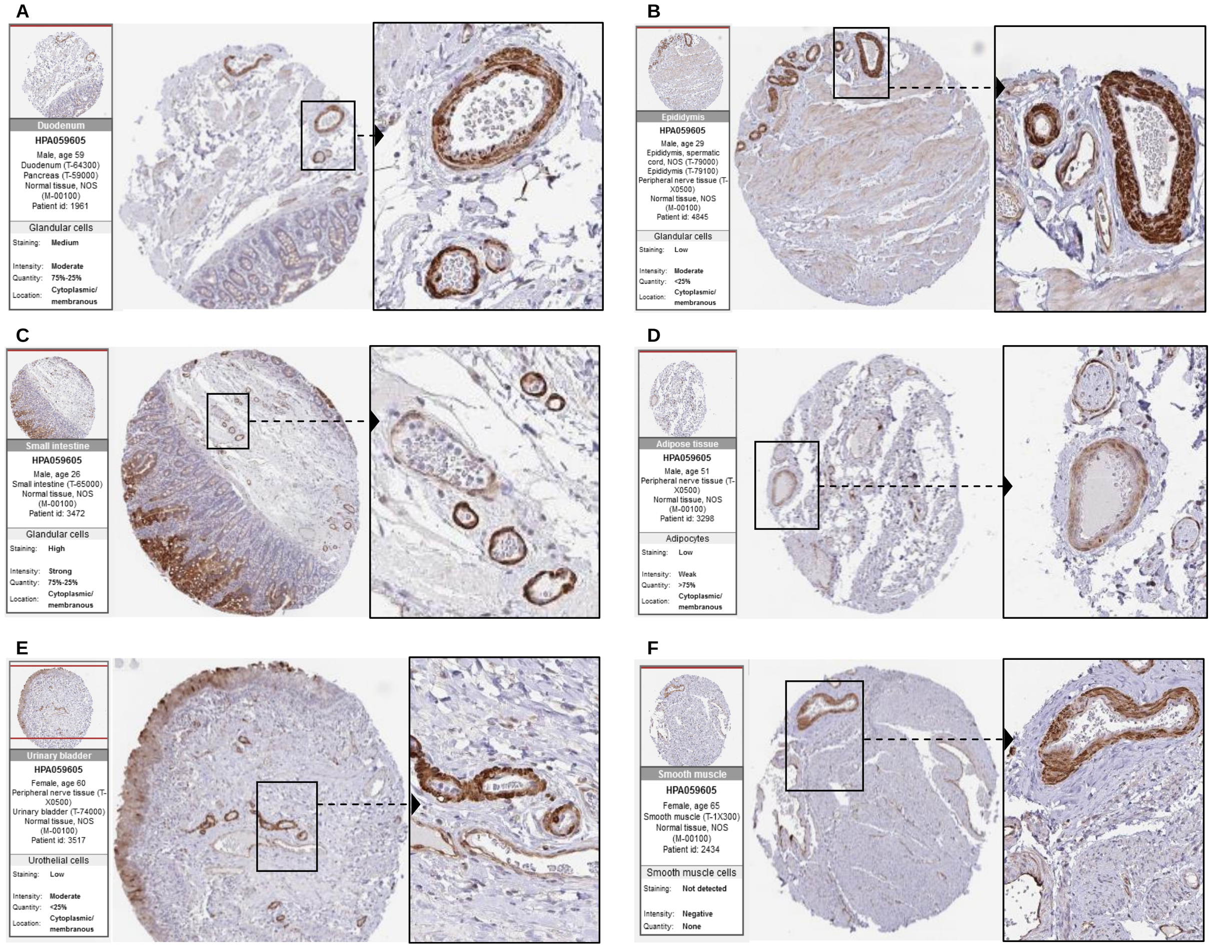

Supplemental Figure 5. CDKL1 is expressed in the wall of blood vessels. All images are from The Human Protein Atlas version 22 (v22.proteinatlas.org) (27). **A. Immunohistological staining of duodenum**. https://www.proteinatlas.org/ENSG00000100490-CDKL1/tissue/duodenum#img. **B. Immunohistological staining of epididymis**. https://www.proteinatlas.org/ENSG00000100490-CDKL1/tissue/epididymis#img. **C. Immunohistological staining of small intestine**. https://www.proteinatlas.org/ENSG00000100490-CDKL1/tissue/small+intestine#img. **D.** Immunohistological staining of adipose tissue; https://www.proteinatlas.org/ENSG00000100490-CDKL1/tissue/adipose+tissue#img. **E. Immunohistological staining of urinary bladder**. https://www.proteinatlas.org/ENSG00000100490-CDKL1/tissue/urinary+bladder#img. **F. Immunohistological staining of smooth muscle**. https://www.proteinatlas.org/ENSG00000100490-CDKL1/tissue/smooth+muscle#img.

Supplemental Figure 6

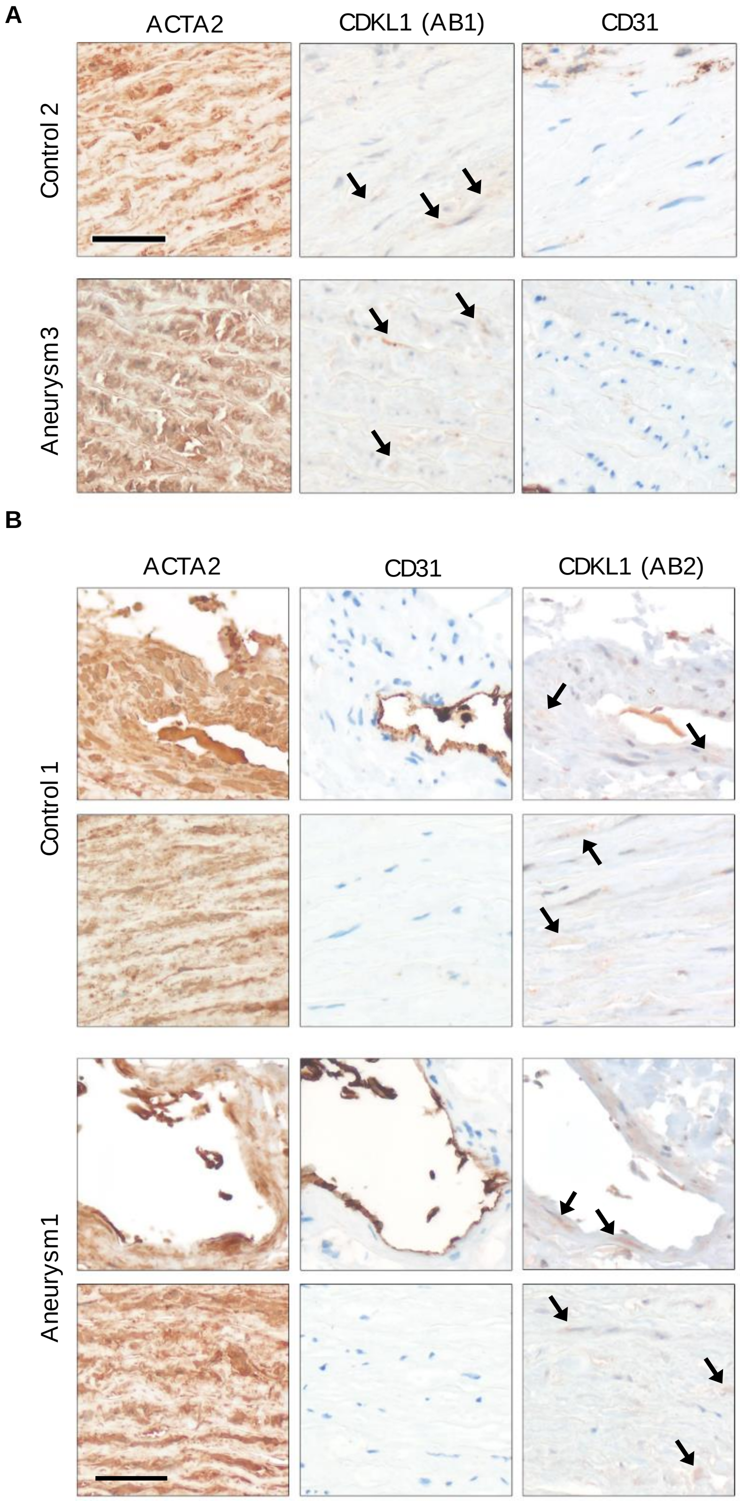

Supplemental Figure 6. CDKL1 IHC analyses in additional normal and diseased aortic tissue samples. A. Verification of CDKL1 expression in VSMCs in aorta tissue samples by using antibody 1 (AB1: Merck Sigma-Aldrich, HPA059605). CDKL1 co-localizes with ACTA2-positive cells in the media of control individual 2 (control 2) and a patient with aortic aneurysm (aneurysm 3). Smooth muscle actin (ACTA2), CDKL1 (AB1, 1:300), and the vascular marker CD31 were stained in consecutive sections of human aorta tissue. Representative pictures are shown. Scale bar: 50µm. **B. Verification of CDKL1 expression in VSMCs in aortic tissue samples by using antibody 2 (AB2: Thermo Fisher Scientific, PA5-101142).** CDKL1 co-localizes with ACTA2-positive cells in the media and in small vessels in the aortic wall of

control individual 1 (control 1) and a patient with aortic aneurysm (aneurysm 1). Smooth muscle actin (ACTA2), vascular marker CD31, and CDKL1 (AB2, 1:100) were stained in consecutive sections of human aorta tissue. Representative pictures are shown. ACTA2 and CD31 staining in Supplemental Figure 6B is also shown in Figure 3A of the main manuscript. Scale bar: 50µm.

Supplemental Figure 7

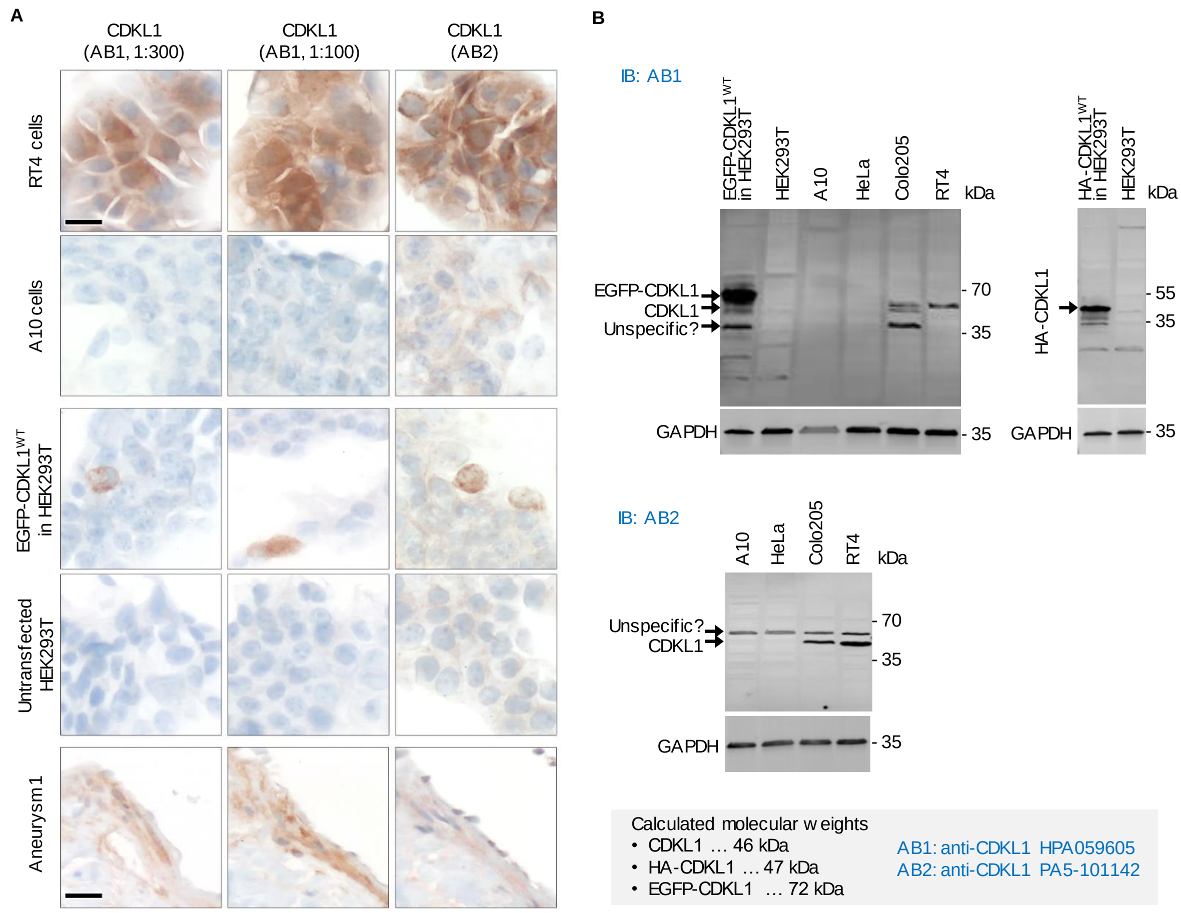

Supplemental Figure 7. Specificity validation of CDKL1 antibodies. A. Validation of two different CDKL1 antibodies on cell blocks and patient aorta tissue. Formalin-fixed paraffin-embedded cells were used to model paraffin-embedded human tissues. For this, RT4-, A10, HEK293T, and HEK293T cells transfected with EGFP-CDKL1 were fixed in formaldehyde, harvested by centrifugation, and embedded in paraffin similar to human tissue. Representative images of CDKL1-positive RT4-cells (upper panel) and CDKL1-negative A10-cells (second panel) are shown. HEK293T cells were transfected with EGFP-CDKL1 (third panel); untransfected HEK293T cells were used as negative control (forth panel). All samples were stained with CDKL1 antibodies [antibody 1 (AB1), Merck Sigma-Aldrich, HPA059605 at different dilutions and antibody 2 (AB2), Thermo Fisher Scientific, PA5-101142]. Both tested antibodies detected CDKL1 in RT4 cells and in CDKL1-transfected HEK293T cells. The signal-to-noise ratio was optimal for AB1 at 1:300 dilution. Scale bar cells: 20 µm. Human aortic tissue was stained in consecutive sections with both CDKL1 antibodies (lower panel). Representative staining showed that both antibodies detect CDKL1 in cells in the smooth muscle layer of a small vessel in the adventitia. Scale bar: 20 µm. **B. Immunoblotting analyses with CDKL1 antibodies in CDKL1-transfected HEK293T cells and various cell lines.** HEK293T cells were transfected with EGFP-CDKL1^{WT} (upper left western blot) or HA-CDKL1^{WT} (upper right western blot). Untransfected HEK293T, A10, HeLa, Colo205, and RT4, cells were cultivated under basal growth conditions. After cell lysis, samples were subjected to immunoblotting as indicated. Two different antibodies were used for the detection of CDKL1: Merck Sigma-Aldrich, HPA059605 (AB1) and Thermo Fisher Scientific, PA5-101142 (AB2). GAPDH was used as loading control. Suspected specific and unspecific bands are marked.

Supplemental Figure 8

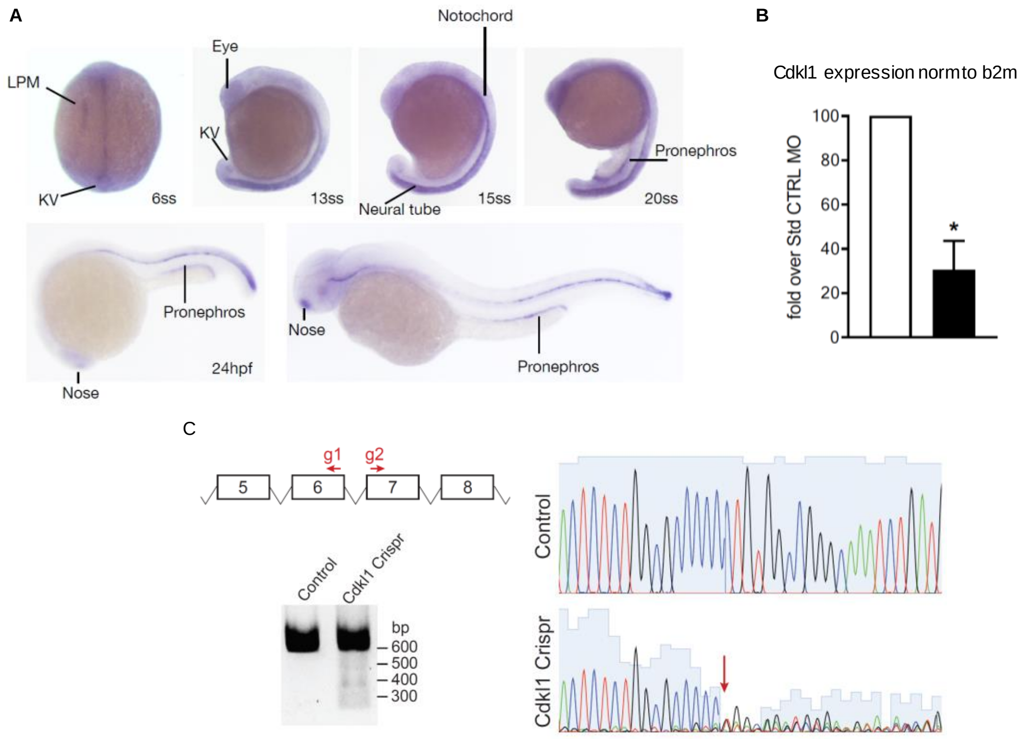

Supplemental Figure 8. Zebrafish experiments. A. Temporal and spatial expression of *cdkl1* in *D. rerio*. Expression of *cdkl1* was analyzed by whole mount in situ hybridization. The respective developmental stage is given in the lower right. Various organs are indicated. LPM, lateral plate mesoderm; KV, Kupffer's vesicle; Notochord, chorda dorsalis; ss, somite state; hpf, hours post fertilization. **B. Verification of splice blocking efficiency of cdkl1 splMO.** RNA was isolated from 24 hpf embryos injected with standard control morpholino oligonucleotides (Std CTRL MO) or Cdkl1 splice site morpholino oligonucleotides (Cdkl1 splMO) and transcribed into cDNA. *cdkl1* content was analysed by qPCR and normalized to *b2m* (beta-2-microglobulin). qPCR raw data are available on request. Data were plotted as mean values ± SEM and analyzed using Students t-test; *, $P \leq 0.01$. **C. Crispr/Cas9-mediated Cdkl1 gene editing.** The cartoon shows exons 5-8 of zebrafish *cdkl1* (ENSDART00000044357) with the position and direction of the guideRNAs (g1, g2). The example agarose gel picture of PCR shows that *Cdkl1* RNP injection gives rise to several bands; sequencing of the smaller bands revealed deletions ranging from 148-339 bp between the sequences targeted by the two guide RNAs. The example sequencing of the band at 610 bp shows that the sequence is interrupted at the position where gRNA1 would start to align (arrow).

Supplemental Figure 9

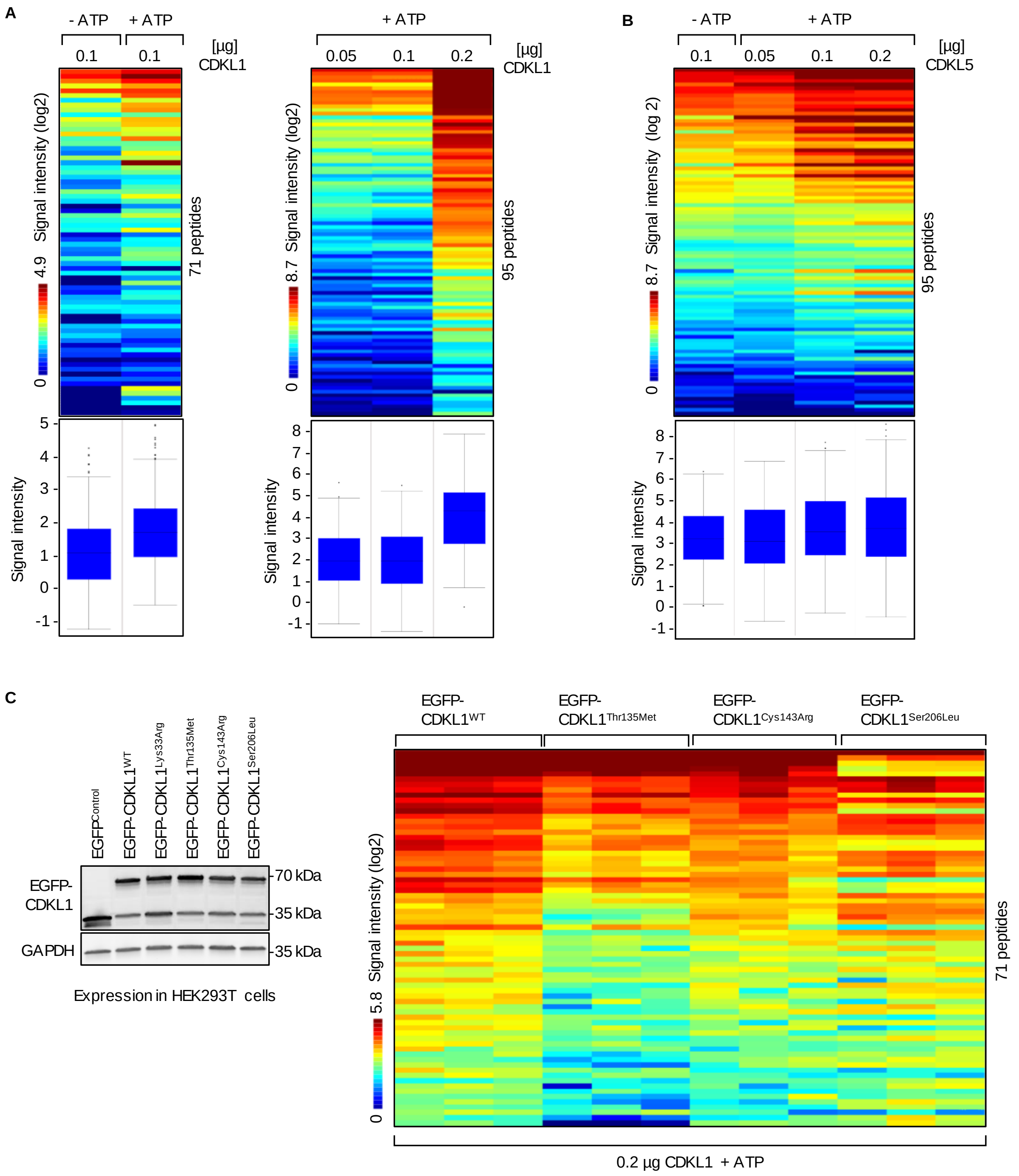

Supplemental Figure 9. CDKL1 kinase activity profiling. A. Recombinant CDKL1 was purchased (Signal Chem Biotech.) and - in the respectively specified amount (0.05, 0.1, 0.2 µg) - applied to STK-PamChip arrays in absence or presence of ATP. 71 (left heatmap) and 95 (right heatmap) peptides passed quality control. The left and right heatmaps demonstrate that phosphorylation of substrate peptides depend on ATP and CDKL1 concentration, respectively. The signals were sorted from high (red) to low (blue) intensity (upper panel each) that corresponds to phosphorylation level. To visualize overall sample variance and group differences, peptide phosphorylation is shown in box plot representations (lower panel each). **B.** For comparison and to demonstrate functionality of functional kinome profiling, we analyzed the well-described CDKL5 kinase (172). Recombinant CDKL5 was purchased (Signal Chem Biotech.) and - in the respectively specified amount (0.05, 0.1, 0.2 µg) - applied to reaction mix. 95

peptides passed quality control. The heatmap demonstrates that phosphorylation of substrate peptides depend on ATP and CDKL5 concentration. Signals were sorted from high (red) to low (blue) intensity (upper panel each) that corresponds to phosphorylation level. To visualize overall sample variance and group differences, peptide phosphorylation is shown in box plot representations (lower panel). **C**. EGFP-tagged CDKL1 protein variants were expressed in HEK293T cells. Small aliquots were removed from cell lysates and subjected to immunoblotting using anti-EGFP and anti-GAPDH antibodies. EGFP-CDKL1 protein variants were purified by using GFP-Trap and 0.2 µg purified protein and ATP were applied to STK-PamChip arrays. 71 peptides passed quality control. The heatmap displays the log2-transformed signal intensities for the indicated CDKL1 variants with 3 replicates per variant. The signals were sorted from high (red) to low (blue) intensity/phosphorylation.

Supplemental Figure 10

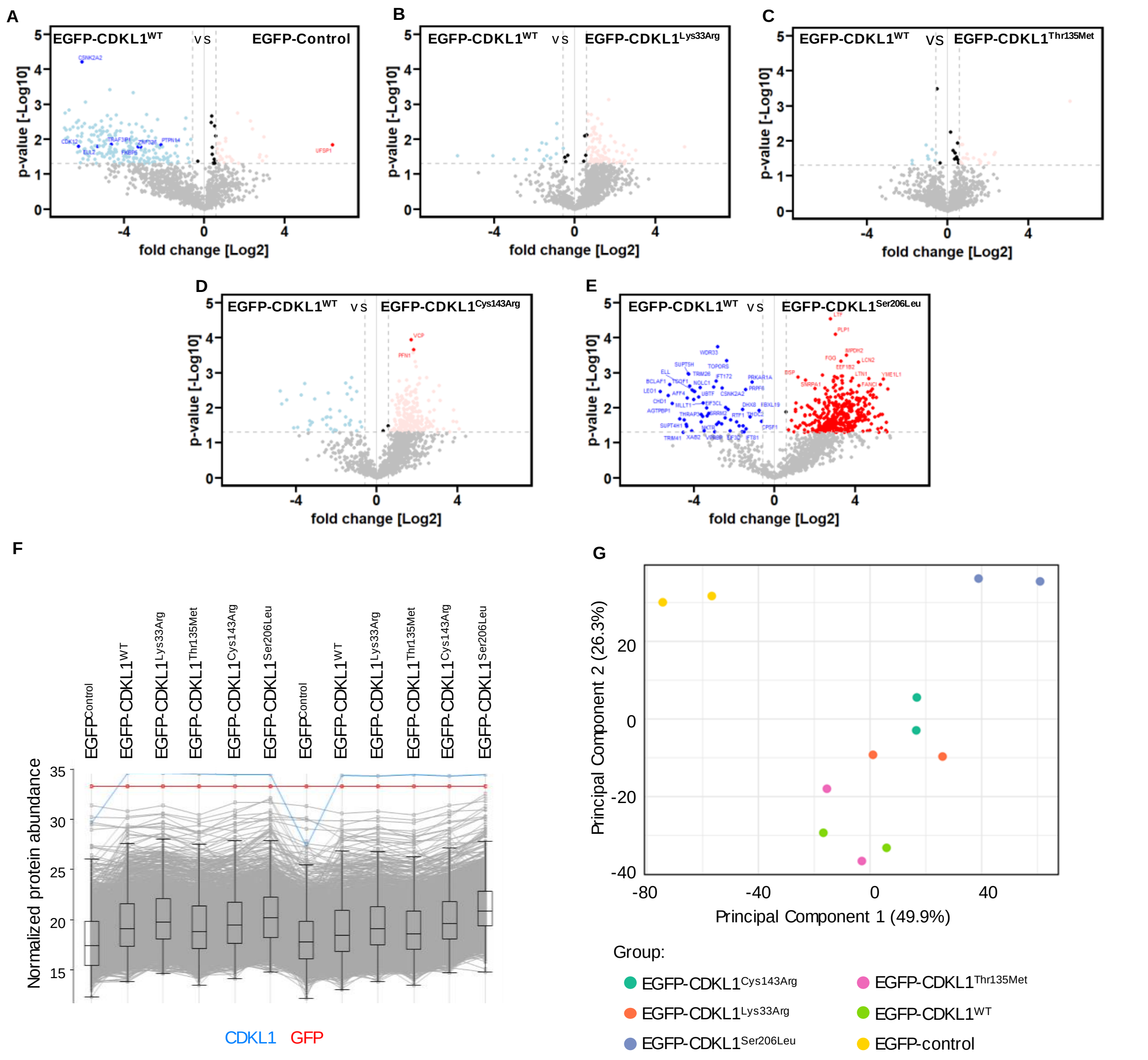

Supplemental Figure 10. Differential abundant proteins in CDKL1 precipitates. (**A-E**) EGFP-tagged CDKL1 protein variants were expressed in HEK293T cells, purified by using GFP-Trap and subjected to LCMS/MS analysis. Volcano plot visualization of t-testing results between indicated pairs of CDKL1 variants including (**A**) empty vector control (EGFP control) vs CDKL1^{WT}, (**B**) CDKL1^{Lys33Arg} vs CDKL1^{WT}, (**C**) CDKL1^{WT} vs CDKL1^{Thr135Met}, (**D**) CDKL1^{WT} vs CDKL1^{Cys143Arg}, and (**E**) CDKL1^{WT} vs CDKL1^{Ser206Leu}. Proteins were considered significantly differential abundant, if they exceeded a q-value cutoff < 0.05 (permutation-based FDR), as well as a mean fold difference (FC, fold change) > 1.5-fold. Q-value significant proteins are labelled. Proteins, which are only p-value significant (cut-off < 0.05), are shown as unlabelled dots and slightly paler in colour. **(F)** Profile Plot showing all protein abundances normalized to green fluorescent protein (GFP) over all samples. GFP is highlighted in red and CDKL1 in blue. **(G)** Scatter Plot of the first two principal components according to principal component analysis (PCA), based on 1,304 proteins.

Supplemental Figure 11

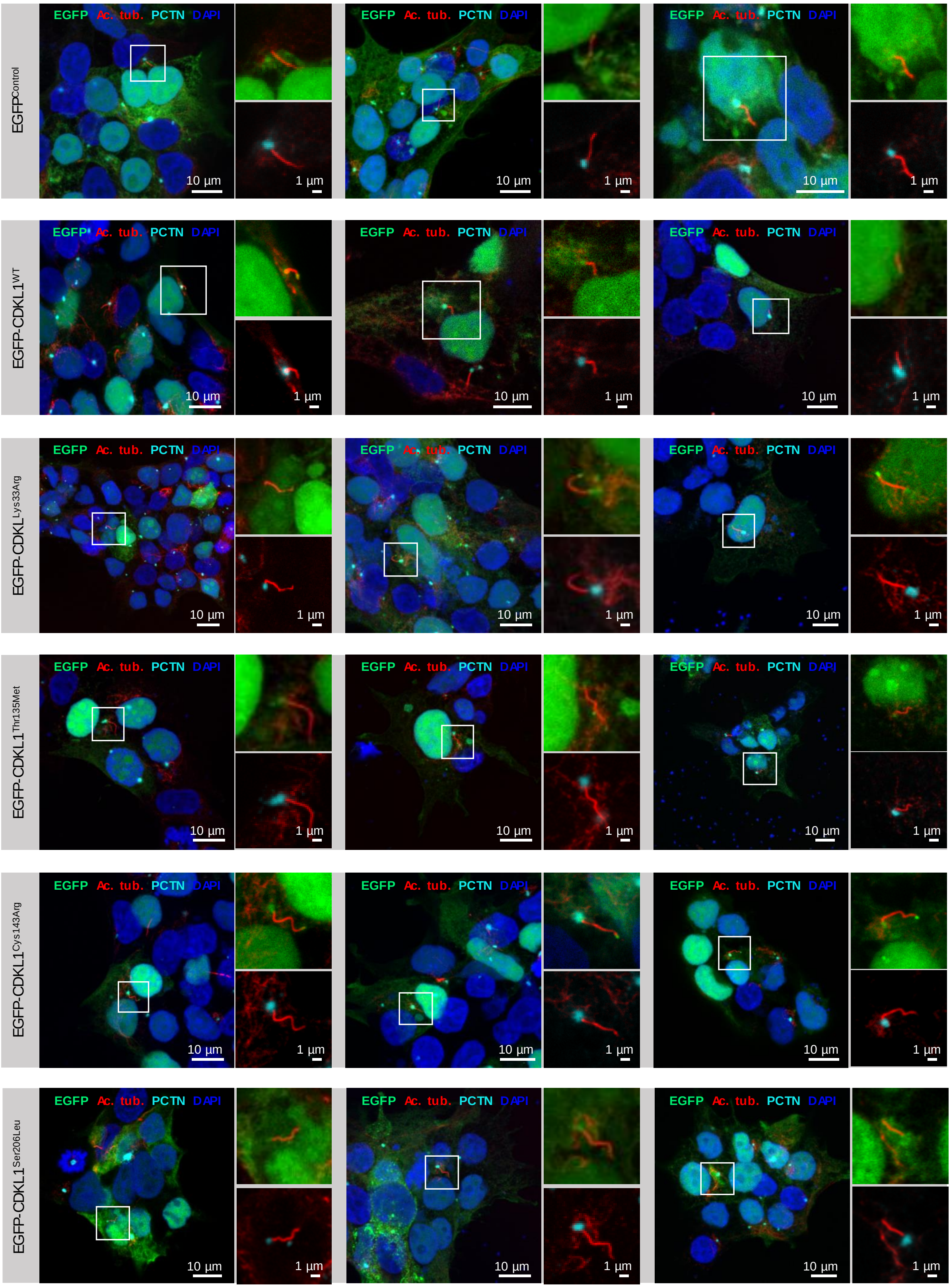

Supplemental Figure 11. Analysis of the subcellular localization of CDKL1 variants and their influence on primary cilia morphology. EGFP-CDKL1 variants (green) were transiently expressed in HEK293T cells. After fixation, cells were stained for the axoneme of primary cilia with mouse monoclonal antibodies against acetylated tubulin (red) and for the basal body with rabbit polyclonal antibodies against pericentrin (cyan). Nuclei were stained with DAPI (blue) and images were acquired at a confocal microscope. Shown are representative images of CDKL1-expressing cells with primary cilia. Six images

(one for each condition) inclusively respective close ups in Supplemental Figure 11 are also shown in Figure 7C of the main manuscript. Scale bar 10 µm and 1 µm in close up images.

Supplemental Figure 12

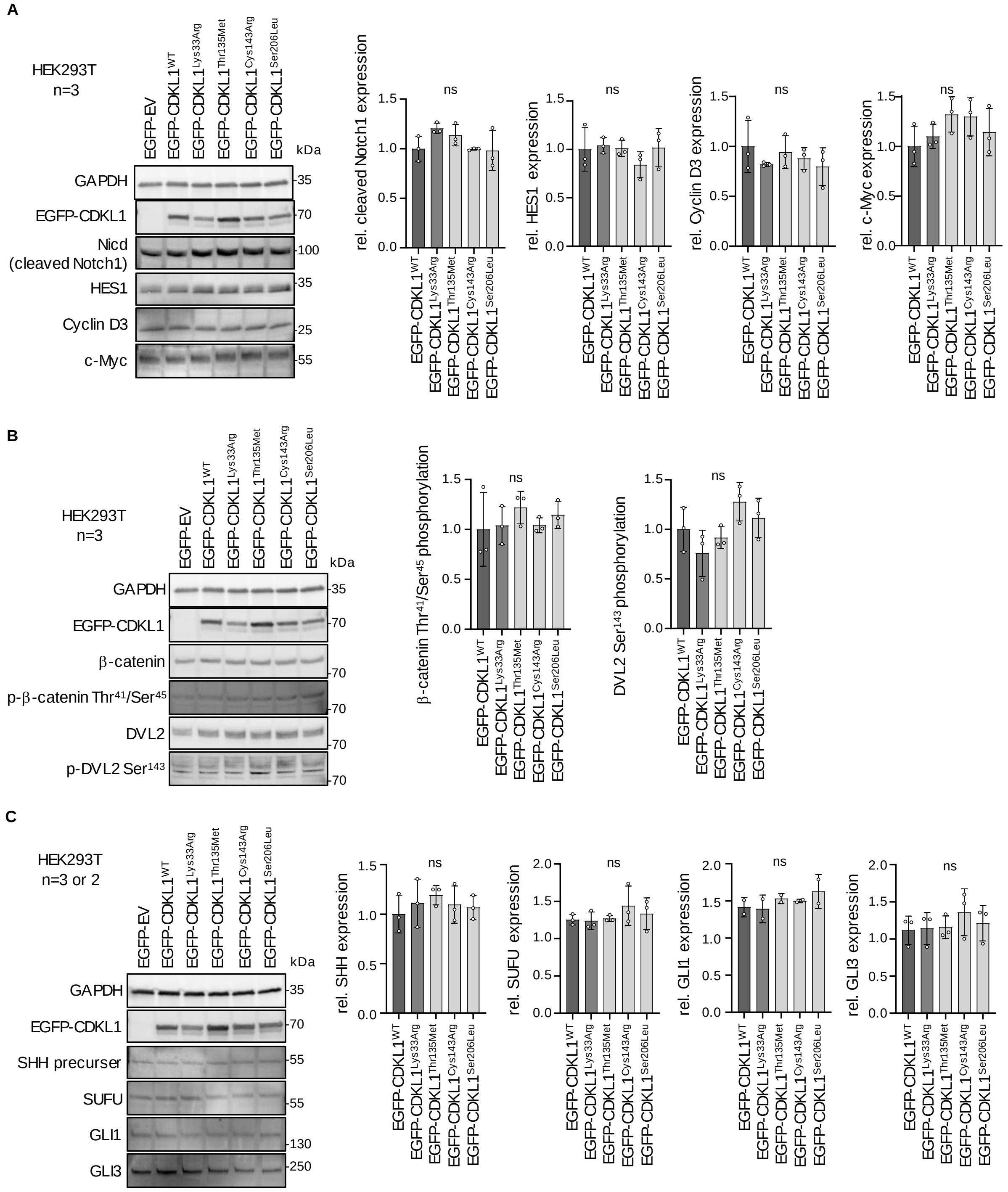

Supplemental Figure 12. Ciliary pathways are not altered by expression of CDKL1 variants. Total lysates of HEK293T cells transiently expressing $CDKL1^{WT}$, $CDKL1^{Thr135Met}$, $CDKL1^{Cys143Arg}$, or $CDKL1^{Ser206Leu}$ were subjected to immunoblotting as indicated. Cells expressing kinase-dead $CDKL1^{Lys33Arg}$ and empty vector (EV) transfected cells were used as controls. GAPDH was used as loading control. The mean of n independent experiments ± SD is given. One-way ANOVA with Tukey's multiple comparison test was used. ns, not significant. **A. NOTCH1 signaling pathway is not affected by the expression of CDKL1 variants.** The graphs show protein levels of cleaved Notch1 (Nicd) and its targets HES1, Cyclin D3, and c-Myc from three independent experiments (n=3), each normalized to GAPDH. Protein levels in cells expressing CDKL1 mutants are relative to those in $CDKL1^{WT}$ cells. **B CDKL1 variants do not affect Wnt signaling.** The graphs show levels of phosphorylated β-catenin (at Thr^{41} and Ser^{45}) and of phosphorylated Dvl2 (at Ser^{143}) from three independent experiments (n=3), each normalized to total

β-catenin and total Dvl2, respectively, as well as to GAPDH. Phosphorylation levels in cells expressing CDKL1 mutants are relative to those in CDKL1^{WT} cells. GAPDH and EGFP-CDKL1 blots in Supplemental Figures 12A and 12B are identical because they derive from the same experiments. **C. Expression of CDKL1 variants has no effect on SHH-mediated signaling.** The graphs show protein levels of SHH and its targets SUFU, GLI1, and GLI3 from three (SHH, SUFU, GLI3) or two (GLI1) independent experiments (n=3 or 2), each normalized to GAPDH. Protein levels in cells expressing CDKL1 mutants are relative to those in CDKL1^{WT} cells.

Supplemental Figure 13

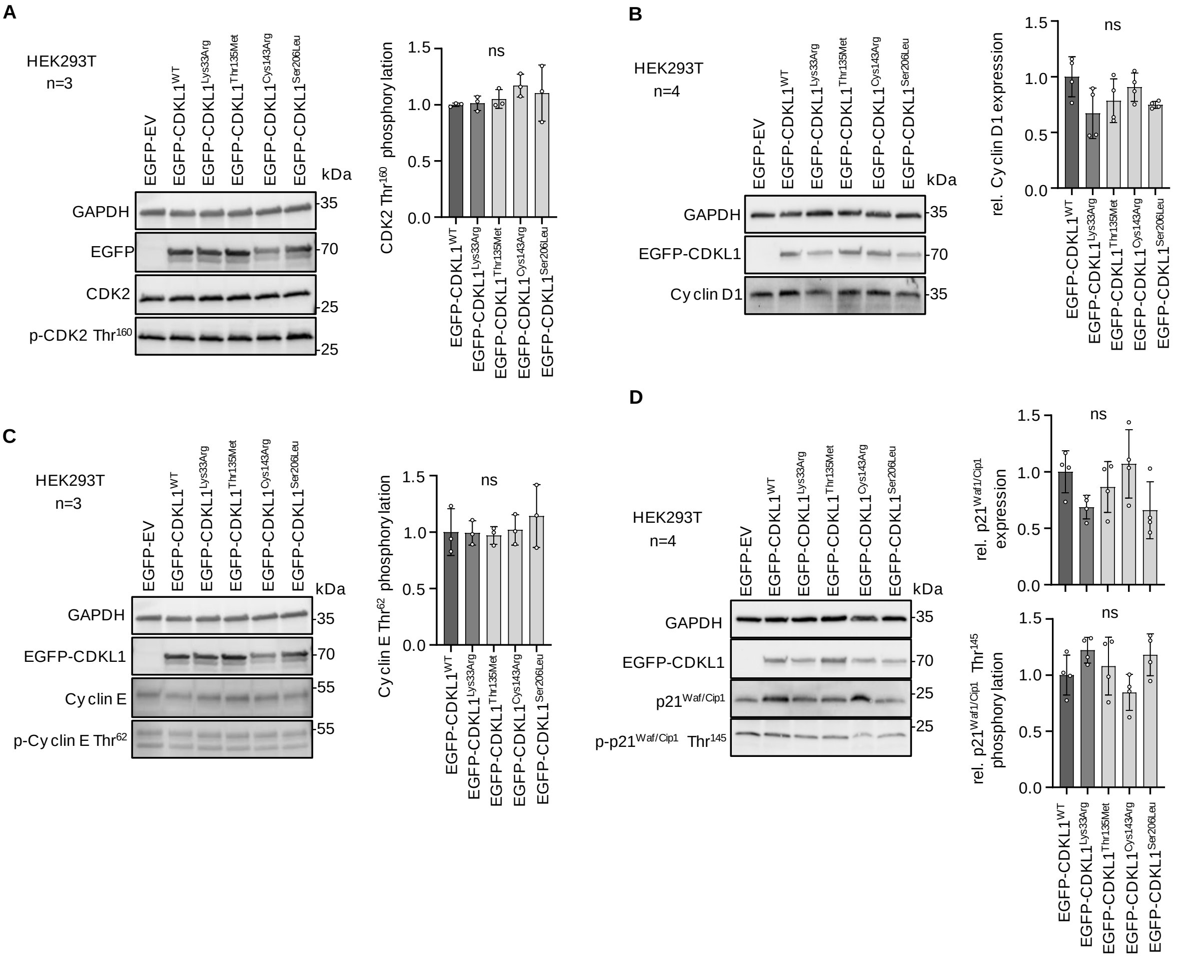

Supplemental Figure 13. Expression of CDKL1 variants does not interfere with expression and/or phosphorylation of cell cycle regulators. Total lysates of HEK293T cells transiently expressing CDKL1^{WT}, CDKL1^{Thr135Met}, CDKL1^{Cys143Arg}, or CDKL1^{Ser206Leu} were subjected to immunoblotting as indicated. Cells expressing kinase-dead CDKL1^{Lys33Arg} and empty vector (EV) transfected cells were used as controls. GAPDH was used as loading control. The mean of n independent experiments ± SD is given. One-way ANOVA with Tukey's multiple comparison test was used. ns, not significant. **A. Expression and phosphorylation of CDK2 is not affected in cells expressing CDKL1 variants.** The graph shows phosphorylation of CDK2 (at Thr^{160}) normalized to amounts of total CDK2 and GAPDH from four independent experiments (n=3). Phosphorylation levels in cells expressing CDKL1 mutants are relative to those in CDKL1^{WT} cells. **B. Expression of Cyclin D1 is not affected in cells expressing CDKL1 variants.** The graph shows expression of Cyclin D1 normalized to GAPDH relatively to CDKL1^{WT} from four independent experiments (n=4). Expression levels in cells expressing CDKL1 mutants are relative to those in CDKL1^{WT} cells. **C. Phosphorylation of Cyclin E is unaltered in cells expressing CDKL1 variants.** The graph shows relative phosphorylation of Cyclin E (at Thr^{62}) normalized to amounts of total Cyclin E and GAPDH from three independent experiments (n=3). Phosphorylation levels in cells expressing CDKL1 mutants are relative to those in CDKL1^{WT} cells. GAPDH and EGFP-CDKL1 blots in Supplemental Figures 13A and 13C are identical because they derive from the same experiments. **D. Consequences of CDKL1 variants on expression and phosphorylation of p21$^{WAF1/CIP1}$.** Graphs show expression and phosphorylaton of p21 in cells transfected with CDKL1 mutants relatively to CDKL^{WT}-transfected cells from four independent experiments (n=4). p21 expression was normalized to GAPDH and phospho-p21 (at Thr^{145}) was normalized to amounts of total p21 and GAPDH.

Supplemental Figure 14

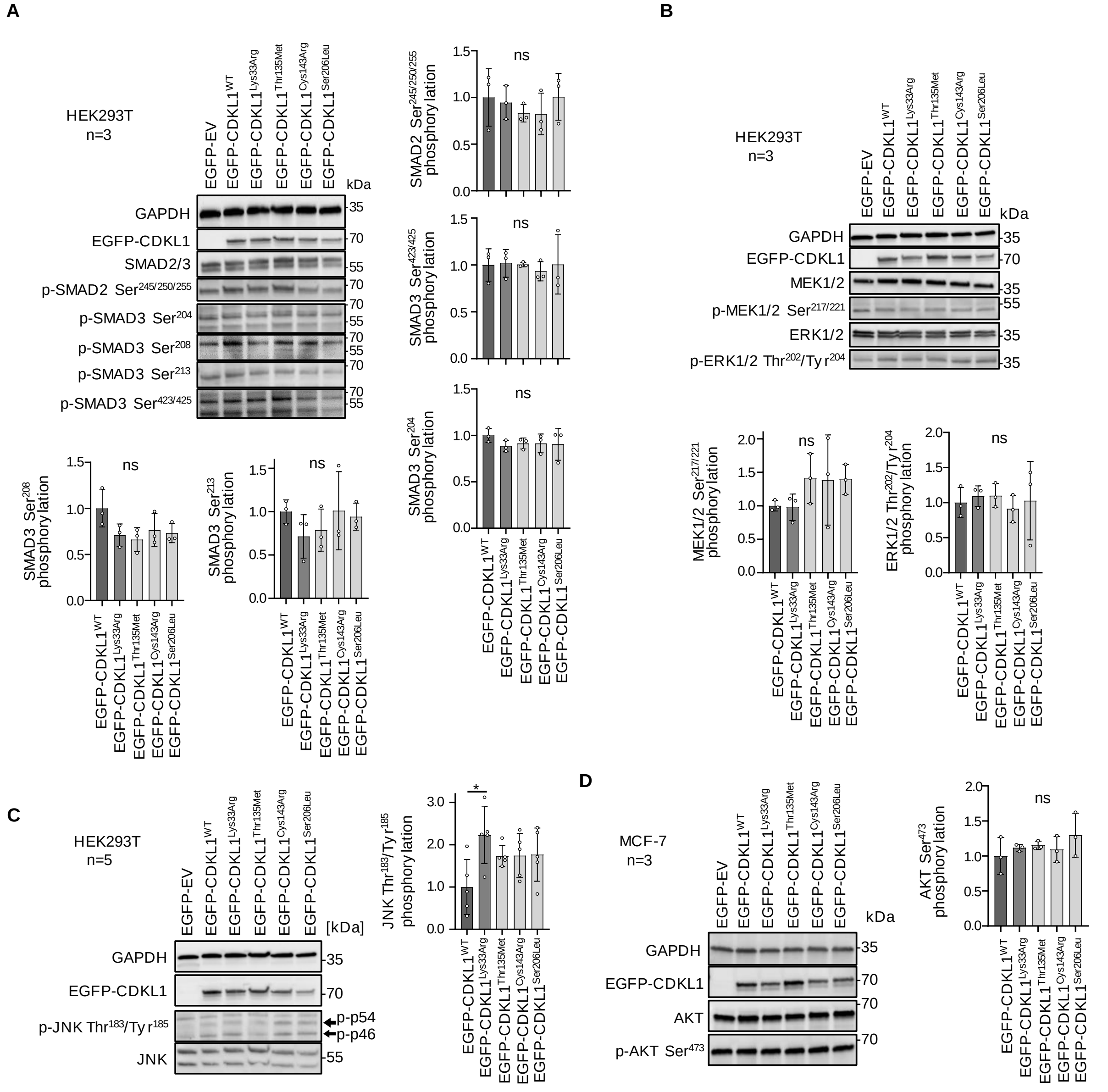

Supplemental Figure 14. Disease-associated CDKL1 variants do not significantly interfere with signaling via SMAD2/3, MEK1/2-ERK1/2, SAPK/JNK, and AKT. Total lysates of HEK293T (**A, B, and C**) or MCF-7 (**D**) cells transiently expressing CDKL1^{WT}, CDKL1^{Thr135Met}, CDKL1^{Cys143Arg}, or CDKL1^{Ser206Leu} were subjected to immunoblotting as indicated. Cells expressing kinase-dead CDKL1^{Lys33Arg} and empty vector (EV) transfected cells were used as controls. GAPDH was used as loading control. The mean of n independent experiments ± SD is given. One-way ANOVA with Tukey's multiple comparison test was used. ns, not significant; *, $P \leq 0.05$. **A**. **Phosphorylation of SMAD2 and SMAD3 is not significantly affected in cells expressing CDKL1 variants.** Graphs show phosphorylation (±SD) of SMAD2 (at Ser$^{245/250/255}$) and SMAD3 (at Ser^{204}, Ser^{208}, Ser^{213}, or Ser$^{423/425}$) each normalized to amounts of total SMAD2/3 and GAPDH and relatively to CDKL1^{WT} from three independent experiments (n=3). **B**. **Phosphorylation of MEK1/2 and ERK1/2 is not significantly affected in cells expressing CDKL1 variants.** Graphs show relative phosphorylation (±SD) of MEK1/2 (at Ser$^{217/221}$) and ERK1/2 (at Thr^{202}/Tyr^{204}) normalized to amounts of total MEK1/2 and ERK1/2, respectively, as well as to GAPDH from three independent experiments (n=3). Phosphorylation levels in cells expressing CDKL1 mutants are relative to those in CDKL1^{WT} cells. Note, phospho-MEK1/2 Ser$^{217/221}$ antibody detects MEK1 Ser$^{218/222}$ and MEK Ser$^{222/226}$; phospho-ERK1/2 Thr^{202}/Tyr^{204} detects ERK1 Thr^{202}/Tyr^{204} and ERK2

Thr^{185}/Tyr^{187}. **C. Phosphorylation of JNK is not significantly affected in cells expressing disease-associated CDKL1 variants.** The graph shows means (±SD) of the relative phosphorylation of p54 and p46 JNK isoforms (at Thr^{183} and Tyr^{185}) normalized to amounts of total JNK as well as to GAPDH from five independent experiments (n=5). Phosphorylation levels in cells expressing CDKL1 mutants are relative to those in $CDKL1^{WT}$ cells. **D. AKT1/2/3 phosphorylation is not affected by the expression of $CDKL1^{Thr135Met}$, $CDKL1^{Cys143Arg}$, or $CDKL1^{Ser206Leu}$.** Graphs show phosphorylation (±SD) of AKT1/2/3 at $Ser^{473/474/472}$ normalized relative to amounts of total AKT1/2/3 and GAPDH and relatively to $CDKL1^{WT}$ from three independent experiments (n=3).

SUPPLEMENTAL TABLES

Supplemental Table 1. Known disease genes and risk genes for vascular/connective tissue disorders (based on and adapted from Demal TJ *et al*. Expanding the clinical spectrum of COL2A1 related disorders by a mass like phenotype. Sci Rep. 2022 Mar 16;12(1):4489) (173).

Gene	NM-Nummer	Disease (Inheritance)	Prevalence[A]<br>Penetrance[B]	Maximum genetic contribution (genetic heterogeneity)[C]	Maximum allelic contribution (allelic heterogeneity)[D]	Maximal Population Frequency (MPF)	References and databases
ABL1	NM_007313.3	Congenital heart defects and skeletal malformations syndrome (AD)	unreported (est. 1:1,000,000)<br>100%	100%	40%	2.00E-07	(174, 175); OMIM #617602
ACTA2	NM_001613.2	Aortic aneurysm, familial thoracic 6 (AD)	1:1,000<br>50%	12-21% (20%)	8%	1.60E-05	(40, 176); LOVD
		Multisystemic smooth muscle dysfunction syndrome (AD)	1:1,000,000<br>unreported (est. 50%)	unreported (100%[†])	8%	8.00E-08	ORPHA:91387; LOVD
ADAMTS10	NM_030957.3	Weill-Marchesani syndrome 1, recessive (AR)	1:100,000<br>100%	35%	10%[§]	1.87E-04	(177)
ADAMTS2	NM_014244.4	Ehlers-Danlos syndrome, dermatosparaxis type (AR)	est. 1:1,000,000<br>unreported (est. 50%)	unreported (100%[†])	33,3%	4.67E-04	OMIM #225410, *604539; ORPHA:1901; ClinVar; LOVD
ARIH1	NM_005744.5	Aortic aneurysm, familial thoracic N.S. (AD)	1:1,000<br>100%	1%	33%	1.65E-06	(178)
B3GALT6	NM_080605.3	Ehlers-Danlos syndrome spondylodysplastic type 2 (AR)	unreported (est. 1:1,000,000)<br>unreported (est. 50%)	only 16 cases known (100%[†])	9%	1.27E-04	(179); OMIM *615291, #615349; LOVD
B4GALT7	NM_007255.2	Ehlers-Danlos syndrome, spondylodysplastic type, 1 (AR)	unreported (est. 1:1,000,000)<br>unreported (est. 50%)	unreported (100%[†])	50%	7.07E-04	(179); OMIM *604327, #130070; LOVD
BGN	NM_001711.5	Aortic aneurysm, familial thoracic / Meester-Loeys syndrome (XL)	1:1,000<br>50%	rare (1%)	100%	1.00E-05[E]	(40, 176); OMIM *301870, #300989
CBS	NM_000071.2	Homocystinuria, B6-responsive and nonresponsive types (AR)	1:200,000-1:335,000<br>unreported (est. 50%)	100%	50%	1.58E-03	(180); ORPHA:394
CHST14	NM_130468.3	Ehlers-Danlos syndrome, musculocontractural type 1 (AR)	unreported (est. 1:1,000,000)<br>unreported (est. 50%)	unreported (100%[†])	25%	3.54E-04	(181, 182); OMIM #601776, *608429; ORPHA:2953; LOVD
COL1A1	NM_000088.3	Ehlers-Danlos syndrome, classic type (AD)	1:20,000<br>unreported (est. 50%)	1%	50%	2.50E-07	(179, 183)
		Ehlers-Danlos syndrome, arthrochalasia type 1 (AD)	unreported (est. 1:1,000,000)<br>unreported (est. 50%)	unreported (100%[†])	10%[§]	1.00E-07	(179); ORPHA:1899; LOVD; OMIM #130060, *120150
COL1A2	NM_000089.3	Ehlers-Danlos syndrome, cardiac valvular type, 2 (AR)	1:1,000,000<br>unreported (est. 50%)	unreported (100%[†])	2%-10%	0.000141	(179); ORPHA:230851; LOVD, OMIM #225320, *120160; LOVD
		Ehlers-Danlos syndrome, arthrochalasia type, 2 (AD)	unreported (est. 1:1,000,000)<br>unreported (est. 50%)	unreported (100%[†])	4%-10%	1.00E-07	(179); ORPHA:1899; LOVD; OMIM 617821, *120160; LOVD
COL2A1	NM_001844.4	Stickler syndrome, type I (AD)	1:7,500-1:9,000<br>100%	80-90%	2%-10%	5.15E-06	(184-188); LOVD
		Czech dysplasia (AD)	1:1,000,000<br>100%	100%	100%	5.00E-07	OMIM #609162, *120140

COL3A1	NM_000090.3	Aortic aneurysm, familial thoracic N.S. (AD)	1:1,000<br>50%	rare (1%)	100%	1.00E-05	(40, 176)
		Ehlers-Danlos syndrome, vascular type (AD)	1:50,000<br>100%	95%	4%	3.80E-07	(189); LOVD
COL4A1	NM_001845.5	Angiopathy, hereditary, with nephropathy, aneurysms, and muscle cramps (AD)	fewer than 100 families (est. 1:1,000,000)<br>100%	100%	7.5%	4.00E-08	(190); LOVD
COL4A5	NM_000495.4	Alport syndrome 1, X-linked (XLD)	1:50,000<br>unreported (est. 50%)	85%	10%	1.70E-06[E]	(191); LOVD
COL5A1	NM_000093.4	Ehlers-Danlos syndrome, classic type (AD)	1:20,000<br>unreported (est. 50%)	78%	5%	1.95E-06	(183); LOVD
COL5A2	NM_000393.4	Ehlers-Danlos syndrome, classic type (AD)	1:20,000<br>unreported (est. 50%)	14%	10%	3.50E-07	(183); LOVD
DCHS1	NM_003737.3	Mitral valve prolapse 2 (AD)	1:40<br>unreported (est. 50%)	25%	10%[§]	6.25E-04	(192); OMIM #607829, *603057
EFEMP2	NM_016938.4	Cutis laxa, type IB (AR)	1:4,000,000<br>incomplete (est. 50%)	100%	25%	1.77E-04	(193); ClinVar
ELN	NM_001278939.1	Cutis laxa (AD)	rare (1:1,000,000)<br>incomplete (est. 50%)	unreported (100%[†])	10%[§]	1.00E-07	(194); OMIM #123700, *130160
EMILIN1	NM_007046.3	Aortic aneurysm, familial thoracic N.S. (AD)	1:1,000<br>50%	<1%<br>(in one family)	100%	1.00E-05	(40, 176, 195)
EP300	NM_001429.4	Rubinstein-Taybi syndrome 2 (AD)	1:100,000-125,000<br>100%	8%-10%	3%	1.35e-08	(196, 197); #613684; LOVD
FBLN5	NM_006329.3	Cutis laxa, autosomal recessive, type IA (AR)	Rare (1:1,000,000)<br>incomplete (est. 50%)	100%	10%[§]	1.41E-03	(198)
FBN1	NM_000138.4	Marfan syndrome (AD)	1:7,500<br>100%	>95%	3%	2.00E-06	(199, 200); LOVD
		Aortic aneurysm, familial thoracic N.S. (AD)	1:1,000<br>50%	3%	100%	3.00E-05	(40, 176)
FBN2	NM_001999.3	Contractural arachnodactyly, congenital (AD)	unreported (est. 1:20,000)<br>100%	25%-75	9.7%	2.25E-06	(201, 202)
FKBP14	NM_017946.3	Ehlers-Danlos syndrome, kyphoscoliotic type, 2 (AR)	1:100,000<br>unreported (est. 50%)	100%	70%	3.13E-03	(179, 203); LOVD
FLNA	NM_001110556.1	Otopalatodigital syndrome, type 1 (XLD)	unreported (est. 1:1,000,000)<br>est. 100% (in males)	94%	16%	7.52E-08[E]	(204); LOVD
		Heterotopia, periventricular, 1 (XLD)	unreported (est. 1:500,000)<br>est. 100% (in males)	95%	10%[§]	9.50E-08[E]	(205)
		Frontometaphyseal dysplasia 1 (XLR)	unreported (est. 1:1,000,000)<br>unreported (est. 50%)	71%	10%[§]	1.19E-04[E]	(204)
FLNC	NM_001458.4	Cardiomyopathy, familial restrictive 5 (AD)	unreported (est. 1:333,000)<br>unreported (est. 50%)	unreported (100%[†])	6%	1.80E-07	(206); OMIM #617047, *102565; LOVD
FOXE3	NM_012186.2	Aortic aneurysm, familial thoracic 11 (AD)	1:1,000<br>50%	1.4%	100%	1.40E-05	(176, 207);<br>OMIM # 617349, *601094

GATA5	NM_080473.4	Congenital heart defects, multiple types, 5 (AR, AD)	1:100 (CHD in general)<br>incomplete (est. 50%)	2%	10%§	2.00E-05	(208-212); OMIM #617912, *611496
IPO8	NM_006390.4	VISS syndrome (AR)	unreported (est. 1:100,000)<br>100%	unreported (100%†)	10%§	3.16E-04	(213-215); OMIM #619472, *605600; ClinVar
KDR	NM_002253.2	Hemangioma, capillary infantil (AD, AR)	est. 1:10<br>unreported (est. 50%)	est. 1%	10%§	1.00E-04	(216); OMIM #602089; *191306
LOX	NM_002317.6	Aortic aneurysm, familial thoracic 10 (AD)	1:1,000<br>50%	1.5%	100%	1.50E-05	(40, 176)
LRP1	NM_002332.2	Aortic aneurysm, familial thoracic N.S. (risk factor)	1:1,000<br>50%	unreported (100%†)	10%§	1.00E-04	(217)
		Keratosis pilaris atrophicans (AR)	1:8<br>est. 50%	unreported (100%†)	10%§	1.00E-04	(218);<br>OMIM #604093
LTBP2	NM_000428.2	Weill-Marchesani syndrome 3 (AR)	1:100,000<br>100%	4%	20%	1.26E-04	(177); LOVD
LTBP3	NM_001130144.3	Dental anomalies and short stature (AR)	unreported (est. 1:1,000,000)<br>unreported (est. 50%)	100%	12,5%	1.70E-04	(219); OMIM, #601216, *602090, ClinVar
LTBP4	NM_003573.2	Cutis laxa, autosomal recessive, type IC (AR)	rare (est. 1:1,000,000)<br>unreported (est. 50%)	100%	15%	1.98E-04	(220); LOVD
MAT2A	NM_005911.5	Aortic aneurysm, familial thoracic N.S. (AD)	1:1,000<br>50%	1%	100%	1.00E-05	(40, 176)
MFAP5	NM_003480.3	Aortic aneurysm, familial thoracic 9 (AD)	1:1,000<br>50%	0.25%	100%	2.50E-06	(40, 176)
MYH11	NM_001040113.1	Aortic aneurysm, familial thoracic 4 (AD)	1:1,000<br>50%	1%	100%	1.00E-05	(40, 176)
MYLK	NM_053025.3	Aortic aneurysm, familial thoracic 7 (AD)	1:1,000<br>50%	1%	100%	1.00E-05	(40, 176)
NOTCH1	NM_017617.4	Aortic valve disease 1 (AD)	1:50-500 (1:275)<br>unreported (est. 50%)	5%	10%§	1.82E-05	(221, 222); OMIM #109730, *190198
		Adams-Oliver syndrome 5 (AD)	1:230,000<br>Incomplete (est. 50%)	23%	10%§	1.00E-07	(223)
NPR3	NM_001204375.2	Boudin-Mortier syndrome (AR)	unreported (est. 1:1,000,000)<br>unreported (est. 50%)	unreported (100%†)	10%§	1.41E-04	(224), OMIM #619543, *108962
PLOD1	NM_000302.3	Ehlers-Danlos syndrome, kyphoscoliotic type, 1 (AR)	1:100,000<br>100%	unreported (100%†)	30%	9.49E-04	(179, 225); LOVD
PLOD3	NM_001084.4	Lysyl hydroxylase 3 deficiency (AR, risc factor)	unreported (est. 1:1,000,000)<br>unreported (est. 50%)	unreported (100%†)	10%§	1.41E-04	(226); OMIM #612394, *603066
PMEPA1	NM_020182.5	Syndromic aortopathy, Loeys–Dietz syndrome (AD)	unreported (est. 1:100,000)<br>unreported (est. 50%)	20%-25%	50%	1.25E-06	(227)
PRDM5	NM_018699.3	Brittle cornea syndrome 2 (AR)	1:1,000,000<br>unreported (est. 50%)	unreported (100%†)	15%	2.12E-04	(179, 228); OMIM #614170, *614161; ORPHA:90354
PRKG1	NM_006258.3	Aortic aneurysm, familial thoracic 8 (AD)	1:1,000<br>50%	1%	100%	1.00E-05	(40, 176)

SECISBP2	NM_024077.5	Multisystem disorder with aortic aneurysmal disease (AR, risc factor)	unreported (est. 1:1,000,000) unreported (est. 50%)	unreported (100%†)	10%§	1.41E-04	(229)
SKI	NM_003036.3	Sphrintzen-Goldberg syndrome (AD)	rare (est. 1:1,000,000) unreported (est. 50%)	100%	10%	1.00E-07	(230, 231); ClinVar; OMIM #182212, *164780; LOVD
SLC2A10	NM_030777.3	Arterial tortuosity syndrome (AR)	rare (est. 1:1,000,000) unreported (est. 50%)	unreported (100%†)	20%	2.00E-07	(232); ClinVar; OMIM *606145, #208050; LOVD
SLC39A13	NM_152264.4	Ehlers-Danlos syndrome, spondylodysplastic type, 3 (AR)	unreported (est. 1:1,000,000) unreported (est. 50%)	unreported (100%†)	20%	2.83E-04	(179, 233); OMIM #612350, *608735
SMAD2	NM_005901.5	Aortic and arterial aneurysmal disease and connective tissue features (AD, risc factor)	unreported (est. 1:1,000,000) unreported (est. 50%)	unreported; appr. 20 cases decribed (100%†)	10%§	1.00E-07	(234-238)
		Loeys-Dietz syndrome (AD)	unreported (est. 1:100,000) 95%	1-5%	20%	5.26E-08	(238, 239); UMD
SMAD3	NM_005902.3	Aortic aneurysm, familial thoracic N.S. (AD)	1:1,000 50%	2%	100%	2.00E-05	(40, 176)
		Loeys-Dietz syndrome 3 (AD)	unreported (est. 1:100,000) 95%	5-10%	20%	1.05E-07	(238, 239); UMD
SMAD4	NM_005359.5	Juvenile polyposis/hereditary hemorrhagic telangiectasia syndrome (AD)	1:10,000 age-dependent (50%)	1%-2%	25%	5.00E-07	(240), LOVD
		Myhre syndrome (AD)	rare (1:1,000,000) 100%	100%	25%	2.50E-07	(241); LOVD
		Polyposis, juvenile intestinal (AD)	1:50,000 97%	27%	25%	6.96E-07	(242); LOVD
SOX18	NM_018419.2	Hypotrichosis-lymphedema-telangiectasia syndrome (AR)	unreported (est. 1:1,000,000) unreported (est. 50%)	unreported (100%†)	10%§	1.41E-04	(243); ORPHA:69735; OMIM #607823
		Hypotrichosis-lymphedema-telangiectasia-renal defect syndrome (AD)	unreported (est. 1:1,000,000) unreported (est. 50%)	unreported (100%†)	10%§	1.00E-07	(243); ORPHA:69735; OMIM #137940
TES	NM_015641.4	Aortic aneurysm, familial thoracic N.S. (AD)	1:1,000 50%	<1%	100%	1.00E-05	(244)
TGFB2	NM_001135599.2	Aortic aneurysm, familial thoracic (AD)	1:1,000 50%	1%	100%	1.00E-05	(40, 176)
		Loeys-Dietz syndrome 4 (AD)	unreported (est. 1:100,000) 95%	5%-10%	20%	1.05E-07	(238, 239); UMD
TGFB3	NM_003239.3	Aortic aneurysm, familial thoracic (AD)	1:1,000 50%	rare (est. 1%)	100%	1.00E-05	(40, 176)
		Loeys-Dietz syndrome 5 (AD)	unreported (est. 1:100,000) 95%	1%-5%	20%	5.26E-08	(238, 239); UMD
TGFBR1	NM_004612.3	Loeys-Dietz syndrome 1 (AD)	unreported (est. 1:100,000) 95%	20%-25%	20%	2.63E-07	(238, 239); UMD
		Aortic aneurysm, familial thoracic N.S. (AD)	1:1,000 50%	3%	100%	3.00E-05	(40, 176)

TGFBR2	NM_001024847.2	Loeys-Dietz syndrome 2 (AD)	unreported (est. 1:100,000)<br>95%	55%-60%	10-20%	6.32E-07	(238, 239, 245); LOVD; UMD
		Aortic aneurysm, familial thoracic N.S. (AD)	1:1,000<br>50%	5%	10%	5.00E-06	(40, 176); LOVD
TNXB	NM_019105.6	Ehlers-Danlos syndrome, classic-like, 1 (AR, AD; N.S.)	unreported (est. 1:1,000,000)<br>unreported (est. 50%)	unreported (100%†)	17%	2.40E-04	(179); OMIM #606408, *600985; ORPHA:230839; LOVD
THSD4	NM_024817.3	Aortic aneurysm, familial thoracic 12 (AD)	1:1,000<br>50%	rare (est. 1%)	100%	1.00E-05	(246); OMIM #619825, *614476
ULK4	NM_017886.3	Aortic aneurysm, familial thoracic N.S. (risc factor)	1:1,000<br>50%	rare (est. 1%)	100%	1.00E-05	(217)
ZNF469	NM_001127464.2	Brittle cornea syndrome 1 (AR)	1:1,000,000<br>unreported (est. 50%)	unreported (100%†)	15%	2.12E-04	(179, 228); OMIM #229200, *612078; ORPHA:90354
GENE 67 CDKL1	NM_004196.7<br>NM_001282236.2	Vasculopathy, familial (AD)	unreported (est 1:1,000)<br>50%	unreported (100%†)	10%§	1.00E-04	candidate disease gene<br>This study
GENE 68	N.G.	N.G.	N.G.<br>N.G.	N.G.	N.G.	N.G.	candidate disease gene
GENE 69	N.G.	N.G.	N.G.<br>N.G.	N.G.	N.G.	N.G.	candidate disease gene
GENE 70	N.G.	N.G.	N.G.<br>N.G.	N.G.	N.G.	N.G.	candidate disease gene
GENE 71	N.G.	N.G.	N.G.<br>N.G.	N.G.	N.G.	N.G.	candidate disease gene
GENE 72	N.G.	N.G.	N.G.<br>N.G.	N.G.	N.G.	N.G.	candidate disease gene

Supplemental Table 1 shows prevalence and penetrance of vascular/connective tissue disorders associated with 66 genes (from *ABL1* to *ZNF64*) and maximum calculated population frequency (MPF) of causative variants. MPF calculator is available at cardiodb.org/allelefrequencyapp/; accessed February 2022 or December 2024. For genes associated with more than one disorder (e.g. *ACTA2*, *COL2A1*) two or three representative disorders are given, and the highest MPF was used for variant prioritization (which is usually corresponding to the most prevalent condition). Gene 67 is *CDKL1*. Genes 68-72 are candidate disease genes identified in families with TAAD/HCTD and, thereby, subject of ongoing research. Genes analysed by targeted next-generation sequencing are indicated in bold font. **[A]**For disorders with unreported prevalence, information about similar disorders was adopted. **[B]**For disorders with unreported, incomplete or age-dependent penetrance, a value of 50% was estimated. **[C]**Maximum genetic contribution: proportion of families with pathogenic variant in this gene (112). If genetic heterogeneity of a disorder is not well characterized, we assume maximal genetic contribution (i.e., 100%; indicated by †), so that the disease is modeled as attributable to one gene. [D]Maximum allelic contribution: for disorders with well characterized allelic contribution we used various databases to estimate the maximum allelic contribution at the upper bound of a 95% confidence interval (CI) (112). Where no mutation database exists, we used what is known about similar disorders such as Marfan syndrome, vascular Ehlers-Danlos syndrome, etc. to estimate the maximum allelic contribution: no single variant causes more than 10% of cases (which is a very conservative estimation; indicated by §). If allelic heterogeneity of a disorder is not well characterized, we assume maximal allelic contribution (i.e., 100%), so that the contribution of each gene is modeled as attributable to one allele, and the maximum allelic contribution is substituted

by the maximum genetic contribution (i.e., the maximum proportion of the disease attributable to a single gene) (112). For example, familial thoracic aortic aneurysm (also known as non-syndromic TAAD) is caused by variants in the *MYLK* gene in approximately 1% of cases. Taking 0.01 as our maximum genetic contribution, a minimal allelic heterogeneity (i.e., maximal allelic contribution, 100%) and a population prevalence of 1:1,000, we derive a maximum tolerated population frequency of 0.0002). [E]For X-linked disorders we calculated the MPF by assuming monoallelic inheritance. ORPHA, Orphanet, An online database of rare diseases and orphan drugs. Copyright, INSERM 1997. Available at www.orpha.net. Accessed February 2022. OMIM, Online Mendelian Inheritance in Man, An Online Catalog of Human Genes and Genetic Disorders, Available at www.omim.org/. Accessed February 2022. LOVD, Leiden Open-source Variation Database (247), Available at www.lovd.nl/. Accessed February 2022. ClinVar, database on the relationships between human variations and phenotypes (248). Available at www.ncbi.nlm.nih.gov/clinvar/. Accessed February 2022. UMD, The UMD mutations database. Available at www.umd.be. Accessed February 2022. AD, autosomal dominant; AR, autosomal recessive; XLD, X-linked dominant; XLR, X-linked recessive; XL, X-linked; N.S., not solved; est., estimated; N.A., not applicable; N.G., not given.

Supplemental Table 2. Peptide substrates with decreased or increased phosphorylation in CDKL1^{Cys143Arg}-, CDKL1^{Ser206Leu}-, or CDKL1^{Thr135Met}-treated samples versus CDKL1^{WT}

Pept. nos.	Protein name_ amino acids	Peptide sequence	Ser sites	Thr sites	Uniprot Acc.	Description
**1**	ART_025_CXGLRRWSLGGLRRWSL	GLRRWSLGGLRRWSL	NA	NA	NA	Artificial control peptide
**2**	RBL2_655_667	GLGRSITSPTTLY	[659, 662]	[661, 664, 665]	Q08999	Retinoblastoma-like protein 2 (130 kDa retinoblastoma-associated protein) (p130) (PRB2) (RBR-2)
**3**	ACM1_421_433	CNKAFRDTFRLLL	[]	[428]	P11229	Muscarinic acetylcholine receptor M1
**4**	ACM1_444_456	KIPKRPGSVHRTP	[451]	[455]	P11229	Muscarinic acetylcholine receptor M1
**5**	ERBB2_679_691	QQKIRKYTMRRLL	[]	[686]	P04626	Receptor tyrosine-protein kinase erbB-2 precursor (p185erbB2) (C-erbB-2) (NEU proto-oncogene) (Tyrosine kinase-type cell surface receptor HER2) (MLN 19) (CD340 antigen)
**6**	FOXO3_25_37	QSRPRSCTWPLQR	[26, 30]	[32]	O43524	Forkhead box protein O3 (Forkhead in rhabdomyosarcoma-like 1) (AF6q21protein)
**7**	GPR6_349_361	QSKVPFRSRSPSE	[350, 356, 358, 360]	[]	P46095	Sphingosine 1-phosphate receptor GPR6 (G-protein coupled receptor 6)
**8**	GYS2_1_13	MLRGRSLSVTSLG	[6, 8, 11]	[10]	P54840	Glycogen [starch] synthase, liver
**9**	MARCS_152_164	KKKKKRFSFKKSF	[159, 163]	[]	P29966	Myristoylated alanine-rich C-kinase substrate (MARCKS) (Protein kinase C substrate, 80 kDa protein, light chain) (PKCSL) (80K-L protein)
**10**	MARCS_160_172	FKKSFKLSGFSFK	[163, 167, 170]	[]	P29966	Myristoylated alanine-rich C-kinase substrate (MARCKS) (Protein kinase C substrate, 80 kDa protein, light chain) (PKCSL) (80K-L protein)
**11**	MBP_222_234	HFFKNIVTPRTPP	[]	[229, 232]	P02686	Myelin basic protein (MBP) (Myelin A1 protein) (Myelin membrane encephalitogenic protein)
**12**	MP2K1_287_299	PPRPRTPGRPLSS	[298, 299]	[292]	Q02750	Dual specificity mitogen-activated protein kinase kinase 1 (MAP kinase kinase 1) (MAPKK 1) (ERK activator kinase 1) (MAPK/ERK kinase 1) (MEK1)
**13**	MPIP1_172_184	FTQRQNSAPARML	[178]	[173]	P30304	M-phase inducer phosphatase 1 (Dual specificity phosphatase Cdc25A)
**14**	NOS3_1171_1183	SRIRTQSFSLQER	[1171, 1177, 1179]	[1175]	P29474	Nitric oxide synthase, endothelial (Endothelial NOS) (eNOS) (NOS type III) (NOSIII) (Constitutive NOS) (cNOS)
**15**	PLEK_106_118	GQKFARKSTRRSI	[113, 117]	[114]	P08567	Pleckstrin (Platelet p47 protein)
**16**	RYR1_4317_4329	VRRLRRLTAREAA	[]	[4324]	P21817	Ryanodine receptor 1 (Skeletal muscle-type ryanodine receptor) (RyR1)(RYR-1) (Skeletal muscle calcium release channel)
**17**	ACM4_456_468	CNATFKKTFRHLL	[]	[459, 463]	P08173	Muscarinic acetylcholine receptor M4
**18**	ACM5_494_506	CYALCNRTFRKTF	[]	[501, 505]	P08912	Muscarinic acetylcholine receptor M5
**19**	ACM5_498_510	CNRTFRKTFKMLL	[]	[501, 505]	P08912	Muscarinic acetylcholine receptor M5

**20**	ADDB_696_708	GSPSKSPSKKKKK	[697, 699, 701, 703]	[]	P35612	Beta-adducin (Erythrocyte adducin subunit beta)
**21**	ADDB_706_718	KKKFRTPSFLKKS	[713, 718]	[711]	P35612	Beta-adducin (Erythrocyte adducin subunit beta)
**22**	ANDR_785_797	VRMRHLSQEFGWL	[791]	[]	P10275	Androgen receptor (Dihydrotestosterone receptor) (Nuclear receptor subfamily 3 group C member 4)
**23**	BAD_93_105	FRGRSRSAPPNLW	[97, 99]	[]	Q92934	Bcl2 antagonist of cell death (BAD) (Bcl-2-binding component 6) (Bcl-XL/Bcl-2-associated death promoter) (Bcl-2-like 8 protein)
**24**	CA2D1_494_506	LEDIKRLTPRFTL	[]	[501, 505]	P54289	Voltage-dependent calcium channel subunit alpha-2/delta-1 precursor(Voltage-gated calcium channel subunit alpha-2/delta-1) [Contains: Voltage-dependent calcium channel subunit alpha-2-1; Voltage-dependent calcium channel subunit delta-1]
**25**	CD27_212_224	HQRRKYRSNKGES	[219, 224]	[]	P26842	CD27 antigen precursor (CD27L receptor) (T-cell activation antigenCD27) (T14) (Tumor necrosis factor receptor superfamily member 7)
**26**	CENPA_1_14	MGPRRRSRKPEAPR	[7]	[]	P49450	Histone H3-like centromeric protein A (Centromere protein A) (CENP-A)(Centromere autoantigen A)
**27**	GSUB_61_73	KKPRRKDTPALHI	[]	[68]	O96001	G-substrate
**28**	H2B1B_ 27_40	GKKRKRSRKESYSI	[33, 37, 39]	[]	P33778	Histone H2B type 1-B (H2B.f) (H2B/f) (H2B.1)
**29**	H32_3_18	RTKQTARKSTGGKAPR	[11]	[4, 7, 12]	Q71DI3	Histone H3.2 (H3/m) (H3/o)
**30**	IF4E_203_215	TATKSGSTTKNRF	[207, 209]	[203, 205, 210, 211]	P06730	Eukaryotic translation initiation factor 4E (eIF-4E) (eIF4E) (mRNA cap-binding protein) (eIF-4F 25 kDa subunit)
**31**	KAPCG_192_206	VKGRTWTLCGTPEYL	[]	[196, 198, 202]	P22612	cAMP-dependent protein kinase catalytic subunit gamma (PKA C-gamma)
**32**	KPCB_19_31_A25S	RFARKGSLRQKNV	[25]	[]	P05771	Protein kinase C beta type (PKC-beta) (PKC-B)
**33**	KS6A1_374_386	QLFRGFSFVATGL	[380]	[384]	Q15418	Ribosomal protein S6 kinase alpha-1 (S6K-alpha 1) (90 kDa ribosomal protein S6 kinase 1) (p90-RSK 1) (pp90RSK1) (p90S6K) (Ribosomal S6 kinase 1) (RSK-1) (MAP kinase-activated protein kinase 1a) (MAPKAPK1A)
**34**	NR4A1_344_356	GRRGRLPSKPKQP	[351]	[]	P22736	Nuclear receptor subfamily 4 group A member 1 (Orphan nuclear receptorHMR) (Early response protein NAK1) (TR3 orphan receptor) (ST-59)
**35**	PPR1A_28_40	QIRRRRPTPATLV	[]	[35, 38]	Q13522	Protein phosphatase 1 regulatory subunit 1A (Protein phosphatase inhibitor 1) (IPP-1) (I-1).
**36**	RAP1B_172_184	PGKARKKSSCQLL	[179, 180]	[]	P61224	Ras-related protein Rap-1b precursor (GTP-binding protein smg p21B)
**37**	RB_242_254	AVIPINGSPRTPR	[249]	[252]	P06400	Retinoblastoma-associated protein (PP110) (P105-RB)
**38**	RB_350_362	SFETQRTPRKSNL	[350, 360]	[353, 356]	P06400	Retinoblastoma-associated protein (PP110) (P105-RB)
**39**	RB_803_815	NIYISPLKSPYKI	[807, 811]	[]	P06400	Retinoblastoma-associated protein (PP110) (P105-RB)
**40**	ADRB2_338_350	ELLCLRRSSLKAY	[345, 346]	[]	P07550	Beta-2 adrenergic receptor (Beta-2 adrenoceptor) (Beta-2adrenoreceptor).

**41**	CREB1_126_138	EILSRRPSYRKIL	[129, 133]	[]	P16220	cAMP response element-binding protein (CREB)
**42**	CSF1R_701_713	NIHLEKKYVRRDS	[713]	[]	P07333	Macrophage colony-stimulating factor 1 receptor precursor (CSF-1-R) (Fms proto-oncogene) (c-fms) (CD115 antigen)
**43**	KIF2C_105_118_S 106G	EGLRSRSTRMSTVS	[109, 111, 115, 118]	[112, 116]	Q99661	Kinesin-like protein KIF2C (Mitotic centromere-associated kinesin)(MCAK) (Kinesin-like protein 6)
**44**	PTN12_32_44	FMRLRRLSTKYRT	[39]	[40, 44]	Q05209	Tyrosine-protein phosphatase non-receptor type 12 (Protein-tyrosine phosphatase G1) (PTPG1) (PTP-PEST)
**45**	RS6_228_240	IAKRRRLSSLRAS	[235, 236, 240]	[]	P62753	40S ribosomal protein S6 (Phosphoprotein NP33)
**46**	CAC1C_1974_198 6	ASLGRRASFHLEC	[1975, 1981]	[]	Q13936	Voltage-dependent L-type calcium channel subunit alpha-1C (Voltage-gated calcium channel subunit alpha Cav1.2) (Calcium channel, L type,alpha-1 polypeptide, isoform 1, cardiac muscle)
**47**	CDN1A_139_151	GRKRRQTSMTDFY	[146]	[145, 148]	P38936	Cyclin-dependent kinase inhibitor 1 (p21) (CDK-interacting protein 1) (Melanoma differentiation-associated protein 6) (MDA-6)
**48**	CFTR_761_773	LQARRRQSVLNLM	[768]	[]	P13569	Cystic fibrosis transmembrane conductance regulator (CFTR) (cAMP-dependent chloride channel) (ATP-binding cassette transporter sub-family C member 7)
**49**	E1A_ADE05_212_ 224	AILRRPTSPVSRE	[219, 222]	[218]	P03255	Early E1A 32 kDa protein
**50**	ESR1_160_172	GGRERLASTNDKG	[167]	[168]	P03372	Estrogen receptor (ER) (Estradiol receptor) (ER-alpha) (Nuclear receptor subfamily 3 group A member 1)
**51**	F263_454_466	NPLMRRNSVTPLA	[461]	[463]	Q16875	6-phosphofructo-2-kinase/fructose-2,6-biphosphatase 3 (6PF-2-K/Fru-2,6-P2ASE brain/placenta-type isozyme) (iPFK-2) (Renal carcinoma antigen NY-REN-56) [Includes: 6-phosphofructo-2-kinase;Fructose-2,6-bisphosphatase]
**52**	GBRB2_427_439	SRLRRRASQLKIT	[427, 434]	[439]	P47870	Gamma-aminobutyric acid receptor subunit beta-2 precursor (GABA(A)receptor subunit beta-2)
**53**	GRIK2_708_720	FMSSRRQSVLVKS	[710, 711, 715, 720]	[]	Q13002	Glutamate receptor, ionotropic kainate 2 precursor (Glutamate receptor6) (GluR-6) (GluR6) (Excitatory amino acid receptor 4) (EAA4)
**54**	KAP3_107_119	NRFTRRASVCAEA	[114]	[110]	P31323	cAMP-dependent protein kinase type II-beta regulatory subunit.
**55**	KCNA6_504_516	ANRERRPSYLPTP	[511]	[515]	P17658	Potassium voltage-gated channel subfamily A member 6 (Voltage-gated potassium channel subunit Kv1.6) (HBK2)
**56**	LIPS_944_956	GFHPRRSSQGATQ	[950, 951]	[955]	Q05469	Hormone-sensitive lipase (HSL)
**57**	MYPC3_268_280	LSAFRRTSLAGGG	[269, 275]	[274]	Q14896	Myosin-binding protein C, cardiac-type (Cardiac MyBP-C) (C-protein, cardiac muscle isoform)
**58**	NCF1_296_308	RGAPPRRSSIRNA	[303, 304]	[]	P14598	Neutrophil cytosol factor 1 (NCF-1) (Neutrophil NADPH oxidase factor1) (47 kDa neutrophil oxidase factor) (p47-phox) (NCF-47K) (47 kDa autosomal chronic

						granulomatous disease protein) (Nox organizer 2)(Nox-organizing protein 2) (SH3 and PX domain-containing protein 1A)
**59**	NCF1_321_333	QDAYRRNSVRFLQ	[328]	[]	P14598	Neutrophil cytosol factor 1 (NCF-1) (Neutrophil NADPH oxidase factor1) (47 kDa neutrophil oxidase factor) (p47-phox) (NCF-47K) (47 kDa autosomal chronic granulomatous disease protein) (Nox organizer 2)(Nox-organizing protein 2) (SH3 and PX domain-containing protein 1A)
**60**	PTK6_436_448	ALRERLSSFTSYE	[442, 443, 446]	[445]	Q13882	Tyrosine-protein kinase 6 (Breast tumor kinase)(Tyrosine-protein kinase BRK)
**61**	SCN7A_898_910	KNGCRRGSSLGQI	[905, 906]	[]	Q01118	Sodium channel protein type 7 subunit alpha (Sodium channel protein type VII subunit alpha) (Putative voltage-gated sodium channel subunitalpha Nax) (Sodium channel protein cardiac and skeletal muscle subunit alpha)
**62**	STK6_283_295	SSRRTTLCGTLDY	[283, 284]	[287, 288, 292]	O14965	Serine/threonine-protein kinase 6 (Aurora kinase A)(Aurora-A) (Serine/threonine kinase 15) (Aurora/IPL1-related kinase 1)(Aurora-related kinase 1) (hARK1) (Breast tumor-amplified kinase)
**63**	TOP2A_1463_1475	RRKRKPSTSDDSD	[1469, 1471, 1474]	[1470]	P11388	DNA topoisomerase 2-alpha (DNA topoisomerase II, alpha isozyme)
**64**	TY3H_65_77	FIGRRQSLIEDAR	[71]	[]	P07101	Tyrosine 3-monooxygenase (Tyrosine 3-hydroxylase) (TH).
**65**	VTNC_390_402	NQNSRRPSRATWL	[393, 397]	[400]	P04004	Vitronectin precursor (Serum-spreading factor) (S-protein) (V75)[Contains: Vitronectin V65 subunit; Vitronectin V10 subunit; Somatomedin-B]
**66**	KAP2_92_104	SRFNRRVSVCAET	[92, 99]	[104]	P13861	cAMP-dependent protein kinase type II-alpha regulatory subunit
**67**	NMDZ1_890_902	SFKRRRSSKDTST	[890, 896, 897, 901]	[900, 902]	Q05586	Glutamate [NMDA] receptor subunit zeta-1 precursor (N-methyl-D-aspartate receptor subunit NR1)

Peptide numbers (Pept. nos.) of peptides correspond to peptide numbers in Figure 5D. Protein names, amino acid positions, peptide sequences, positions of potential serine and threonine phosphorylation sites (Ser sites, Thr sites), Uniprot accessions and protein descriptions are given. PamChip 4 kinase peptide substrates were used for STK-PamChip array (article code 87102; www.pamgene.com).

Supplemental Table 3. Quantitative proteomics of 1,304 proteins (see spreadsheet in online supplement)

Supplemental Table 4. Antibodies used for immunoblotting

Antibody class/type	Antibody name	Antibody source; product number; applied antibody dilution
Primary rabbit polyclonal antibodies	CDKL1	Thermo Fisher Scientific; PA5-101142; 1:1,000
	CDKL1	Merck, Sigma-Aldrich; HPA05960; 1:1,000
	CFAP20 (GTL3)	Thermo Fisher Scientific; PA5-96577; 1:1,000
	MEK1/2	Cell Signaling Tech.; no. 9122; 1:1,000
	phospho-MEK1/2 (Ser217/221)	Cell Signaling Tech.; no. 9121; 1:1,000
	p44/42 MAP kinase (ERK1/2)	Cell Signaling Tech.; no. 9102, 1:1,000
	phospho-p44/42 MAP kinase (ERK1/2) (Thr202/Tyr204)	Cell Signaling Tech.; no. 9101, 1:1,000
	Akt1/2/3	Cell Signaling Tech.; no. 9272; 1:1,000
	phospho-Akt1/2/3 (Ser473)	Cell Signaling Tech.; no. 9271; 1:1,000
	SAPK/JNK	Cell Signaling Tech.; no. 9252; 1:500
	phospho-SAPK/JNK (Thr183/Tyr185)	Cell Signaling Tech.; no. 9251; 1:1,000
	phospho-SMAD2 (Ser245/250/255)	Cell Signaling Tech.; no. 3104; 1:1,000
	phospho-SMAD3 (Ser204)	abcam, Cambridge, UK; ab63402; 1:500
	phospho-SMAD3 (Ser208)	Santa Cruz Biotechnology, Inc.; sc-130218; 1:200
	phospho-SMAD3 (Ser213)	abcam; ab63403; 1:500
	phospho-Cyclin E (Thr62)	Invitrogen, Thermo Fisher Scientific; PA5-17560; 1:1,000
	phospho-CDK2 (Thr160)	Cell Signaling Tech.; no. 2561; 1:1,000
	phospho-p21 (Thr145)	Thermo Fisher Scientific; PA5-12646; 1:1,000
	Dishevelled 2 (Dvl2)	Cell Signaling Tech.; no. 3216; 1:1,000
	phospho-β-Catenin (Thr41/Ser45)	Cell Signaling Tech.; no. 9565; 1:500
	IFT52	Thermo Fisher Scientific; # 17534-1-AP; 1:1,000
	IFT172	Thermo Fisher Scientific; # 28441-1-AP; 1:1,500
Primary goat polyclonal antibody	phospho-SMAD3 (Ser423/425)	Santa Cruz Biotechnology, Inc., sc-11769; 1:200
Primary rabbit monoclonal antibodies	p38	Cell Signaling Tech.; no. 8690; 1:1,000
	SMAD2/3 (D7G7)	Cell Signaling Tech.; no. 8685; 1:1,000
	CDK2	Cell Signaling Tech.; no. 2546; 1:1,000
	p21 Waf1/cip1	Cell Signaling Tech.; no. 2947, 1:1,000
	phospho-p38 (Thr180/Tyr182) (D3F9)	Cell Signaling Tech.; no. 4511; 1:1,000
	Cyclin D1 (92G2)	Cell Signaling Tech.; no. 2978; 1:500
	Shh (C9C5)	Cell Signaling Tech.; no. 2207; 1:1,000
	SUFU (C54G2)	Cell Signaling Tech.; no. 2520; 1:1,000
	GLI1	Cell Signaling Tech.; no. 3538; 1:1,000

	GLI3	Cell Signaling Tech.; no. 71107; 1:1,000
	phospho-Dishevelled 2 (Dvl2) (Ser143)	abcam; ab124933; 1:1,000
	Cleaved Notch1 (Val1744) (D3B8)	Cell Signaling Tech.; no. 4147; 1:1,000
	HES1 (d6P2U)	Cell Signaling Tech.; no. 11988; 1:1,000
Primary mouse monoclonal antibodies	GFP Epitope Tag	BioLegend; no. 902601; 1:5,000
	GAPDH	GeneTex Inc.; GTX28245; 1:10,000
	β-Catenin (L87A12)	Cell Signaling Tech.; no. 2698; 1:1,000
	Cyclin E (HE12)	Cell Signaling Tech.; no. 4129; 1:1,000
	Cyclin D3 (DCS22)	Cell Signaling Tech.; no. 2936S; 1:1,000
	c-Myc	Sigma-Aldrich; M5546; 1:1,000
	Anti-TUBA1A	Merck; T8203-25UL; 1:1,000
Secondary antibodies	horseradish peroxidase (HRP)-coupled anti-rabbit	GE Healthcare; NA934V; 1: 7,500
	horseradish peroxidase (HRP)-coupled anti-mouse	GE Healthcare; NA931V; 1: 7,500
	goat anti-mouse IgG StarBright Blue 700 Fluorophore	BioRad; 12004159, 1:10,000
	goat anti-rabbit IgG StarBright Blue 520 Fluorophore	BioRad; 12005869, 1:10,000
	donkey anti-goat DyLight 800 antibodies	Invitrogen; SA5-10092, 1:10,000
Loading control antibody	hFAB™ Rhodamine Anti-GAPDH	BioRad; 12004167; 1:10,000
