# Supplementary material for: CDKL1 variants affecting ciliary formation predispose to thoracic aortic aneurysm and dissection
Source: J Clin Invest. 2025 Oct 7;135(23):e186287. doi: 10.1172/JCI186287 (PMC12646653; doi:10.1172/JCI186287)

Full unedited blots for Supplemental Figure 7B

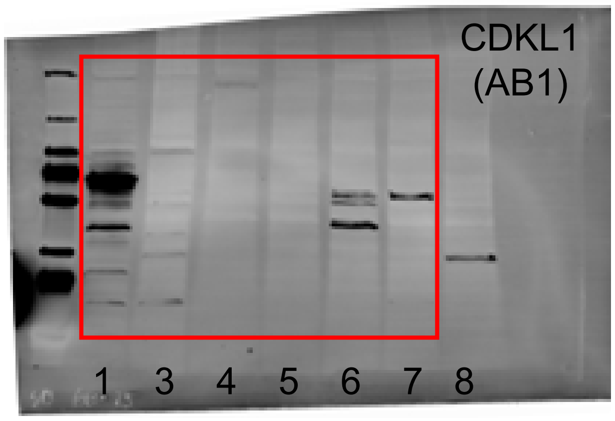

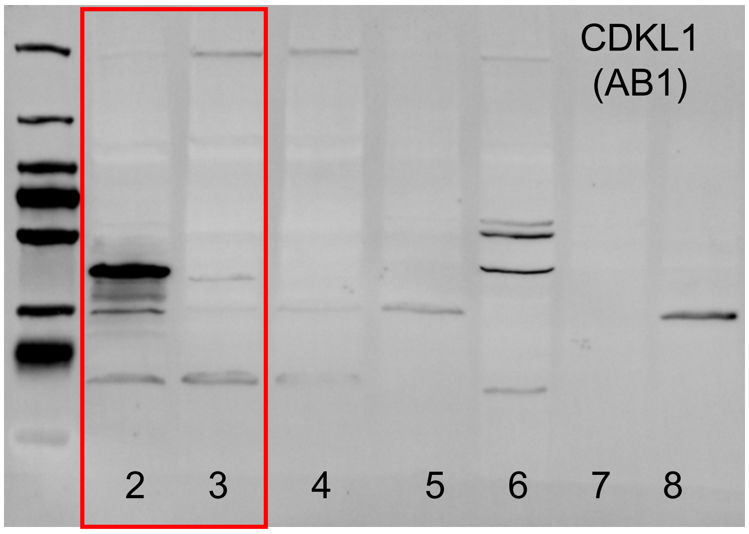

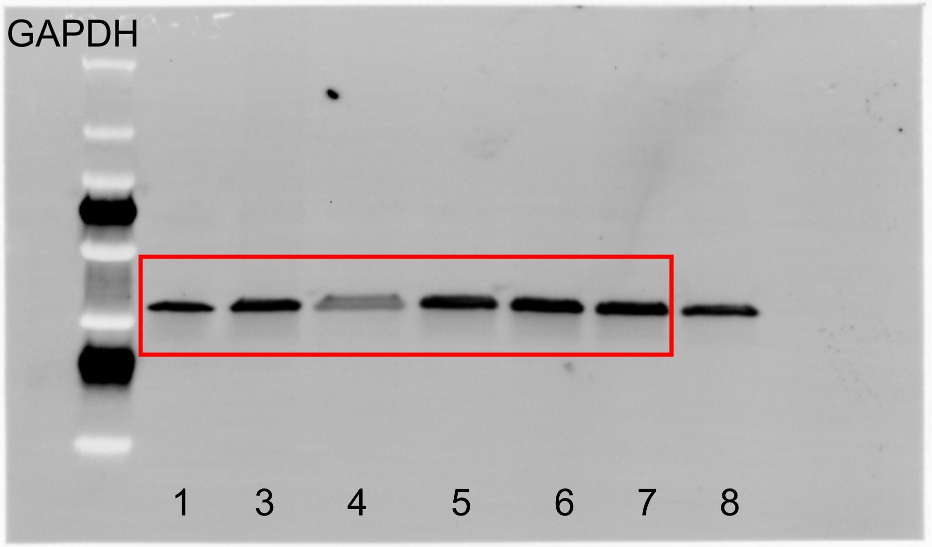

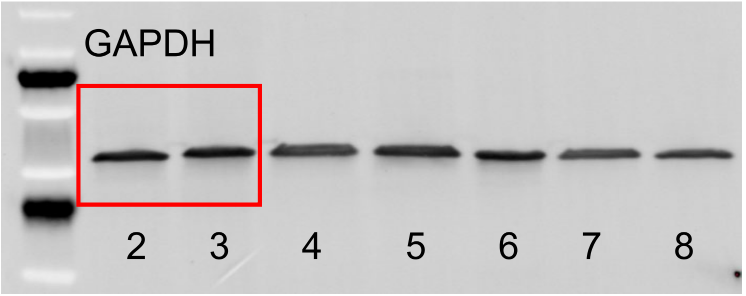

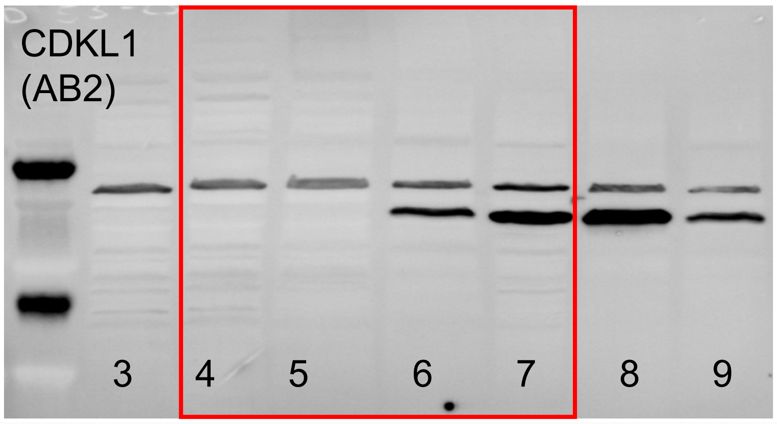

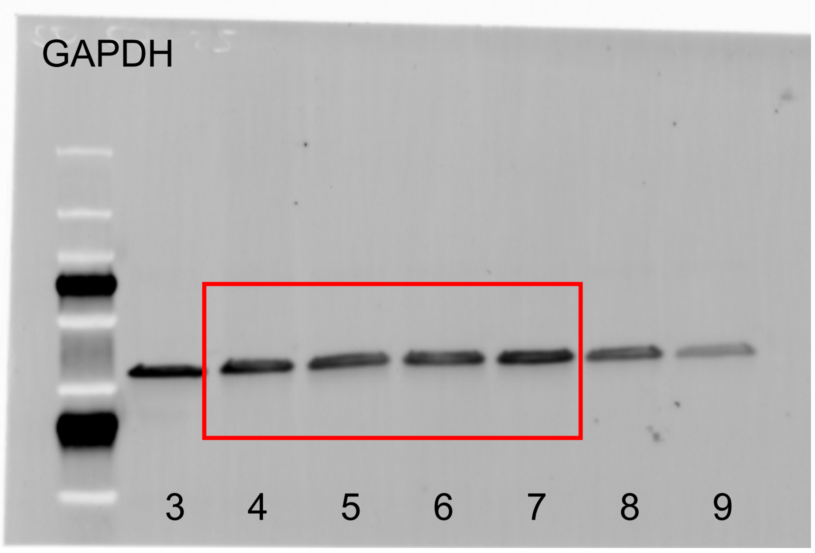

Lane 1: EGFP-CDKL1^{WT} in HEK293T

Lane 2: HA-CDKL1^{WT} in HEK293T

Lane 3: HEK293T

Lane 4: A10

Lane 5: HeLa

Lane 6: Colo205

Lane 7: RT4

Lane 8: EA.hy926

Lane 9: A431

Full unedited blot for Supplemental Figure 8C

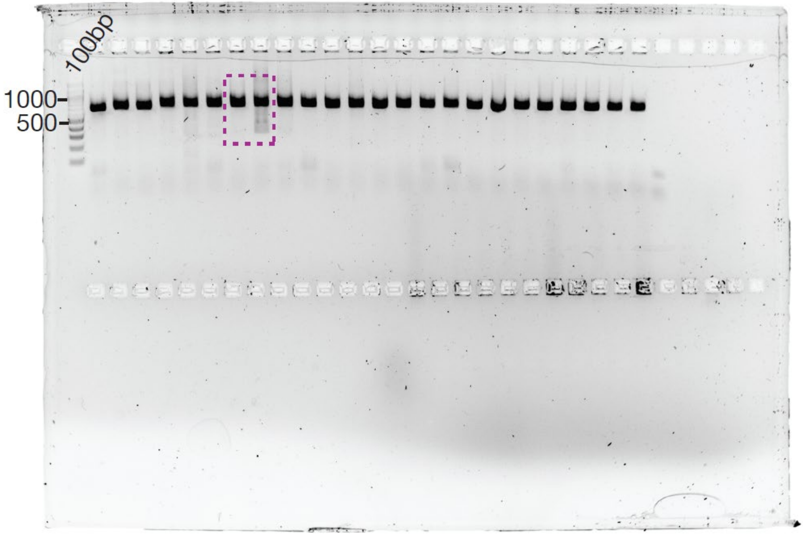

Full unedited blots for Supplemental Figure 9C

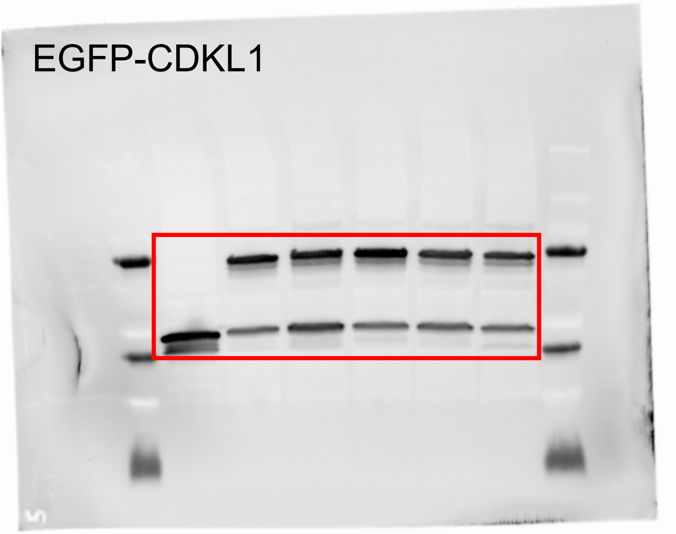

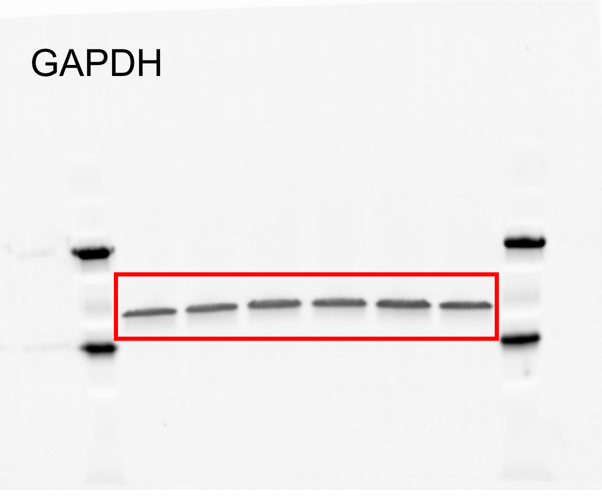

Full unedited blots for Supplemental Figure 12

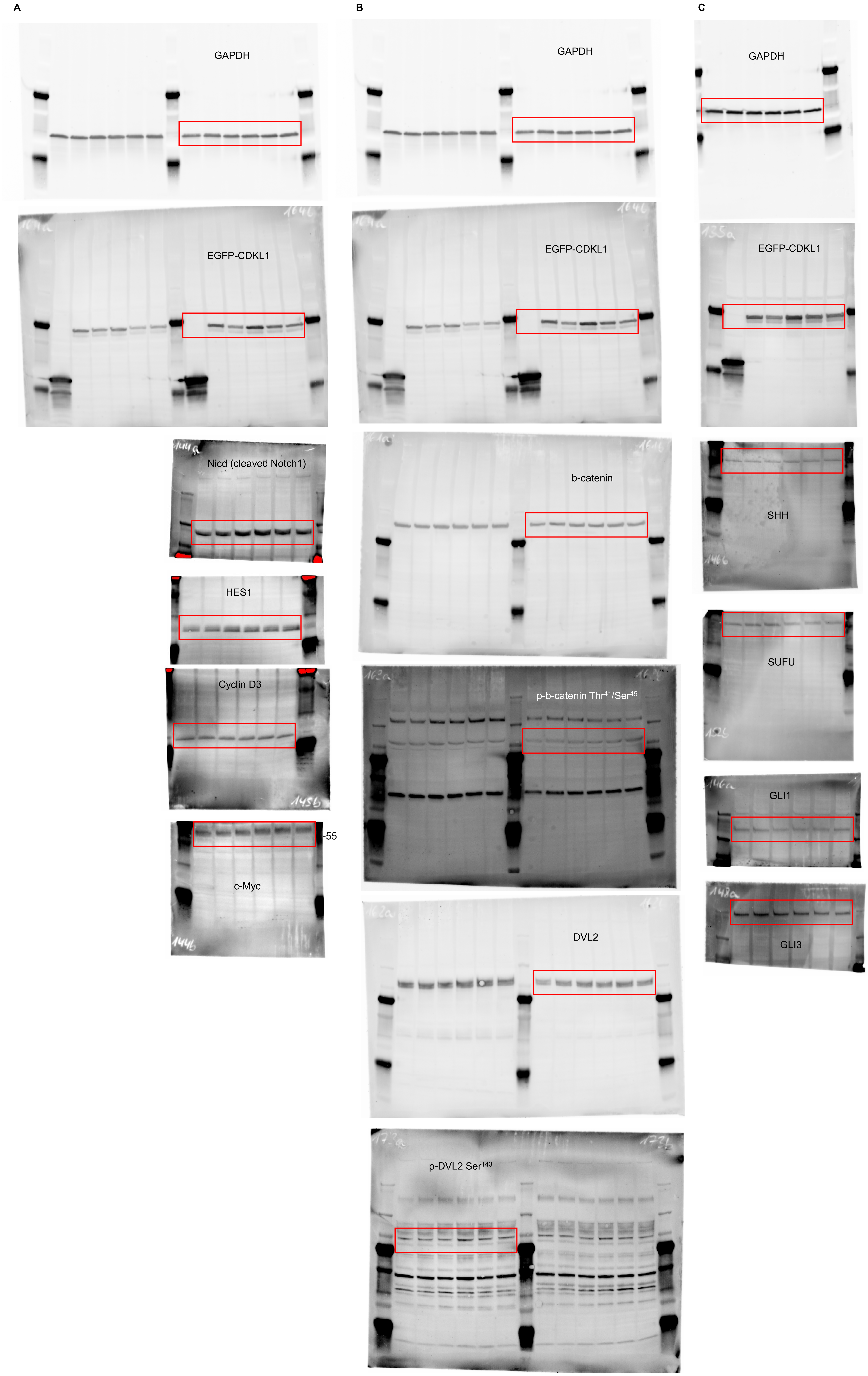

Full unedited blots for Supplemental Figure 13

A

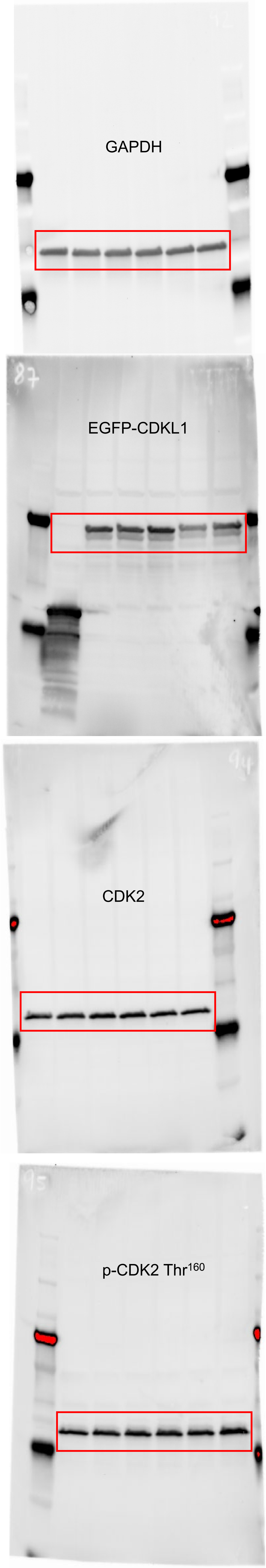

B

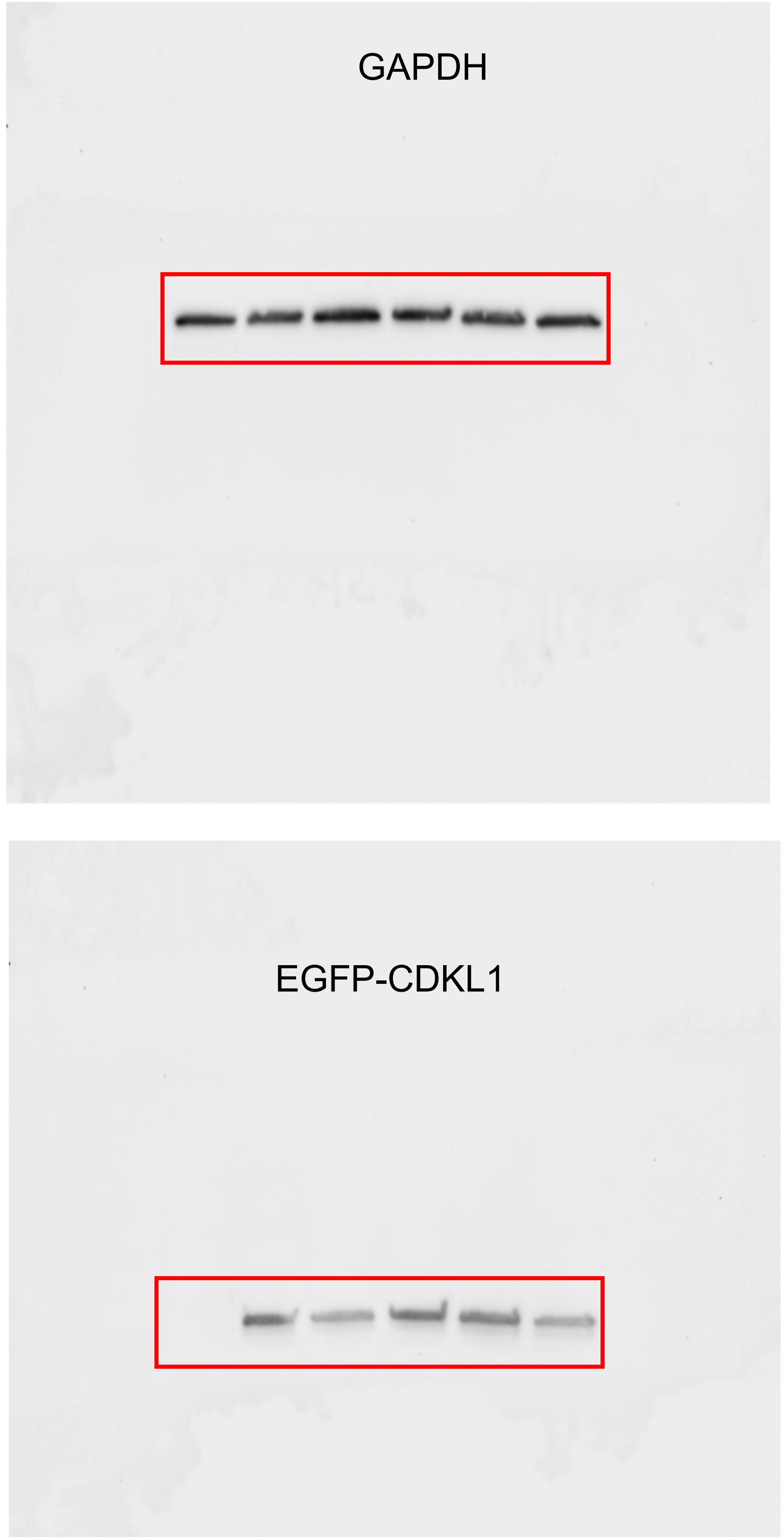

Note: EGFP signal in the first lane is missing because the western blot membrane was cut apart over the EGFP band in order to be able to detect other target proteins in parallel on the lower part of the membrane.

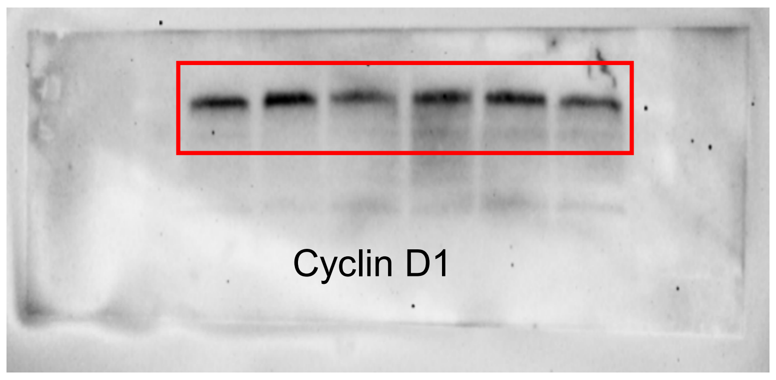

C

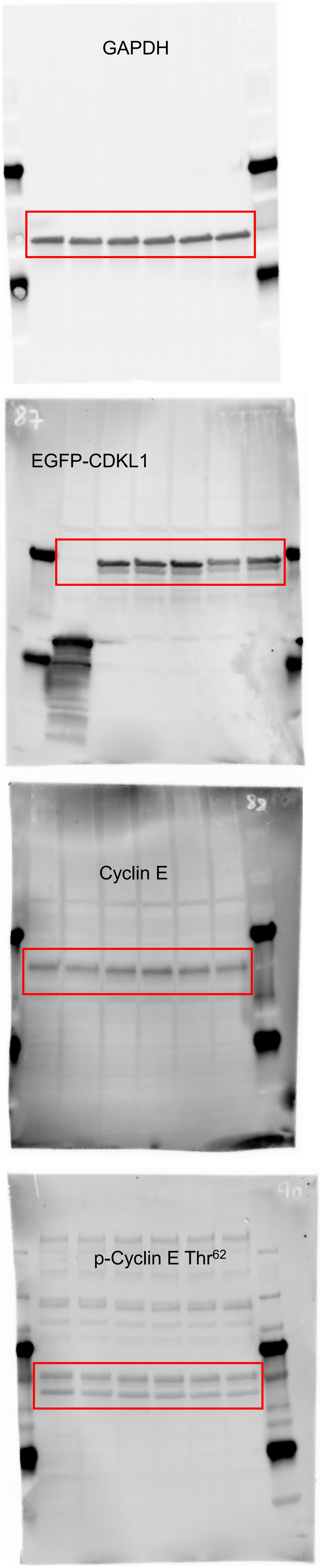

D

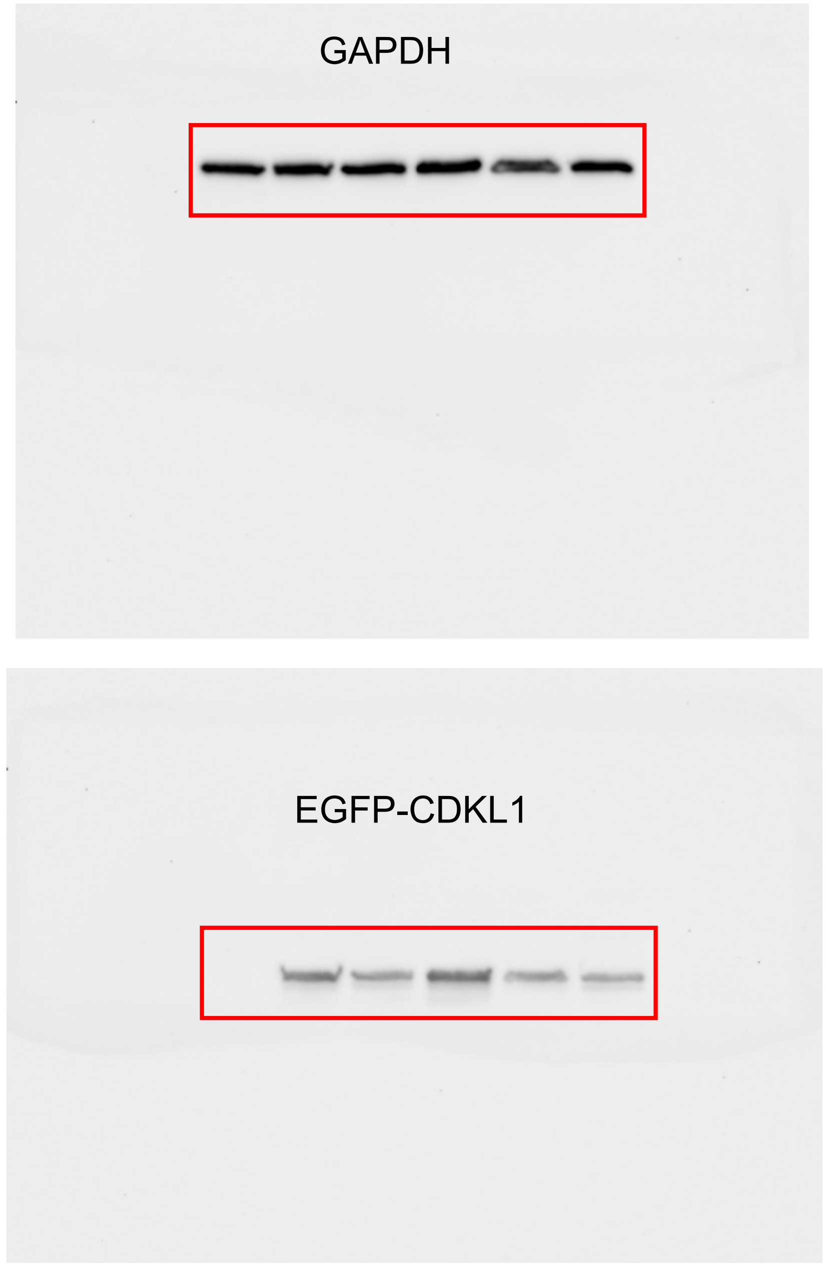

Note: EGFP signal in the first lane is missing because the western blot membrane was cut apart over the EGFP band in order to be able to detect other target proteins in parallel on the lower part of the membrane.

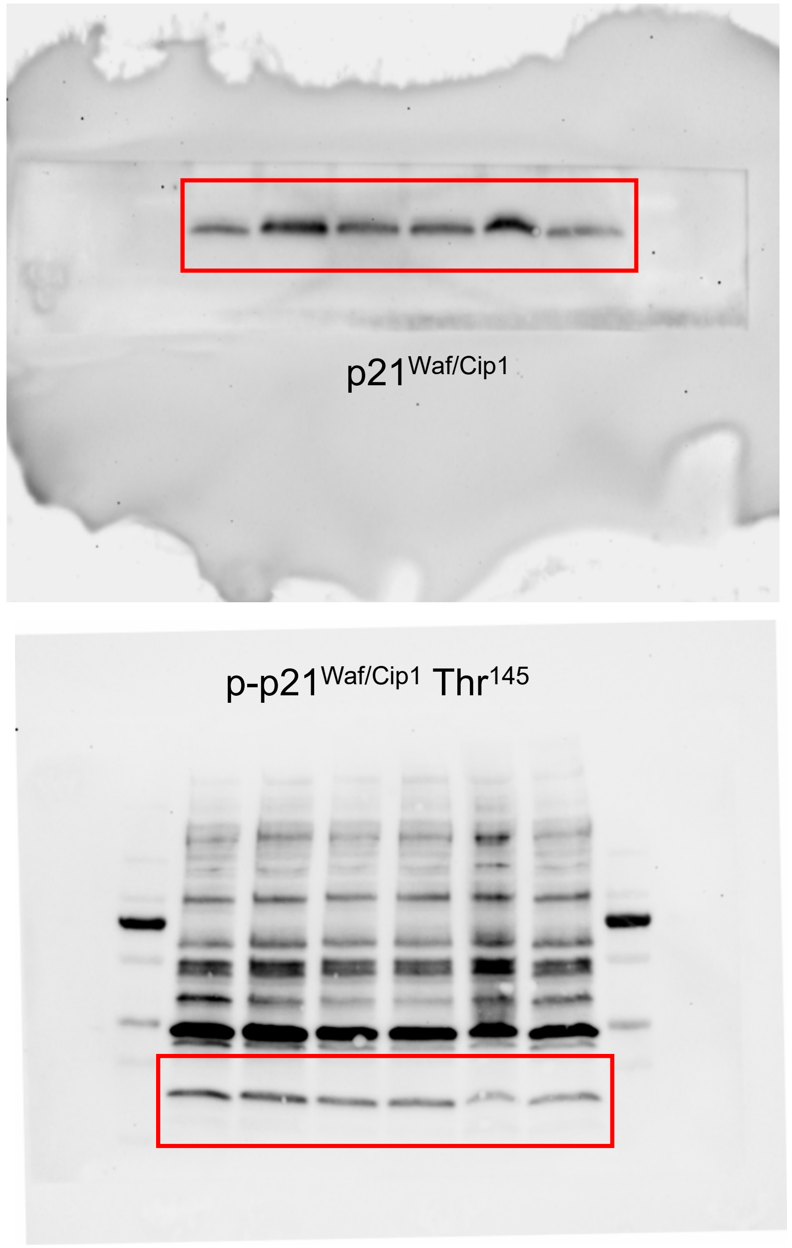

Full unedited blots for Supplemental Figure 14

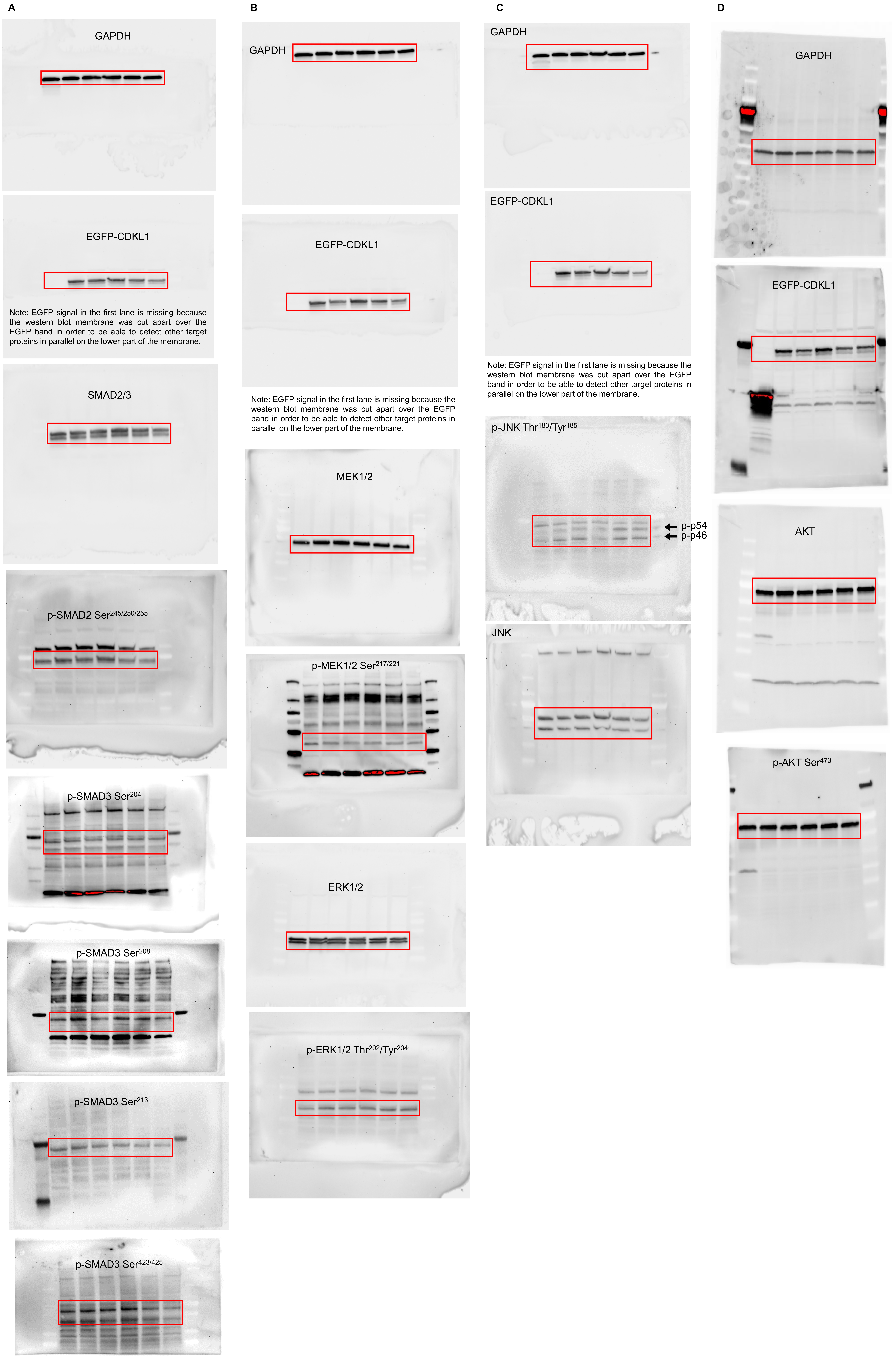

Full unedited blots for Figure 5

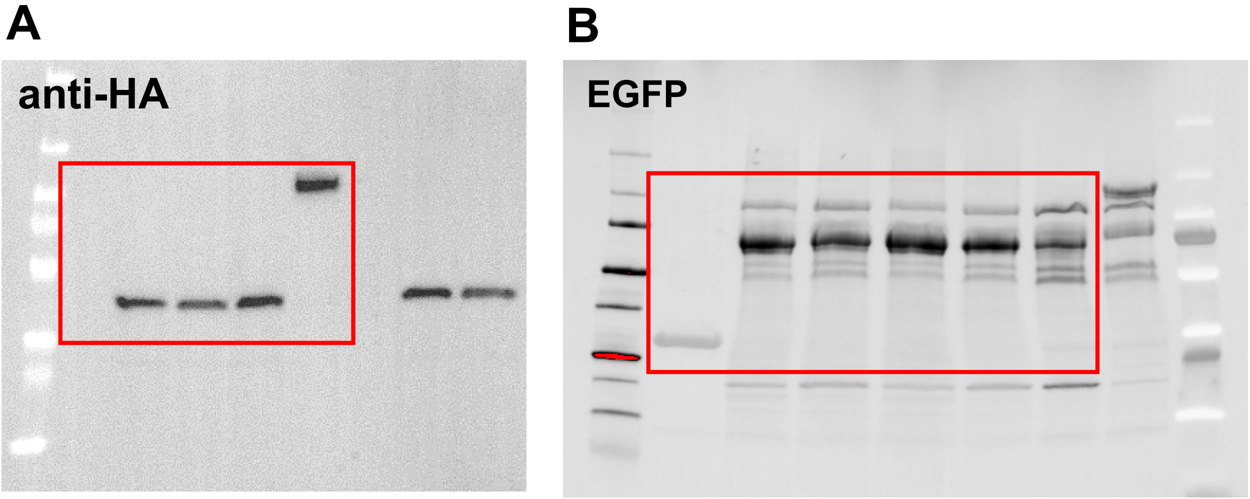

Full unedited blots for Figure 6C

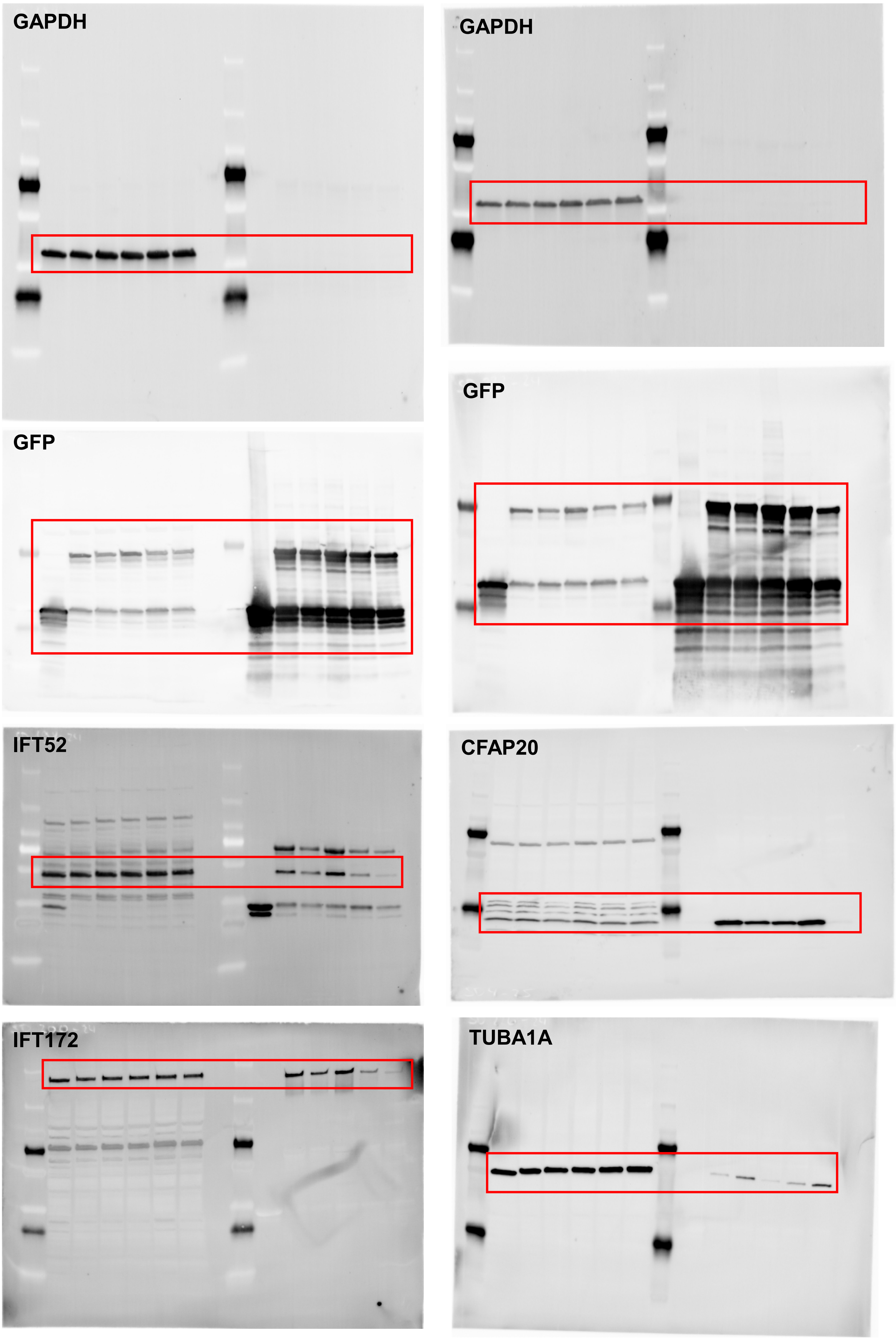

Full unedited blots for Figure 8

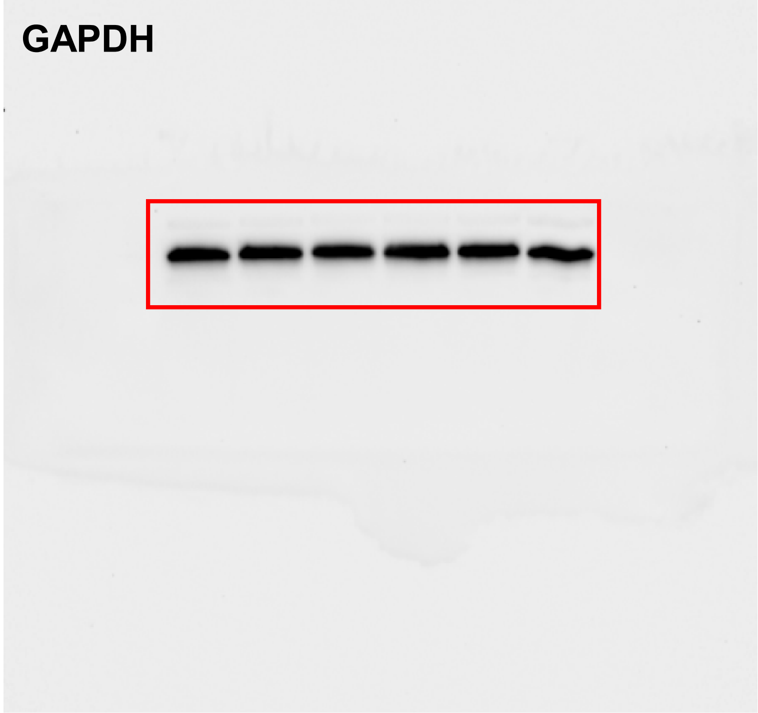

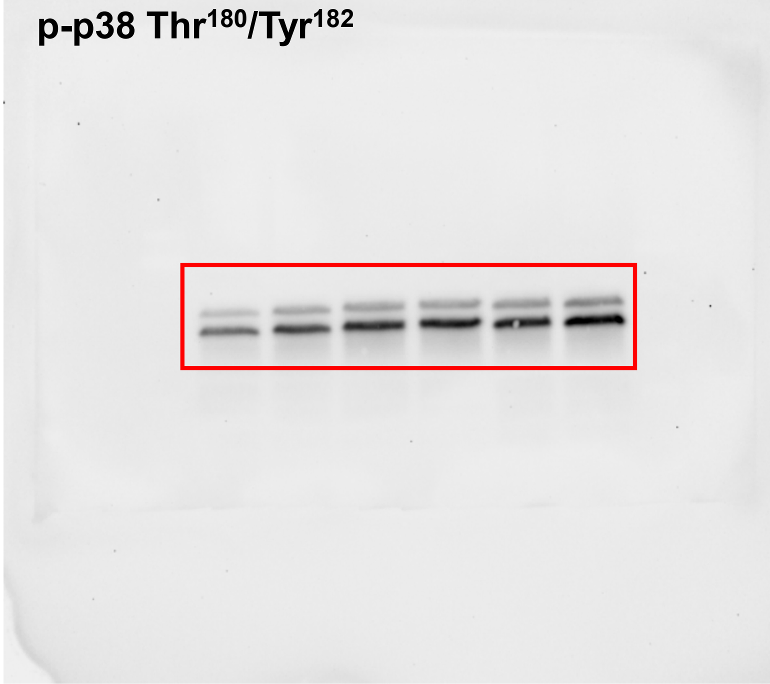

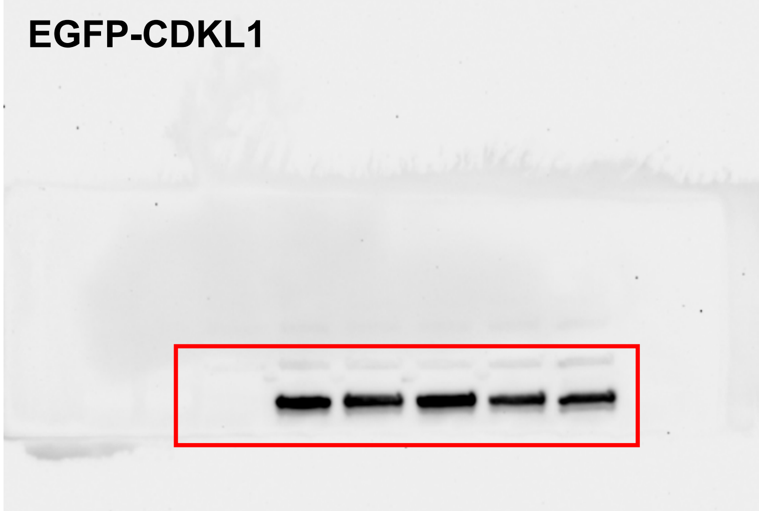

Note: EGFP signal in the first lane is missing because the western blot membrane was cut apart over the EGFP band in order to be able to detect other target proteins in parallel on the lower part of the membrane.

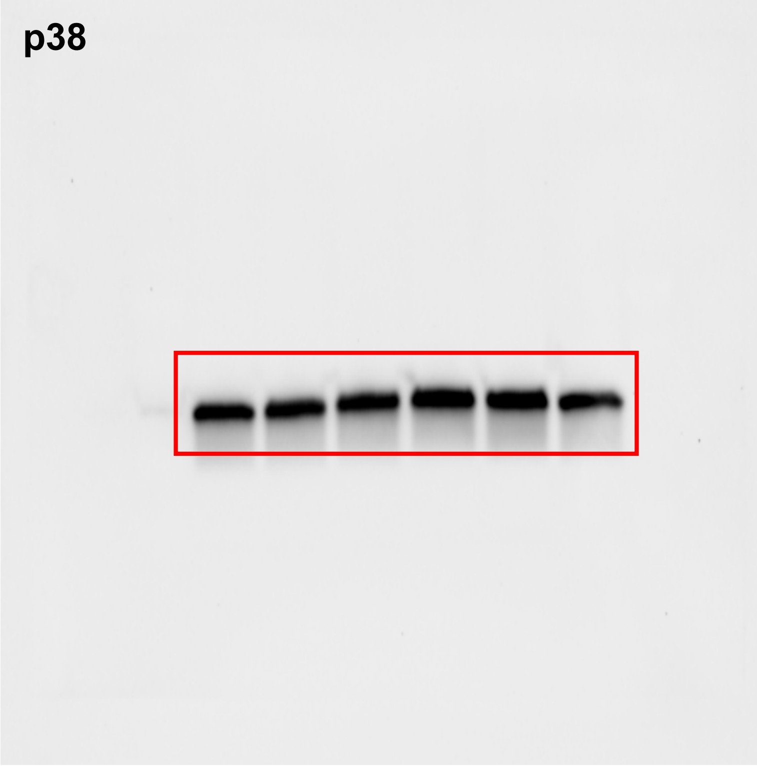

Supplement: Unedited blot and gel images [file jci-135-186287-s232.pdf]
